# High-density SNP-based genetic map development and linkage disequilibrium assessment in *Brassica napus* L

**DOI:** 10.1186/1471-2164-14-120

**Published:** 2013-02-22

**Authors:** Régine Delourme, Cyril Falentin, Berline Fopa Fomeju, Marie Boillot, Gilles Lassalle, Isabelle André, Jorge Duarte, Valérie Gauthier, Nicole Lucante, Amandine Marty, Maryline Pauchon, Jean-Philippe Pichon, Nicolas Ribière, Gwenn Trotoux, Philippe Blanchard, Nathalie Rivière, Jean-Pierre Martinant, Jérôme Pauquet

**Affiliations:** 1 INRA, UMR1349 IGEPP, BP35327, 35653, Le Rheu cedex, France; 2 Euralis Semences, Domaine de Sandreau, 31700, Mondonville, France; 3 Limagrain Europe, Route d’Ennezat, CS3911, 63920, Chappes, France; 4 BIOGEMMA, Domaine de Sandreau, 31700, Mondonville, France

## Abstract

**Background:**

High density genetic maps built with SNP markers that are polymorphic in various genetic backgrounds are very useful for studying the genetics of agronomical traits as well as genome organization and evolution. Simultaneous dense SNP genotyping of segregating populations and variety collections was applied to oilseed rape (*Brassica napus* L.) to obtain a high density genetic map for this species and to study the linkage disequilibrium pattern.

**Results:**

We developed an integrated genetic map for oilseed rape by high throughput SNP genotyping of four segregating doubled haploid populations. A very high level of collinearity was observed between the four individual maps and a large number of markers (>59%) was common to more than two maps. The precise integrated map comprises 5764 SNP and 1603 PCR markers. With a total genetic length of 2250 cM, the integrated map contains a density of 3.27 markers (2.56 SNP) per cM. Genotyping of these mapped SNP markers in oilseed rape collections allowed polymorphism level and linkage disequilibrium (LD) to be studied across the different collections (winter *vs* spring, different seed quality types) and along the linkage groups. Overall, polymorphism level was higher and LD decayed faster in spring than in “00” winter oilseed rape types but this was shown to vary greatly along the linkage groups.

**Conclusions:**

Our study provides a valuable resource for further genetic studies using linkage or association mapping, for marker assisted breeding and for *Brassica napus* sequence assembly and genome organization analyses.

## Background

Genetic linkage maps are highly valuable tools for comparative genome analyses and the identification of genomic regions carrying major genes and quantitative trait loci (QTL) controlling agronomical traits. They are a prerequisite for further map-based cloning or marker-assisted breeding programs. In recent years, the establishment of genetic maps have benefited from the development of new types of molecular markers which take advantage of automated sequencing and genotyping technologies. While the first marker-based genetic maps were built with restriction fragment length polymorphisms (RFLPs), random amplified polymorphic DNAs (RAPDs) and amplified fragment length polymorphisms (AFLPs), dense genetic maps now include simple sequence repeats (SSRs) and more recently single nucleotide polymorphisms (SNPs). Dense genetic maps based on sequence-derived markers allow finer comparative genome analyses to be performed based on comparisons with sequenced related genomes and to accelerate the process of map-based cloning of major genes and QTL. They are also very useful tools to assist sequence assembly in whole *de novo* genome sequencing projects [1-3]. Moreover, by integrating genetic map data with genotyping data generated from collections of accessions/varieties linkage disequilibrium (LD) pattern along the genome of a given species can be investigated, which is a prerequisite for precise genome wide association studies (GWAS). GWAS performed with a large number of SNPs have been reported in a number of crop species such as maize [4,5], Arabidopsis [6], barley [7-11], and rice [12-15] The success of GWAS to locate genes responsible for complex traits depends on the extent of LD, the number, the distribution and the diversity of markers and the underlying structure in the studied collections. Since the diversity of markers and the extent of LD may vary depending on the history of the collections [7,15], they should be investigated prior to GWAS design.

Oilseed rape (*Brassica napus*) is a prominent oilseed crop in most world continents including America, Europe, Australia and Asia and is cultivated for food (oil) and feed (meal) as well as for non-food uses such as biofuels or lubricants. It is the second world oil crop after soybean (http://faostat3.fao.org/home/index.html; May 2011) with a world production of more than 60 million tonnes per year. *B*. *napus* is an amphidiploid species (AC genome, n = 19) that arose from hybridization between *B*. *rapa* (A genome, n = 10) and *B*. *oleracea* (C genome, n = 9) diploid species [16] within the past 10,000 years [17]. *B*. *napus* includes spring and winter oilseed rape, rutabaga or swede, and some fodder crops. It likely originated from a few interspecific hybridization events [18] and has only a short domestication history of about 400–500 years [17,19]. For these reasons, the genetic diversity within *B*. *napus* germplasm is rather low compared to that of its two progenitor species *B*. *rapa* and *B*. *oleracea*. Moreover, two bottlenecks have occurred during breeding of modern oilseed rape varieties through the selection for low erucic acid content in the oil and low glucosinolate content in the seeds, which reduced the genetic diversity in modern varieties [20].

Over the last 20 years, many genetic *B*. *napus* maps have been built, which have been progressively integrating various types of markers [21-27]. These maps have been used for genetic studies of various agronomical traits including development traits [28,29], seed quality [30-34], yield components [35-38] and disease resistance [39-44] as well as for genetic study of chromosome pairing [45]. The establishment of genetic maps of diploid and amphidiploid Brassica species, and their comparison and alignment to Arabidopsis genome sequence provided insights into Brassica genome organization and evolution after the different rounds of polyploidization and diploidization occurring in these species history. Extensive collinearity observed between *A*. *thaliana* and *B*. *napus* led to the description of a genomic block system determined by Parkin *et al*. [21], who demonstrated that the structure of the *Brassica* A and C genomes could be described with approximately 21 conserved blocks. A framework built of 24 genomic blocks (A-X) within the ancestral karyotype was then proposed that represents an extension of the above mentioned study [46]. This conserved block structure was then further investigated in related species such as *B*. *juncea*[47] or *B*. *oleracea*[48] and led to a block arrangement comparison in the A, B and C genomes [49]. It was also recently confirmed in *B*. *napus* using dense genetic maps with SSR [27], and SNP [50] markers.

The availability of high numbers of markers now makes it possible to investigate more precisely genome wide diversity and the extent of LD in oilseed rape (OSR). To date, the published studies relied on either a low number of lines or low number of markers. Ecke *et al*. [51] used 845 AFLP markers to examine the extent of LD in a set of 85 winter oilseed rape lines. Bus *et al*. [20] investigated patterns of genetic diversity and the extent of LD in 509 inbred lines corresponding to different germplasms of oilseed rape with 89 SSR markers. Xiao *et al*. [52] assessed the genetic diversity and the extent of LD in a panel of 192 inbred lines from all over the world but with a great proportion originating from China using 451 SSR markers. Harper *et al*. [53] carried out associative transcriptomics on 53 *B*. *napus* lines using >60 K SNPs and confirmed the low overall level of LD in *B*. *napus*.

In this context, our objectives were to (i) obtain a dense integrated SNP genetic map of *B*. *napus* built from four segregating populations (ii) investigate the polymorphism of these SNPs within and among different germplasm types of a large *B*. *napus* collection and (iii) assess the extent and pattern of LD between densely mapped SNP markers.

## Results

### Map description

A total of 7322 SNPs was selected for Infinium genotyping according to the criteria described in methods section. Of these, 5986 were retained to build the four individual maps as they exhibited clear segregation patterns after Genome Studio analysis. The numbers of SNP markers mapped were 2664, 2763, 3385 and 2301 for the DYDH (‘Darmor-*bzh*’ × ‘Yudal’ doubled haploid), TNDH (‘Tapidor’ × ‘Ningyou7’ doubled haploid), AADH (‘Aviso’ × ‘Aburamasari’ doubled haploid) and AMDH (‘Aviso’ × ‘Montego’ doubled haploid) populations, respectively (Table 1). In addition, 833 and 831 PCR markers (SSR and sequence-derived markers) were mapped in the DYDH and TNDH populations. Individual maps covered 1947 cM for the TNDH and AMDH populations, 2049 cM for the DYDH and 3495 cM for the AADH ones and the numbers of markers per cM were 1.85, 1.18, 1.71 and 0.97, respectively (Table 1 and Additional file 1: Table S1). The percentage of mapped SNPs that showed segregation distortion was estimated at 46.2%, 28.3%, 16.1% and 16.3% in the DYDH, TNDH, AADH and AMDH populations, respectively. Most of the linkage groups in the DYDH and TNDH maps showed segregation distortion except A1, A4, A8, A10, C1, C4, C8 on DYDH and A1, C1, C2, C4, C5, C9 on the TNDH maps (Additional file 2: Table S2). On the AMDH map, only A2, A3, A9, C1 and C9 showed segregation distortion. Many regions were distorted in more than one map such as regions on A2, A3, A4, A9, A10, C3, C6, C7, C8 and C9.

**Table 1:** **Summary of the individual genetic maps obtained on the ‘Darmor- *bzh *’ x ‘Yudal’ (DYDH), ‘Tapidor’ x ‘Ningyou7’ (TNDH), ‘Aviso’ x ‘Abumasari’ (AADH) and ‘Aviso’ x ‘Montego' (AMDH) populations: number of markers and SNPs, map length (in cM), distribution of markers and SNPs per cM on the whole genome and on the genomesA and C**

	**DYDH**			**TNDH**			**AADH**			**AMDH**		
	**Whole**	**A**	**C**	**Whole**	**A**	**C**	**Whole**	**A**	**C**	**Whole**	**A**	**C**
Number of markers	3497	2159	1338	3594	2503	1091	3385	2350	1035	2301	1341	960
Number of SNPs	2664	1702	962	2763	1995	768	3385	2350	1035	2301	1341	960
Length (cM)	2049	982	1066	1947	967	980	3495	1789	1706	1947	892	1055
Number of markers/cM	1.71	2.25	1.25	1.85	2.59	1.15	0.97	1.97	0.62	1.18	1.57	0.88
Number of SNPs/cM	1.30	1.79	0.90	1.42	2.08	0.81	0.97	1.37	0.62	1.18	1.57	0.88

The integrated map comprised 7367 markers (5764 SNP and 1603 PCR markers) and covered 2250 cM, which corresponds to a density of 3.27 markers (2.56 SNPs) per cM (Table 2). Twice as many SNP markers were assigned a position on the A genome compared to the C genome: 3942 (68.4%) and 1822 (31.6%) were mapped on the A and C genomes, respectively, which correspond to 4.6 and 2.17 SNPs per cM. This difference between genomes A and C was less pronounced for the AMDH population. Of these 5764 SNP markers, 2350 (41%), 2150 (37%), 1112 (19%) and 152 (3%) were mapped in only one or were common to two, three or four populations, respectively. The AMDH population had the smallest number of markers (28%) in common with the other populations whereas 35 to 40% of the markers were common between the AADH, DYDH and TNDH populations. These percentages were similar if we consider the SNPs that were mapped to the A and C genomes, respectively.

**Table 2:** Number of markers and SNPs included in the integrated map, linkage group (LG) length, distribution of markers and SNPs per cM for each LG, total number of markers and SNPs on the A, C and whole genomes and ratio of marker and SNP number on the A genome relatively to the C genome (A/C)

**Integrated map**					
**LG**	**Number of markers**	**Number of SNPs**	**Length (cM)**	**Number of markers/cM**	**Number of SNPs/cM**
A1	533	425	120.1	4.44	3.54
A2	474	412	117.9	4.02	3.49
A3	664	504	137.5	4.83	3.67
A4	420	365	67.4	6.23	5.42
A5	578	493	106.4	5.43	4.63
A5	527	442	137.1	3.84	3.08
A7	458	370	109.7	4.18	3.37
A8	212	159	102.1	2.08	1.56
A9	557	416	133.2	4.18	3.12
A10	447	376	65.7	6.80	5.72
C1270	270	181	104.5	2.58	1.73
C2	312	236	126.5	2.47	1.87
C3	471	368	183.5	2.57	2.01
C4	312	220	132.9	2.35	1.66
C5	158	93	144.7	1.09	0.64
C6	257	192	111.2	2.31	1.73
C7	247	189	103.3	2.39	1.83
C8	208	149	119	1.75	1.25
C9	262	194	128.3	2.04	1.51
**Total**	**7367**	**5764**	**2250.9**	**3.27**	**2.56**
Total A	4870	3942	1097.1	4.60	3.76
Total C	2497	1822	1153.9	2.17	1.58
**A/C**	**2.0**	**2.0**	**1.0**	**2.1**	**2.4**

The recombination rate on the AADH map was higher than on the others (Additional file 3: Figures S1 and Additional file 4: Figure S2) as expected due to the mating scheme used to produce the population. This increase was relatively homogeneous across all the linkage groups, except for the bottom of A2 and the top of C2. An increase in recombination rate was also observed at the top of A3 on the TNDH map. Overall, very good collinearity was observed between all the maps with the exception of three inversions that were observed at the bottom of A2 on the TNDH map, at the top of C8 on the AADH map and at the bottom of C8 on the AMDH map. The AMDH population was the least polymorphic with the lowest number of markers and an overall lower marker density: many regions were not polymorphic at all or had a very scarce number of markers. Non-polymorphic regions were observed on almost all the linkage groups and in particular very low coverage was obtained for linkage groups A8, A10, C5 and C8.

**Figure 1:**
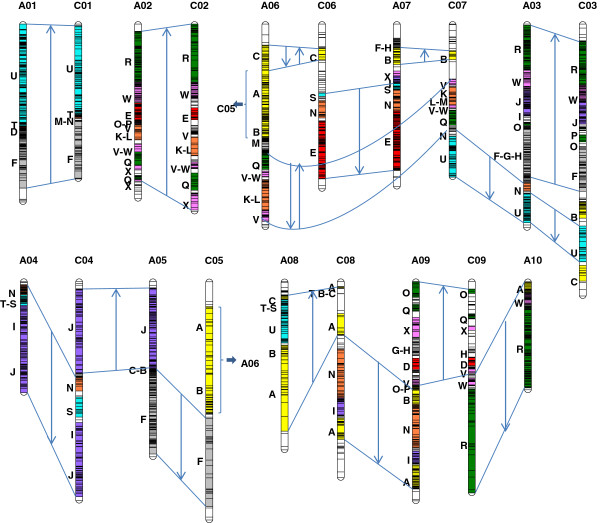
**Schematic representation of the integrated map.** The blocks as defined by Schranz et al. [46] based on their collinearity with *A*. *thaliana* are indicated with the capital letters A to X on the left of each linkage group. The A and C linkage groups are organized to show the collinearity between the homoeologous regions. Arrows in these homoeologous regions represent the orientation of the gene order (within the blocks) with respect to the corresponding regions in *A*. *thaliana*. The double arrows indicate an inversion of markers between two homoeologous regions.

We anchored the genetic maps onto the Arabidopsis genome using homology searches with the SNP context sequences and the previous anchorage method described in Wang *et al*. [27] for SSR and other PCR markers. Out of the 7367 mapped markers, 5725 gave hits with Arabidopsis genes. From these hits, 119 collinearity blocks were identified and represented in relation to the 24 blocks defined by Schranz *et al*. [46] (Figure 1). Since the collinearity between the different individual maps was very good, the conservation of the block organization between these individual maps was also very good with some additional blocks on some maps due to the different number of markers mapped in these regions (Additional file 3: Figure S1). However, few polymorphic markers were identified in some blocks especially on the AMDH map, which, as mentioned above, showed the lowest polymorphism rate. Indeed, on this map some blocks were totally or partially monomorphic (*e*.*g*. F on A1; E on A2 and C2; W, J and I on A3; V on A6; E on A7; B and A on A8; A on A9; W on A10; A and N on C8; R on C9). The A block was also partially monomorphic or missing on A8 of the DYDH map as well as the R block on A2 and C2 of the TNDH map.

In addition to these 7367 markers included in the integrated map, 222 markers were assigned to different linkage groups across the individual maps (Additional file 5: Table S3). Further investigation of these cases showed that these markers were included in the same synteny blocks and mapped to duplicated regions present on different linkage groups. Thus they appear to be homoeologous or duplicated loci.

### Validation of a 1536 SNP public set in a GoldenGate assay

A set of 1536 SNPs was selected for even distribution over the integrated linkage map and a good level of polymorphism in the OSR collection. The characteristics of these 1536 SNPs including their anchorage onto Arabidopsis and *B*. *rapa* sequences are described in Additional file 6: Table S4. Of these 1536 SNPs, 1104 (72%) were successfully genotyped on the Illumina BeadXpress platform. Twenty-nine could not be localized on the integrated map as they were assigned to different linkage groups on the individual maps. The distribution of the remaining 1507 SNPs, that were located on the integrated linkage map (Table 3), showed that the mean distances between two SNP markers were 1.32 and 1.79 cM for the original set and the GoldenGate validated set, respectively, which on average corresponded to a 35% increase. Consequently, the number of gaps over 10 cM increased from 22 to 32, out of 1488 and 1069 intervals, respectively in these two sets. Out of the 1536 SNPs, 1412 and 1527 showed a significant hit with Arabidopsis genes or *B*. *rapa* sequences, respectively. A total of 1461 SNPs had a significant hit on the *B*. *rapa* pseudo-chromosomes: 883 and 578 were located on the A and C linkage groups, respectively. Ninety-five percent of the SNPs located on A linkage groups showed a hit on the corresponding pseudo-chromosomes. Eighty-six percent of the SNPs located on the C linkage groups showed a hit on the expected pseudo-chromosomes according to the known collinearity between the A and C genomes.

**Table 3:** Distribution of the SNPs from the 1536 set and from the GoldenGate validated set over the linkage groups in the integrated map

		**1536 set**		**Validated in GoldenGate assay**			
**LG**	**Length**	**Number of SNPs**	**Mean distance between two SNPs**	**Number of gaps >10 cM**	**Number of SNPs**	**Mean distance between two SNPs**	**Number of gaps >10 cM**
A1	120.1	96	1.15	1	59	1.52	1
A2	117.9	82	1.10	1	72	1.40	1
A3	137.5	123	1.08		75	1.75	
A4	67.4	85	0.74		63	0.97	
A5	106.4	114	0.89	1	79	1.29	1
A6	137.1	98	1.26	1	74	1.68	3
A7	109.7	110	0.80		85	1.04	
A8	102.1	43	2.00	2	38	2.27	2
A9	133.2	71	1.78	1	48	2.60	3
A10	65.7	76	0.83		58	1.06	
C1	104.5	67	1.53	1	49	2.10	2
C2	126.5	56	2.08	1	33	3.49	2
C3	183.5	124	1.27		94	1.68	3
C4	132.9	82	1.53	2	63	2.00	2
C5	144.7	27	4.50	5	21	5.40	5
C6	111.2	73	1.14	1	59	1.27	1
C7	103.3	64	1.30	1	44	1.91	1
C8	119	47	1.97	1	33	2.78	1
C9	128.3	59	2.06	3	41	2.98	4
**Total**	**2250.9**	**1507**	**1.32**	**2**	**1088**	**1.79**	**32**

### Polymorphism in the *B. napus* collection

A total of 5685 SNPs were validated and scored on the *B*. *napus* collection. On average, only 1.1% of the data (0–10.3) was missing and 1.4% (0–21.9) was heterozygous (or of a mix of homozygous and heterozygous) per variety. Only 13 varieties showed more than 10% of heterozygosity. A total of 4881 SNPs with a minor allele frequency (MAF) greater than 5% on the whole collection was retained for further polymorphism study and analysis of molecular variance (AMOVA). An AMOVA was carried out to assess genetic differentiation between fodder, spring oilseed rape and winter oilseed rape varieties, and between the three seed quality subgroups (“++”, “0+” and “00”) within the spring and winter oilseed rape types. The level of within subgroup variation was 72.5 and 68.7% whether we considered fodder varieties or not, with F_ST_ indices of 0.275 and 0.312 and differentiation indices between types of 0.181 and 0.223, respectively. The F_ST_ indices were 0.089 and 0.170 within winter and spring oilseed rape types, respectively.

**Figure 2:**
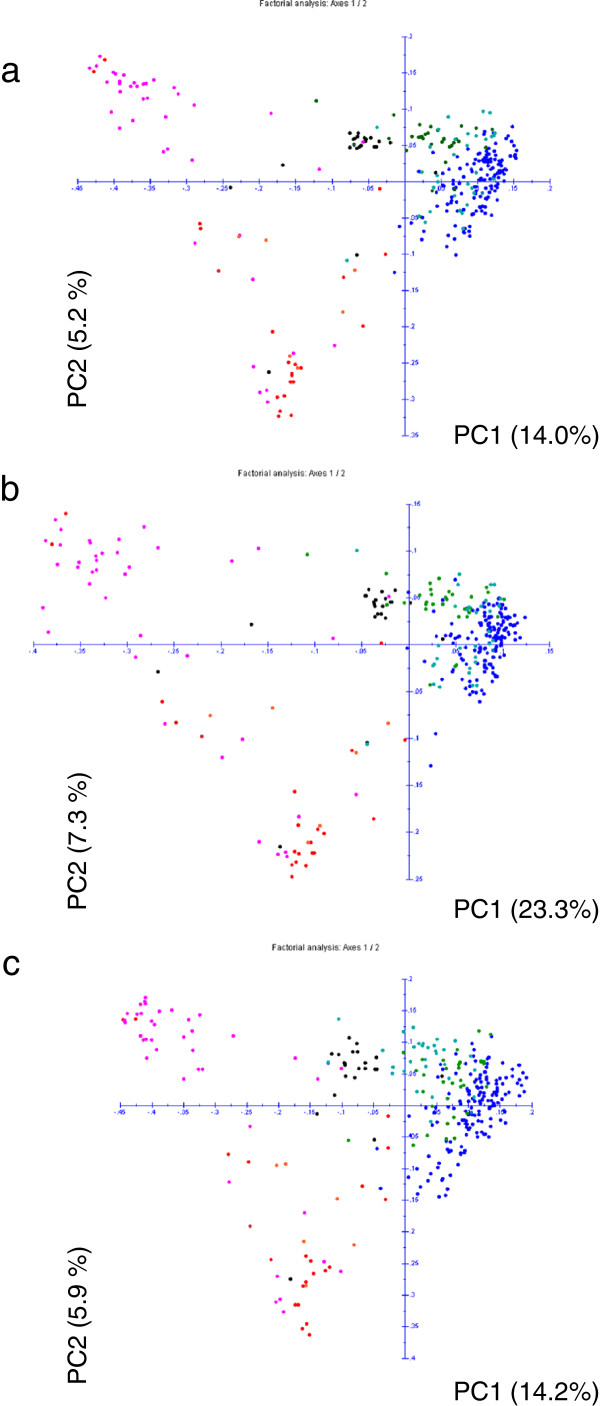
**Principal component analyses (PCA) of 313 *B*. *napus *varieties based on simple matching distances calculated from the total 4363 SNP loci (a), the 2854 SNP loci mapped on the A genome (b) and the 1509 SNP loci mapped on the C genome (c).** PC1 and PC2 are the two first principal coordinates and the proportion of variance explained by these coordinates is indicated in parentheses. Black color states for fodder rape, magenta for Asian spring oilseed rape (OSR), red for European and Canadian spring OSR, dark blue for « 00 » winter oilseed rape (WOSR), light blue for « 0+ » WOSR and green for « ++ » WOSR.

Of the 4881 SNPs above, 4363 were localised on the integrated map and were further used for principal component analyses (PCA). Three PCAs were performed using either i) the whole set of mapped SNPs; ii) the 2854 SNPs that were mapped on the A genome or iii) the 1509 SNPs that were mapped on the C genome. The first two axes accounted for 19.2%, 30.6% and 20.1% of the variation, respectively in these three PCAs (Figure 2). Out of the 1536 SNPs selected subset, 1507 SNPs were located on the integrated map. In the PCA performed with these 1507 SNPs or with the 908 and 599 SNPs mapped on the A and C genomes, respectively, the first two axes accounted for 18.5%, 18.3% and 20.0% of the variation. In each case, the first axis differentiated the spring oilseed rape from the winter oilseed rape accessions and the fodder rape varieties were between the two groups. The second axis mainly discriminated European and Canadian (“0+” and “00”) spring oilseed rape from Asian spring “++” oilseed rape. No clear differentiation between “++”, “+0” and “00” winter oilseed rape subgroups was observed.

The 4363 SNPs that were localised on the integrated map were further used for investigating polymorphism variation across the linkage groups. Table 4 and Additional file 7: Figure S3 show the mean polymorphism information content (PIC) values for each linkage groups in the whole collection, the fodder, spring and winter oilseed rape types, as well as in the “++”, “0+” and “00” winter oilseed rape subgroups. The polymorphism level was significantly higher in spring oilseed rape than in fodder and winter oilseed rape and was significantly lower in winter oilseed rape than in fodder rape. The exception was the C1 linkage group where the polymorphism was much lower in the spring oilseed rape types. On average, the polymorphism level was slightly lower for the A than for the C linkage groups except for the spring oilseed rape types and there was a great variability between linkage groups. A1, A2, A9 and A10 were the least polymorphic linkage groups for the A genome, especially in winter oilseed rape. For the C genome, C2, C8, C9 and C2, C9 were the least polymorphic in winter oilseed rape and fodder types, respectively. For winter oilseed rape, the polymorphism level was quite similar between the “++”, “0+” and “00” subgroups for the A linkage groups whereas there was a greater variability for the C linkage groups with some linkage groups such as C2, C4, C5, C9 showing lower PIC values in the “0+” and “00” subgroups and other linkage groups such as C1, C3, C7 showing higher PIC values in the “00” subgroup. The mean PIC values were also estimated for the whole collection with the mapped SNPs from the 1536 SNP subset. These were significantly higher than the ones estimated with the full SNP set (Table 4), due to the criteria used for their selection.

**Table 4:** Polymorphism Information Content (PIC) estimated for the whole collection, on the fodder, the spring (SOSR) and winter (WOSR) oilseed rape types and on the three seed quality subgroups within WOSR

				**Full set**				**1536 set**
**LG**	**Fodder rape**			**“++”**	**“+”**	**“00”**	**Whole Collection**	**Whole Collection**
		**SOSR**	**WOSR**	**WOSR**	**WOSR**	**WOSR**		
		**(24)**	**(68)**	**(221)**	**(33)**	**(33)**	**(154)**	**(313)**	**(313)**
A1	0.220	0.313	0.188	0.208	0.189	0.175	0.259	0.307
A2	0.236	0.293	0.190	0.175	0.173	0.187	0.254	0.287
A3	0.260	0.309	0.229	0.243	0.225	0.216	0.286	0.322
A4	0.253	0.297	0.215	0.213	0.199	0.207	0.272	0.307
A5	0.261	0.297	0.233	0.218	0.231	0.221	0.277	0.312
A6	0.260	0.303	0.232	0.233	0.230	0.218	0.272	0.307
A7	0.258	0.301	0.241	0.248	0.227	0.232	0.285	0.313
A8	0.256	0.296	0.265	0.225	0.248	0.253	0.296	0.325
A9	0.219	0.316	0.183	0.162	0.160	0.165	0.247	0.305
A10	0.243	0.310	0.177	0.203	0.166	0.164	0.259	0.292
**Mean A genome**	**0.247**	**0.304**	**0.214**	**0.214**	**0.204**	**0.202**	**0.270**	**0.308**
C1	0.282	0.220	0.296	0.263	0.247	0.299	0.319	0.322
C2	0.225	0.304	0.209	0.233	0.159	0.184	0.283	0.304
C3	0.247	0.314	0.268	0.210	0.216	0.270	0.300	0.308
C4	0.294	0.301	0.243	0.273	0.234	0.226	0.297	0.310
C5	0.288	0.275	0.257	0.284	0.258	0.243	0.305	0.330
C6	0.312	0.298	0.271	0.282	0.298	0.244	0.309	0.315
C7	0.270	0.287	0.272	0.238	0.230	0.270	0.298	0.323
C8	0.270	0.302	0.215	0.232	0.225	0.202	0.279	0.313
C9	0.223	0.270	0.234	0.245	0.225	0.208	0.290	0.301
**Mean C genome**	**0.265**	**0.290**	**0.253**	**0.246**	**0.229**	**0.241**	**0.298**	**0.313**
**Whole genome**	**0.253**	**0.299**	**0.227**	**0.225**	**0.213**	**0.216**	**0.280**	**0.310**

Mean PIC values estimated on the A, C and whole genomes are given for each subgroup of the collection and on the whole collection with the full set of SNPs or the 1536 SNP subset. The number of accessions in each subgroup is indicated in parentheses.

Additional file 7: Figure S3 shows that the PIC values varied differently along the different linkage groups. When we compared fodder rape, spring oilseed rape (SOSR) and winter oilseed rape (WOSR), there were large regions on most A linkage groups except A8 where the level of polymorphism was higher in the spring oilseed rape types. The polymorphism level in winter oilseed rape was either lower or similar to the one observed in fodder rape, depending on the A regions. On the C linkage groups, the level of polymorphism was lower in some regions in winter oilseed rape *e*.*g*. on C2, C4, C6 and C8 linkage groups but elsewhere the variation in PIC values between the different types was more erratic. The C1 and to a lesser extent the C5 linkage groups showed a contrasted situation with a lower polymorphism in spring oilseed rape along most of their length. When we compared the three winter oilseed rape subgroups (“++”, “0+” and “00”), variation in PIC values along the different linkage groups was very contrasted since it decreased or increased in the “0+” and/or “00” subgroups depending on regions. Nevertheless, regions showing a decrease in polymorphism were more numerous.

### LD mapping

The 4329 SNPs that were localised on the integrated map and had a MAF above 5% were then used for mean LD estimation and LD pattern study along the different linkage groups depending on the oilseed rape types. The whole collection or spring, winter and “00” winter oilseed rape were considered so that a sufficient number of varieties was included to estimate the LD.

The mean pairwise r^2^ was estimated at 0.037, 0.057, 0.017 and 0.021 in the whole, the spring, the winter and the “00” winter oilseed rape collections, respectively. This corresponded to 2.9%, 6.6%, 0.4% and 0.7% of the SNP pairs showing a r^2^ value higher than 0.2 (Table 5). In the whole collection and the spring types, a high percentage of the pairs with a significant LD were between SNP markers located on different chromosomes (85-90%) whereas in the winter types, most were intra-chromosomic pairs. The r^2^ value was greater than 0.5 for 0.12% to 0.25% of the pairs and greater than 0.8 for less than 0.1% of the pairs. Very few pairs with r^2^ > 0.8 were observed between SNP markers located on different chromosomes.

**Table 5:** **Number and percentage of pairs exhibiting r**^**2**^ **> 0.2, 0.5 or 0.8 between SNPs located within a same linkage group (intra LG) or on different LGs (inter LG)**

	**Whole collection**		**SOSR collection**		**WOSR collection**		**“00” WOSR collection**	
	**number**	**%**	**number**	**%**	**number**	**%**	**number**	**%**
SNP	4329		4137		3409		3196	
Pairs	9367956		8555316		5808936		5105610	
Pairs with r^2^ > 0.2	274421	2.929	563609	6.588	23840	0.410	33656	0.659
intra LG	42879	0.458	56480	0.660	20474	0.352	25852	0.506
inter LG	231542	2.472	507129	5.928	3366	0.058	7804	0.153
Pairs with r^2^ > 0.5	15194	0.162	21390	0.250	7085	0.122	9075	0.178
intra LG	9032	0.096	9553	0.112	6950	0.120	8944	0.175
inter LG	6162	0.066	11837	0.138	135	0.002	131	0.003
Pairs with r^2^ > 0.5	2908	0.031	2678	0.031	2890	0.050	3820	0.075
intra LG	2901	0.031	2620	0.031	2877	0.050	3805	0.075
inter LG	7	0.000	58	0.001	13	0.000	15	0.000

**Figure 3:**
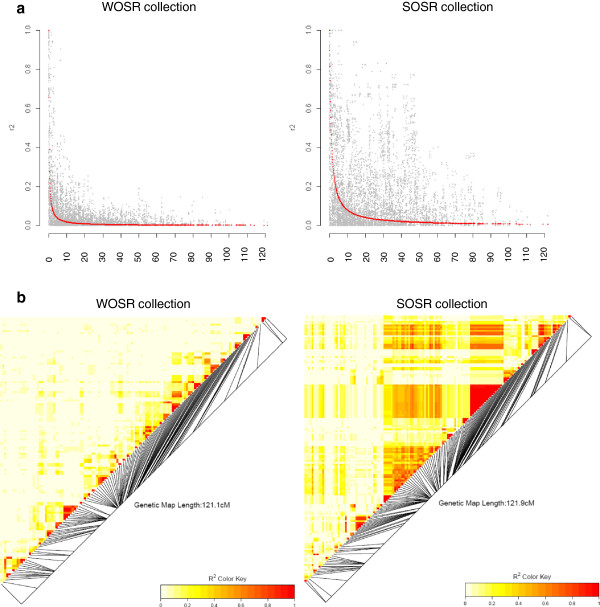
**Plots of r**^**2 **^**as a function of genetic distance (in cM) between pairs of SNPs (a) and linkage disequilibrium heatmaps (b) for linkage group C9 in the winter (WOSR) and the spring (SOSR) collections.**

LD decay was estimated globally and for each linkage groups from the four collections. The non-linear regression of the LD measure (r^2^) relative to genetic map distance and the genetic distance at which the estimated r^2^ fell below 0.2, as well as the effective size, were estimated (Table 6). The trend lines of these non-linear regressions are shown in Additional file 8: Figure S4. The genetic distance at which the estimated r^2^ fell below 0.2 was 0.6-0.7 cM for the whole collection, the spring and winter types whether it was estimated on the whole genome or the A and C genomes. However on the C genome for the spring types, this distance was estimated at 1.2 cM. For the “00” winter types, it was estimated at 1.2 cM on the whole genome as well as on the genomes A or C. This value varied depending on the linkage group and the collection, ranging from 0.2 to 3.4 cM. On all the linkage groups, the extent of LD was overall higher for the “00” winter types than for the winter types that included the “++”, “0+” and “00” varieties. Different linkage groups showed a higher extent of LD depending on whether it was estimated from the spring or the winter types *e*.*g*. A9, C2 and C9 for the spring types and A2, A6, A8 and C6 for the winter types. The effective size varied accordingly. The very different LD patterns between spring and winter types was also evident from the LD plots obtained for each linkage group. This was true for the linkage groups cited above such as for C9 (Figure 3) but some other differences could be seen on most of the groups (Additional file 9: Figure S5).

**Table 6:** **Genetic distance (d) and effective size (Ne) at which r**^**2 **^**fell below 0.2 estimated for each linkage group and globally for A, C and whole genomes on the whole, SOSR, WOSR and “00” WOSR collections**

	**Whole collection**		**SOSR collection**		**WOSR collection**		**“00” WOSR collection**	
	**d**	**Ne**	**d**	**Ne**	**d**	**Ne**	**d**	**Ne**
Whole genome	0.6	161	0.7	138	0.7	137	1.2	82
A genome	0.6	165	0.6	165	0.7	139	1.2	82
C genome	0.6	161	1.2	80	0.7	137	1.2	82
A1	0.8	124	0.9	106	0.6	159	1.0	95
A2	0.8	113	0.6	151	1.4	69	2.1	47
A3	0.7	127	0.7	126	0.5	169	0.8	115
A4	0.3	284	0.3	257	0.4	231	0.7	128
A5	0.4	239	0.5	195	0.8	121	1.2	83
A6	0.4	224	0.5	199	1.1	87	2.3	42
A7	0.3	227	0.4	249	0.4	220	1.0	98
A8	0.9	103	0.8	116	1.9	51	2.8	34
A9	1.6	61	1.7	57	0.8	109	1.1	85
A10	0.4	212	0.2	360	0.4	227	0.7	140
C1	0.7	139	1.0	94	0.7	130	1.0	98
C2	1.7	58	2.6	37	0.9	108	1.3	72
C3	0.5	262	0.9	106	0.3	275	0.4	211
C4	0.3	262	0.9	106	0.3	275	0.4	211
C5	0.4	210	0.6	143	1.3	74	2.5	60
C6	0.4	210	0.6	143	1.3	74	2.5	40
C7	0.4	207	0.8	115	0.7	128	1.4	67
C8	0.4	214	0.9	104	0.5	189	0.7	137
C9	1.0	92	3.4	29	0.7	131	1.0	91

## Discussion

In this study, we could built a high density SNP integrated *B*. *napus* map and depict polymorphism level and LD decay over the linkage groups across different *B*. *napus* collections by integrating genotyping data of a large set of SNPs in both segregating populations and diverse collections.

As reviewed by Kaur *et al*. [54], SNP discovery is challenging in allopolyploid species such as *B*. *napus*. SNPs may arise both between allelic (homologous) sequences within subgenomes and between homoeologous sequences among subgenomes but also from polymorphisms between paralogous duplicated sequences. SNP discovery has been based on *B*. *napus* ESTs sequence analysis [55-57] or on second generation high throughput sequencing [50,58-60]. SNP genotyping using Illumina GoldenGate assays was shown to be possible in *B*. *napus* species after careful selection of the SNP [55]. Here, we report the first high throughput genotyping study in oilseed rape using Illumina Infinium and GoldenGate technology. From the 1536 SNPs tested on the two platforms, 1104 were validated on both, suggesting that it should be possible to use one platform or the other depending on the required number of SNPs.

In this study, we generated an integrated map with 7367 markers including 5764 SNPs, which corresponds to 3.3 markers every cM. This large number of mapped markers was obtained by integrating four mapping populations. Marker density actually increased from one marker (one SNP) every 0.54 (0.7-0.8) cM on the individual maps to one marker (SNP) every 0.3 (0.39) cM on the integrated map. This density was comparable to that obtained in Wang *et al*. [27] (one marker every 0.34 cM) but the number of markers in common between populations was much higher in our study (60% of the markers were common to at least two maps in our study compared to only 20% in Wang *et al*. [27]). The size of the population was also larger in our study, which led to a more accurate ordering of the markers on the integrated map. Bancroft *et al*. [50] built a *B*. *napus* map with 23027 SNPs by transcriptome sequencing of 37 TNDH lines. These SNP were distributed in 527 recombination bins (one bin corresponded to SNPs having the same scoring genotyping data). Our integrated maps exhibited 3177 bin loci (one bin corresponded to SNPs mapped within 0.1 cM), thus the mapping resolution increased considerably with the increase in population size. The collinearity blocks we identified were compared to those reported in Panjabi *et al*. [47], Parkin [49], Wang *et al*. [27] and Bancroft *et al*. [50]. Compared to Bancroft *et al*. [50], which is at present the most complete study, five and 10 small blocks were missing on the A and C genomes, respectively. Nevertheless, three new blocks were identified in our study: S on A8, V-W on C2 and B on C7. In Bancroft’s study, two markers corresponding to these blocks were identified but not declared as a block. Moreover, the V-W and B blocks were identified in the homoeologous regions on A2 and A7 linkage groups, which supports their occurrence in these genomic regions.

A higher number of markers was mapped on the A genome than on the C genome, as previously reported by Bancroft *et al*. [50]. As in their study, here, this difference between the A and C genomes was more pronounced on the three crosses involving Asiatic parental lines. The hypothesis is that Asiatic cultivars are partly derived from crosses involving its progenitor species *B*. *rapa*[61,62], which increased the genetic diversity of the A genome. This introgression of *B*. *rapa* genetic information was recently shown for ‘Nignyou7’ (one of the parent of the TNDH map) by Bancroft *et al*. [50]. Indeed, there was less difference between the number of markers mapped on the A and C genomes in the AMDH population which is derived from a cross between two French winter OSR varieties. No such difference was observed in Wang *et al*. [27] but the markers mapped in their study were mainly contributed by two crosses involving resynthetised *B*. *napus*, which is enriched in polymorphisms in both genomes. We probably succeeded in capturing this high level of polymorphism on the A genome because the original sequencing to identify the SNP was performed on material that included Asiatic varieties.

We observed a high level of segregation distortion especially in the DYDH and TNDY populations. Such segregation distortions were reported in many *B*. *napus* maps (*e*.*g*. [27]). Segregation distortion and clustering of the skewed loci are common features of microspore-derived DH populations in various species ([63] for a review), including oilseed rape and may be related to differential responsiveness to microspore culture between the two parental lines, which leads to skewed loci in regions involved in the microspore culture responsiveness.

Very good collinearity was observed between all the individual maps, which made it easy to integrate the four individual maps accurately. Only three inversions were identified on the A2 and C8 linkage groups. These inversions could be due to mapping inaccuracies and need to be confirmed. Due to careful selection at the beginning of the study to target homologous SNPs, very few SNPs were not assigned a position on the same linkage groups among the different maps. Those SNPs that did map to different linkage groups were located in duplicated regions within or between the A and C genomes where there is a high level sequence similarity. The map derived from the AADH population was 75% bigger than those derived from the other crosses, which was expected from the way the DH population was obtained. DH lines were produced after intermating F2 plants, which increased the number of recombination events. This type of highly recombinant population is of interest for obtaining better mapping resolution [64,65]. The map derived from the AMDH population was the least dense with some regions missing, due to the lack of polymorphism between the two parental lines. In many cases, the monomorphic regions corresponded to quite complete *B*. *napus*/Arabidopsis collinearity blocks. The lower marker density can be related to the lower level of polymorphism revealed within winter oilseed rape or “00” winter oilseed rape compared with spring oilseed rape.

A moderate level of differentiation was observed between the different *B*. *napus* types (WOSR, SOSR, fodder rape) as revealed by the estimated differentiation indices (0.18 between the three groups and 0.22 between SOSR and WOSR). A similar result was reported by Bus *et al*. [20]. They used 89 SSR primer combinations to assess the diversity in a set of 509 oilseed rape lines which included WOSR, SOSR, fodder and swede rape lines from diverse origins. Xiao *et al*. [52] found a lower level of differentiation between their groups defined from a collection of 192 oilseed rape lines genotyped with 451 SSR markers. However, each of their groups, as defined by STRUCTURE analysis, was constituted of lines from China, Europe, Canada and Australia. Within the SOSR and WOSR groups, a very low level of differentiation was observed between the three subgroups which corresponded to the three quality types (“++”, “0+” and “00”) with 91% and 83% of the variation present within the subgroups in WOSR and SOSR, respectively. Examination of the collection structure with PCA showed distinct clustering of WOSR, SOSR and fodder rape lines while the first two axes did not account for a large part of the variation (19.5%). This differentiation was previously reported by Diers and Osborn [66], Hasan *et al*. [67] and Bus *et al*. [20] and can be related to the relatively distinct breeding history between these pools and their adaptation to different environments or uses. However, our data allowed two groups to be differentiated within SOSR which mainly contained European and Canadian or Asian spring oilseed rape, thus corresponding to a differentiation due to geographic origin. Some SOSR lines were located at an intermediate position between these two groups such as ‘Grouse’ and ‘Marnoo’ (Australian cvs), ‘Chine Wuhan’ and ‘Yeong Dang’ (Asiatic cvs) or ‘Industry’ (a European cv with high erucic acid and low glucosinolate content). No such differentiation was observed within the WOSR lines which all originated from Europe, as previously reported by Ecke *et al*. [51] and Bus *et al*. [20]. Fodder rape lines were located at an intermediate position between SOSR and WOSR although closer to WOSR than to SOSR. The exception was ‘Liho’ which is a spring fodder rape and grouped with the SOSR. This result is consistent with the fact that fodder types and oilseed types were derived from the same ancestral spring and winter pools and were bred for different uses, as reported by Bus *et al*. [20]. The same structuration was obtained whether we consider SNPs from the A or the C genomes. The percentage of variation accounted for by the first two axes was higher with the SNPs from the A than from the C genome when we used the whole SNP set where the markers from the A genome were overrepresented. When we considered the 1507 chosen SNP markers, the percentage of variation accounted for by the first two axes was similar with the SNPs from both genomes, indicating that an overrepresentation of markers in some regions can biased the results. A selection of evenly spaced markers over all the linkage groups is thus recommended for genetic structuration. The optimal number has to be assessed by testing different sample sizes.

To assess the genetic diversity in the collection and within the different germplasm groups, we examined the mean PIC and its evolution along each linkage group. The PIC values ranged between 0.1 and 0.35 depending on the position on the linkage groups and depending on the collections used. Similar observations were made in barley and maize with the same type of markers [4,10,68]. Our set of SNPs was only derived from exonic sequences, which could have lowered the level of revealed diversity compared to intronic SNPs [4]. On average, PIC values were lower in WOSR than in SOSR. This difference was more important for SNPs derived from the A genome rather than the C genome. This might be because the SOSR lines have more diverse geographic origins than the WOSR lines and have undergone differential selection to adapt to the different continents. In WOSR, the mean PIC values were not very different between the three seed quality types. The mean PIC value was only slightly higher in “++” WOSR type but this result should be taken with caution due to the different sizes of the three subgroups. Bus *et al*. [20] reported a lower genetic diversity for “00” seed quality WOSR varieties and the difference was also quite low. The variation in PIC values along the different linkage groups between the three WOSR seed quality types was very contrasted, with more numerous regions showing a PIC decrease for the “0+” and/or “00” types. These variations could be related to potential selection signatures within and between these types. In barley, specific chromosomal regions exhibited contrasting levels of diversity in different germplasm subgroups. A region of reduced diversity in winter barley in the central part of chromosome 5H was attributed to the small number of founding genotypes that contributed to the winter seasonal growth habit locus *Hrn*-*1*. Similarly, an abrupt decrease in diversity on the short arm of chromosome 3H observed in all groups coincided with the locus of non-shattering of ears after ripening [68]. We therefore investigated whether the regions with reduced diversity in either the “0+” and /or the “00” subgroups could be related to the position of genes controlling erucic acid and glucosinolate content. Erucic acid genes are located on A8 and C3. Numerous genes are involved in the glucosinolate pathway [69,70] but the major QTL controlling total glucosinolate content are located on A2, A9, C2, C7 and C9 [71,72], Delourme, unpubl. data. Their positions are indicated in Additional file 2: Table 2. However, there was no decrease in diversity clearly surrounding erucic acid genes in the “0+” and the “00” subgroups or total glucosinolate QTL in the “00” subgroup. The only exception could be at the top of C9. It can be hypothesized that many recombination events have occurred around the selected genes in these regions during different rounds of intercrossing between the varieties since the original crosses were made with the genitors of low erucic and low glucosinolate content. Significant decreases in diversity were observed in other regions, which could be related to breeding for other agronomical traits but a more precise investigation of QTL located in these regions should be made before drawing any conclusions.

The mean pairwise r^2^ values are close to previous estimates of 0.027 [51] or 0.0247 [53] and confirms the low overall level of linkage disequilibrium in *B*. *napus*. LD was observed to decay below a critical level (r^2^ value 0.2) within a map distance between 0.6 and 1.2 cM among the subgroups. This value is in accordance with previous studies performed either on smaller oilseed rape collections or with a smaller number of markers [20,52]. This level is lower than that detected in a collection of 85 WOSR lines genotyped with 845 AFLP markers, where the LD decayed within 2–3 cM at r^2^ < 0.2 [51]. This could be due to the fact that this latter collection comprised only “00” seed quality types. In our study, the extent of LD was also higher in the “00” WOSR collection than in the whole WOSR or the SOSR collections. From the size of the genetic maps and of the genomes in Brassica species, on average 1 cM can be roughly estimated to correspond to ~500 kb [51,73]. This means that on average for the whole genome, the extent of LD is between 300–1000 kb depending on the collections but great variations were observed across the linkage groups (from ~100 kb on A10 to ~1700 kb on C9 in SOSR). In many species, LD decay varied across the germplasms used. For example, LD extends less than 1 kb for maize landraces [74] and roughly 2 kb for diverse inbred maize lines [75] but can be as high as 100 kb for commercial elite inbred lines [76]. LD decay can also vary considerably from locus to locus 1–4 kb [77] up to 800 kb [78]. Similar differences were observed in rice, 50–500 kb, [12,15,79], Arabidopsis, 10–250 kb [80,81] or barley, 90–210 kb [82] or 4–8 cM [83]. Generally, the extent of LD is related to the mating system of the species, the breeding history of the species (*e*.*g*. the occurrence of bottlenecks) and the genetic diversity of the different germplasms [84]. LD decay is more rapid in outcrossing species and in pools with higher genetic diversity. Oilseed rape is bred as a selfing species and has undergone two bottlenecks for seed quality improvement (to eliminate erucic acid and decrease glucosinolate content), which led to a LD decay similar to other selfing species and to a higher LD extent in the “00” WOSR collection.

The LD patterns varied greatly among the linkage groups and these variations were different between the SOSR or WOSR types. Similar results were reported in maize [4,5], barley [85] or rice [13]. Different patterns of LD along the chromosomes in various pools can be related to variation in recombination rate and in the history of recombination for specific chromosome regions within these pools. The centromeres were localised approximately on the integrated map by mapping centromeric markers developed by Pouilly *et al*. [86] to the DYDH map. These were then included on the integrated map and on the LD heatmaps (Additional file 2: Table S2 and Additional file 9: Figure S5). On many linkage groups, LD seems to be extended across the centromeric regions as reported in barley [10] but other regions which do not correspond to centromeres also showed extended LD such as on A2, A8, C8 or C9. Differences in allele frequencies [87] could have also influenced the distribution of LD along the chromosomes. In barley, larger LD extent in some chromosomic regions was caused by markers with low allele frequencies [85]. In rice, LD decay rates in *indica* and *japonica* subspecies were only weakly correlated across the genome in relation to a relatively long history of partial reproductive isolation of these self-fertilized subspecies [13]. Since SOSR and WOSR have a relatively distinct breeding history, a similar hypothesis can also be proposed in our case.

## Conclusion

With high throughput SNP genotyping on four segregating DH populations, we developed an integrated genetic map for oilseed rape that comprises 5764 SNP and 1603 PCR markers. This significantly improves the marker density or mapping accuracy compared to previously published genetic maps. The genotyping of these mapped SNP markers in collections allowed polymorphism level and linkage disequilibrium to be studied in oilseed rape. Both were shown to vary across the different collections (winter *vs* spring, seed quality types) and across the linkage groups. Taking into account the length of the genetic map (~2500 cM) and the mean LD extent (0.7 – 1.2 cM for r^2^ > 0.2), a relatively low number of evenly spaced SNPs (few thousands) would be necessary to perform genome wide association studies in oilseed rape. However, this number should be adjusted to obtain a sufficient SNP density throughout the genome and to take into account the variation in LD along the linkage groups. A set of 1536 public SNPs was set up, of which 72% were validated on a GoldenGate platform. They provide evenly spaced SNPs showing a good level of polymorphism in oilseed rape. Information regarding the other SNPs can be requested from J Pauquet (Jerome.Pauquet@biogemma.com). Our study provides a valuable resource for further genetic studies through linkage or association mapping, marker assisted breeding and *Brassica* sequence assembly and comparative mapping.

## Methods

### Materials

Four doubled haploid (DH) *B*. *napus* populations were used. Two have already been described in previous studies: DYDH [23,31] and TNDH [33]. Sets of 280 and 94 DH lines were used for these two populations, respectively. The third population, referred to as the AMDH population, was derived from the cross between two French winter oilseed rape varieties ‘Aviso’ and ‘Montego’ and consisted in 87 DH lines produced from the F1 between these two parents. The fourth population, referred to as the AADH population, was derived from the cross between a French winter oilseed rape ‘Aviso’ and a Japanese oilseed rape ‘Aburamasari’ and consisted in 96 DH derived from 192 intermated F2 plants: each F2 plant was used once, as male or female, in a cross with another F2 plant so that 96 hybrids were generated and one DH was derived per hybrid.

A *B*. *napus* collection of 313 inbred lines from different geographical origins was used for diversity analyses and linkage disequilibrium assessment. It consisted of 65 spring oilseed rape (SOSR) lines, 223 winter oilseed rape (WOSR) lines and 25 fodder rape lines from Europe, Australia, Asia and Canada. The SOSR and WOSR groups were divided in three subgroups depending on their seed quality types: “++” for high erucic acid and glucosinolate content, “0+” for low erucic acid and high glucosinolate content and “00” for double low types. A description of this collection is presented in Additional file 10: Table S5.

### SNP origin and selection

Two sets of SNPs were used. The first set was obtained in previous internal research programs in Biogemma using sequence capture technology. Publicly available Brassica ESTs contigs (http://brassica.nbi.ac.uk/array_info.html) corresponding to a wide range of gene function were used to capture the corresponding genomic DNA in OSR genotypes including ‘Aviso’, ‘Montego’ and ‘Aburamasari’ as parent of mapping populations and Asiatic lines [88,89]. A custom 2.1 M probes sequence capture Nimblegen (Roche NimbleGen, Inc., Madison, USA) microarray designed from those contigs was used with a protocol adapted from Albert *et al*. [90]. Briefly, 454 sequencing libraries were synthesized, hybridized on microarray and subsequently, specifically hybridized library fragments were eluted and sequenced on a 454 GS-FLX sequencer. Reads were mapped against the targeted contigs and then assembled within each cluster using MIRA software [91] and SNP detection was performed using stringent criteria based on base quality, absence of heterozygosity within genotypes and 2X minimal allele coverage. The second set corresponds to SNPs identified between ‘Tapidor’ and ‘Ningyou7’ [92]. All the SNPs were submitted to the Illumina Assay Design Tool (ADT) (Illumina, San Diego, CA), and only SNPs with designability scores > 0.4 for both Infinium and GoldenGate chemistries were included in further analyses.

A total of 7322 SNPs was selected for Infinium genotyping (4703 from the first set and 2619 from the second set). They were well distributed *in silico* over the Arabidopsis genome in order to obtain an even distribution of markers in the *B*. *napus* genome. The 7322 SNPs targeted 4190 EST contigs [91]. To facilitate their use in a later GoldenGate genotyping assay, we also took care to select those SNPs that were at least 60 bp from another polymorphism. For SNPs derived from ‘Tapidor’ and ‘Ningyou7’, we also only considered SNPs that were at least 60 bp from an intron.

### SNP genotyping

DNA was isolated from young leaves and DNA extracted using the DNeasy 96 Plant Kit (Qiagen, Courtaboeuf, France). DNA was quantified with the Quant-iT™ PicoGreen® Assay (Invitrogen, Carlsbad, USA), using the Appliskan multiplate reader (Thermo Scientific, Courtaboeuf, France). Concentrations were adjusted to a minimum of 50 ng/μL and were submitted to a provider, where the Infinium® assay was performed following the manufacturer's protocol (Illumina Inc., San Diego, USA). The automatic allele calling for each locus was accomplished using the Genome Studio software (Illumina Inc., San Diego, USA). The clusters were manually edited when necessary. Technical replicates and signal intensities were controlled and only the most reliable calls were retained.

### Genetic maps construction

#### Individual genetic maps

For DYDH and TNDH, PCR markers that were previously genotyped [27,28,36] were added to the genotyping SNP matrix. Segregation of each marker was tested by Chi-square test for goodness of fit (1:1; P = 0.01) for the DYDH, AADH, AMDH and TNDH populations. At first, Mapmaker Exp/3.0b [93] was used to build a framework map for each individual genetic map. A minimum LOD score of 4.0 with a maximum genetic distance of 30 cM was first used to associate loci into initial linkage groups. A full multipoint linkage analysis was performed to determine the most probable locus order of highly informative markers (order with a LOD of 3.0 and with the highest log-likelihood ratio) for each linkage group. The remaining markers from each linkage group were manually integrated at their most likely position using the ‘try’ command. Double-crossover events were examined and the original scores rechecked for potential scoring errors. The order of the final set of framework loci within the linkage groups was re-verified using the ‘ripple’ command with a sliding window of five loci and a LOD score threshold of 3.0. Once the framework maps were built, the remaining markers were mapped using the ActionMap software [94]. Each locus was mapped independently to the framework map, so low-quality mapping of some loci did not alter subsequent mapping of other loci and results referred to a stable reference map. All genetic distances were expressed in centimorgans using the Kosambi mapping function [95]. Linkage groups were named according to the international Brassica nomenclature with A1- A10 and C1- C9 corresponding to the linkage groups of the A and C genomes, respectively. They were oriented as in Parkin et al. [22].

#### Integrated genetic map

Once the individual genetic maps were obtained, an integrated map was constructed through a projection process using the BioMercator V3.2.2 software [96]. Using the iterative projection method, and in order to limit error propagation, the projection process started with the map that presents the more stable framework and well ordered and spread loci *i*.*e*. the map derived from the DYDH population.

### Selection of a 1536 SNP set and validation in a GoldenGate assay

A set of 1536 SNPs were further chosen from the previous Infinium® assay to design four custom VeraCode OPA sets for the Illumina BeadXpress Reader. They were evenly distributed on the integrated map and showed a low number of missing data (<5%) and high minor allele frequencies (MAF > 10%) on the OSR panel. For each OPA run, a plate of 96 samples with 5 μL of genomic DNA normalized to 50 ng/μL was genotyped using the “GoldenGate Genotyping Assay for VeraCode Manual Protocol” (Illumina Inc., San Diego, USA). The 96 samples were a subset of the collection previously used for Infinium genotyping. Automatic allele calling for each locus was accomplished using the Genome Studio software (Illumina Inc., San Diego, USA). The clusters were manually edited when necessary.

### Homology search with arabidopsis and *B*. *Rapa*

The sequences associated with each set of genetic markers were used as queries in homology searches against the Arabidopsis thaliana pseudo-chromosomes (TAIR10 release, ftp://ftp.arabidopsis.org/home/tair/Sequences/blast_datasets/TAIR10_blastsets/, version date: 16/04/2012), and against *B*. *rapa* Chiifu-401 pseudo-chromosomes in EnsemblPlants (IVF-CAASv1.14, ftp://ftp.ensemblgenomes.org/pub/plants/release-14/fasta/Brassica_rapa/dna/, version date: 27/05/2012). For homology searches on *Arabidopsis thaliana*, following parameters were used: TBlastX with match = 1, mismatch = −3, gap open penalty = −1, gap extension penalty = −1, word size = 3, and low complexity sequences filtered. A fairly low expect value (E-value) was used as the exclusion cutoff (1E-06). At least five consecutive homologous loci were required to define a collinearity block. Collinearity blocks were colour- coded according to the convention of Schranz *et al*. [46]. For homology searches on *B*. *rapa* pseudo-chromosomes, following parameters were used: BlastN with match = 1, mismatch = −3, gap open penalty = 1, gap extension penalty = 2, word size = 7, and low complexity sequences filtered. A fairly low expect value (E-value) was used as the exclusion cutoff (1E-06).

### Statistical analyses

The minor allele frequencies (MAF), percentage of heterozygosity and polymorphism information content (PIC) were estimated for each SNP marker within the different collections using PowerMarker v3.25 software [97]. Mean PIC values were compared with Wilcoxon test (α = 5%). Population differentiation was studied using analysis of molecular variance (AMOVA) performed with Arlequin v3.1 software [98] and principal component analyses (PCA) performed with Darwin v5.0.158 [99]. LD was estimated as the correlation coefficient r^2^ between all pairs of SNPs (with MAF > 5%) within and between linkage groups using PLINK v1.07 program [100]. The overall decay of LD in relation to genetic distance was evaluated with R software (R development Core team, 2011) using the non linear regression of r^2^ according to Gaut and Long [101] with E[r^2^] = 1/(1 + 4N_e_c) where c is the recombination rate in Morgans and N_e_ the effective population size. LD heatmaps were built for each linkage group with the R package LDheatmap implemented in R software [102].

## Competing interests

The authors declare no competing interests.

## Authors’ contributions

RD contributed to the design of the study, performed genetic diversity and linkage disequilibrium analyses and wrote the manuscript. CF carried out genetic mapping and BLAST analyses. BFF contributed to the genetic diversity and linkage disequilibrium studies. GL contributed to genetic mapping and BLAST analyses. JPP, JD, IA and NR selected the SNPs after sequence capture studies. NR carried out genetic mapping. CF, VG, NL, AM, MP, GT acquired the genotyping data. MB and JPM organized the production of genotyping data. PB contributed to the design of the study. JPM coordinated the project. JP helped analyze the results and participated in the coordination of the study. All authors read and approved the final manuscript.

## Supplementary Material

Additional file 1: Table S1 Description of the individual genetic maps obtained on the ‘Darmor-bzh’ x ‘Yudal’ (DYDH), ‘Tapidor’ x Ningyou7’ (TNDH), ‘Aviso’ x ‘Abumasari’ (AADH) and ‘Aviso’ x ‘Montego’ (AMDH) DH populations. Total number of markers and SNPs per LG and on the A, C and whole genomes and ratio of marker and SNP number on the A genome relatively to the C genome (A/C) are indicated. Click here for file

Additional file 2: Table S2 Summary of data obtained for all the markers: assignment to linkage groups, collinearity blocks, centromere, erucic genes and glucosinolate QTL positions, position on the integrated and the individual maps, segregation distortion and PIC values on the different collections. Click here for file

Additional file 3: Figure S1 Alignment of the four individual maps obtained for the TNDH, DYDH, AADH and AMDH populations and the integrated map (on the right). The blocks as defined by Schranz et al. [46] based on their collinearity with *A*. *thaliana* are indicated with the capital letters A to X on the left of each linkage group. Click here for file

Additional file 4: Figure S2
Dot-plots obtained for each linkage group between the integrated map and all four individual TNDH, DYDH, AADH and AMDH maps. The marker order on the vertical axis is from the four individual maps and the marker order on the horizontal axis is from the integrated map. Cumulative genetic distance in cM is indicated on each axis. (PDF 471 kb) Click here for file

Additional file 5: Table S3 List of the 222 SNPs that were assigned to different linkage groups (LG) according to the mapping populations. Click here for file

Additional file 6: Table S4 List and characteristics of the 1536 public SNPs : Position on the integrated map (or on the individual maps for multi-loci SNPs), Context sequence and SNP position, Arabidopsis and B. rapa hits. Click here for file

Additional file 7: Figure S3 Distribution of PIC values along the linkage groups in the fodder rape (FO), spring (SOSR) and winter (WOSR) oilseed rape and the different seed quality subgroups (“++”,” 0+”,” 00”) within WOSR. PIC was averaged across a sliding window of 10 cM with a step of one cM. Click here for file

Additional file 8: Figure S4 Plots of r^2^ as a function of genetic distance (in cM) between pairs of SNPs on each linkage group in the whole, spring, winter and “00” winter oilseed rape collections. Red curves show the non-linear regressions trend line of r^2^ versus genetic distance. Click here for file

Additional file 9: Figure S5 Linkage disequilibrium heatmaps for each linkage group in the whole, spring (SOSR), winter (WOSR) and “00” WOSR collections. The putative position of the centromeres is indicated by a black arrow. Click here for file

Additional file 10: Table S5 List of accessions used for the diversity analysis and LD study. Click here for file
