# Supplementary material for: High-density SNP-based genetic map development and linkage disequilibrium assessment in Brassica napus L
Source: BMC Genomics. 2013 Feb 22;14:120. doi: 10.1186/1471-2164-14-120 (PMC3600037; doi:10.1186/1471-2164-14-120)
Supplement: Additional file 3: Figure S1 — Alignment of the four individual maps obtained for the TNDH, DYDH, AADH and AMDH populations and the integrated map (on the right). The blocks as defined by Schranz et al. [46] based on their collinearity with A. thaliana are indicated with the capital letters A to X on the left of each linkage group. [file 1471-2164-14-120-S3.pdf]

Supplementary Figure 1: Alignment of the four individual maps obtained for the TNDH, DYDH, AADH and AMDH populations and the integrated map (on the right). The blocks as defined by Schranz et al (2006) based on their collinearity with *A. thaliana* are indicated with the capital letters A to X on the left of each linkage groups.

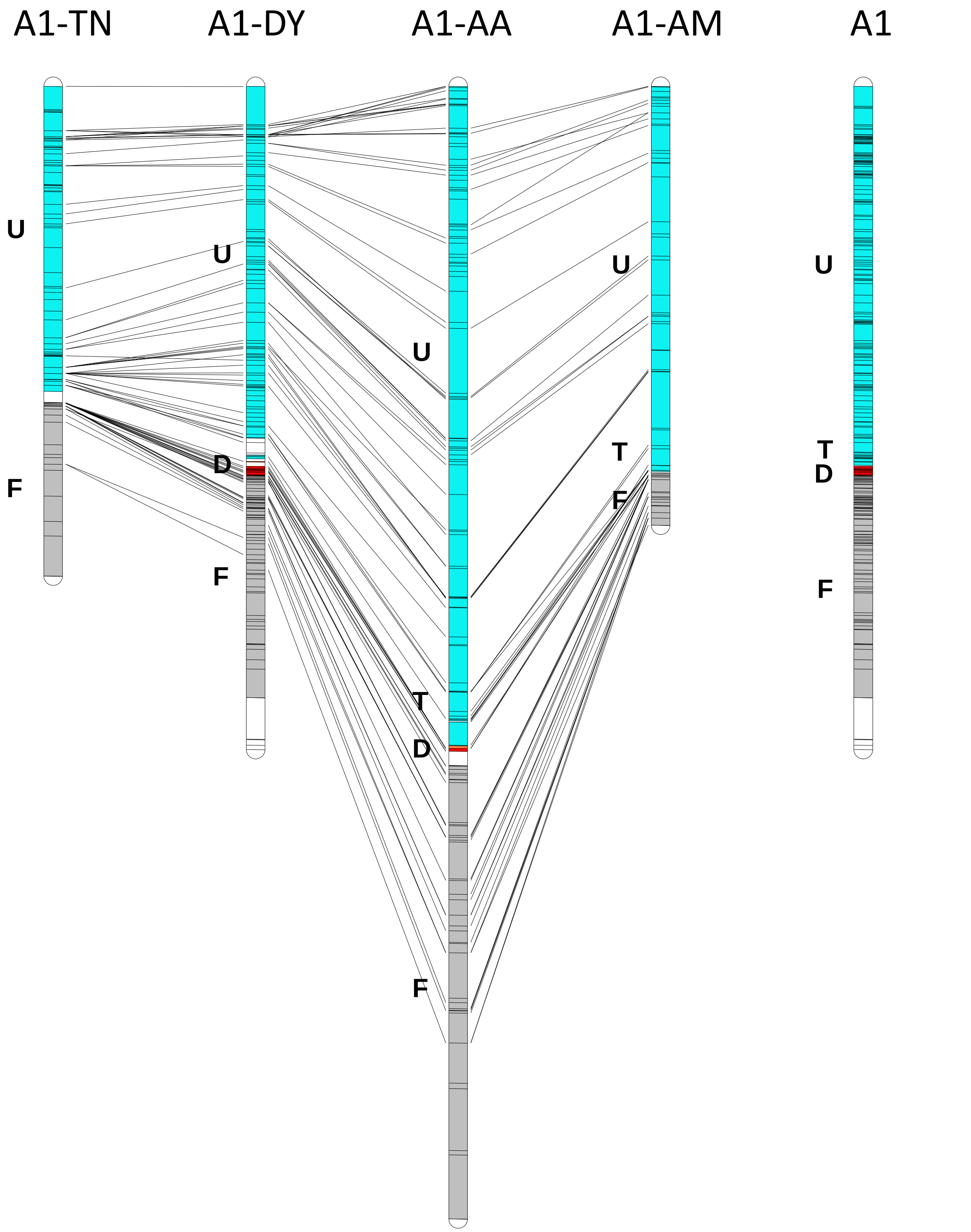

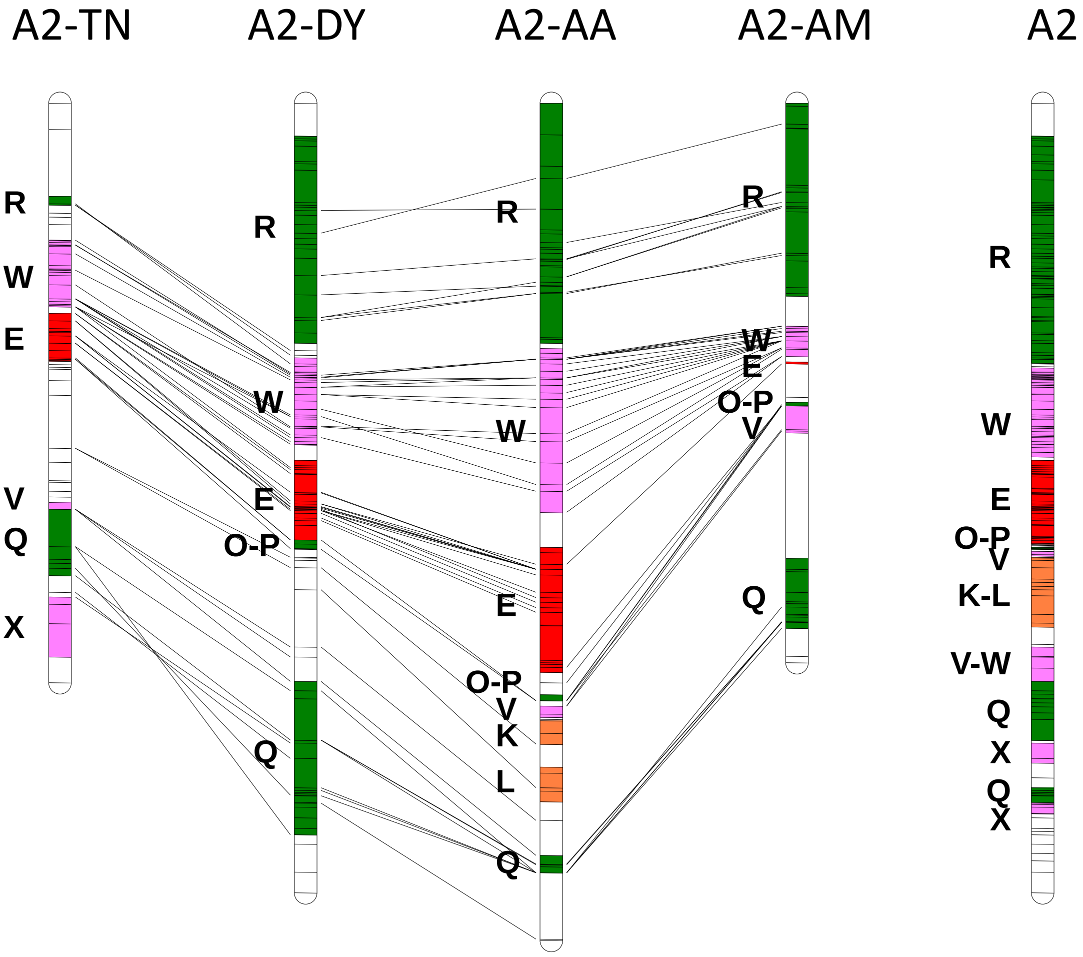
A2-TN
A2-DY
A2-AA
A2-AM
A2
R
W
E
V
Q
X
R
W
E
O-P
Q
R
W
E
O-P
V
K
L
Q
R
W
E
O-P
V
Q
R
W
E
O-P
V
K-L
V-W
Q
X
Q
X

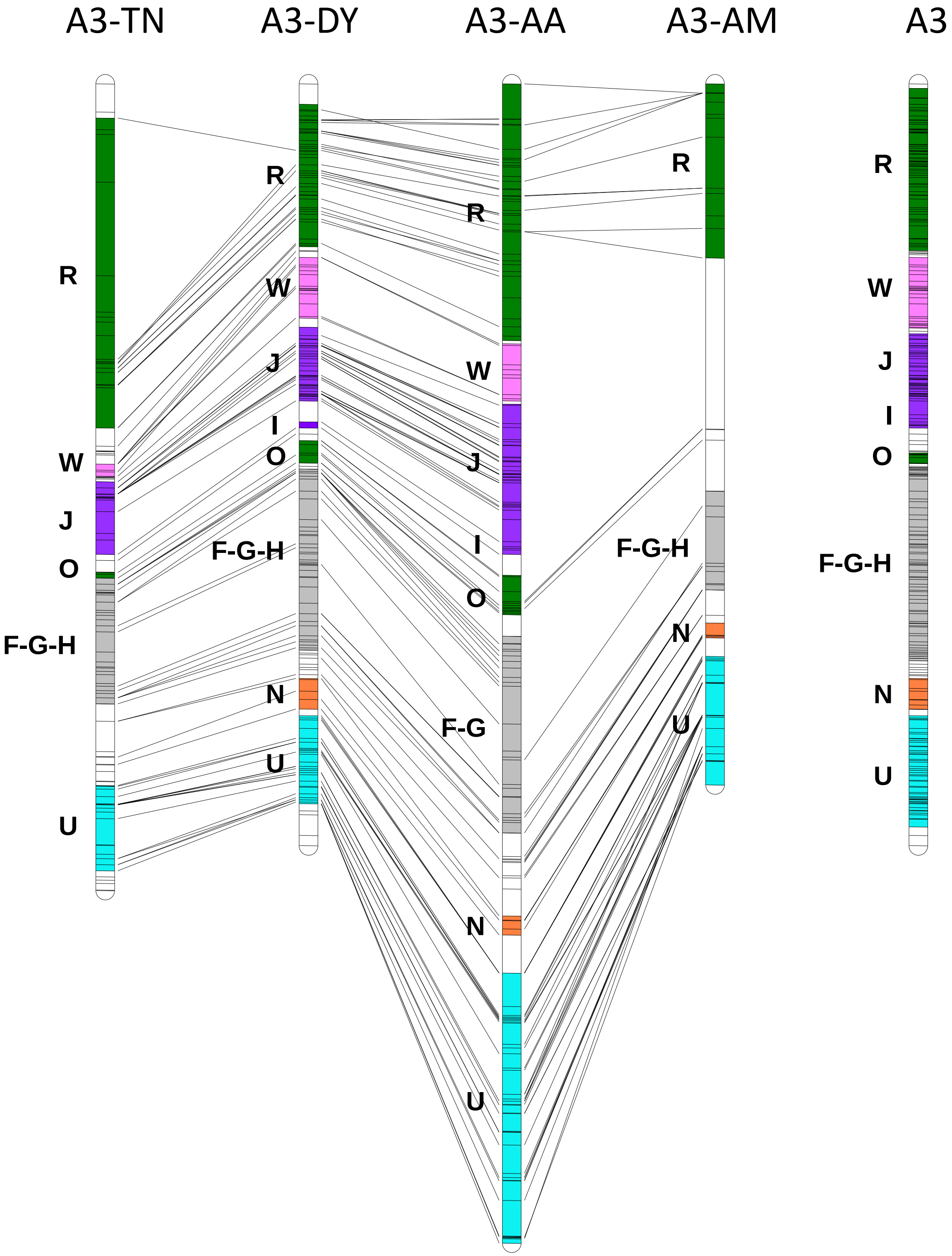
A3-TN
A3-DY
A3-AA
A3-AM
A3
R
W
J
O
F-G-H
U
R
W
J
I
O
F-G-H
N
U
R
W
J
I
O
F-G
N
U
R
F-G-H
N
U
R
W
J
I
O
F-G-H
N
U

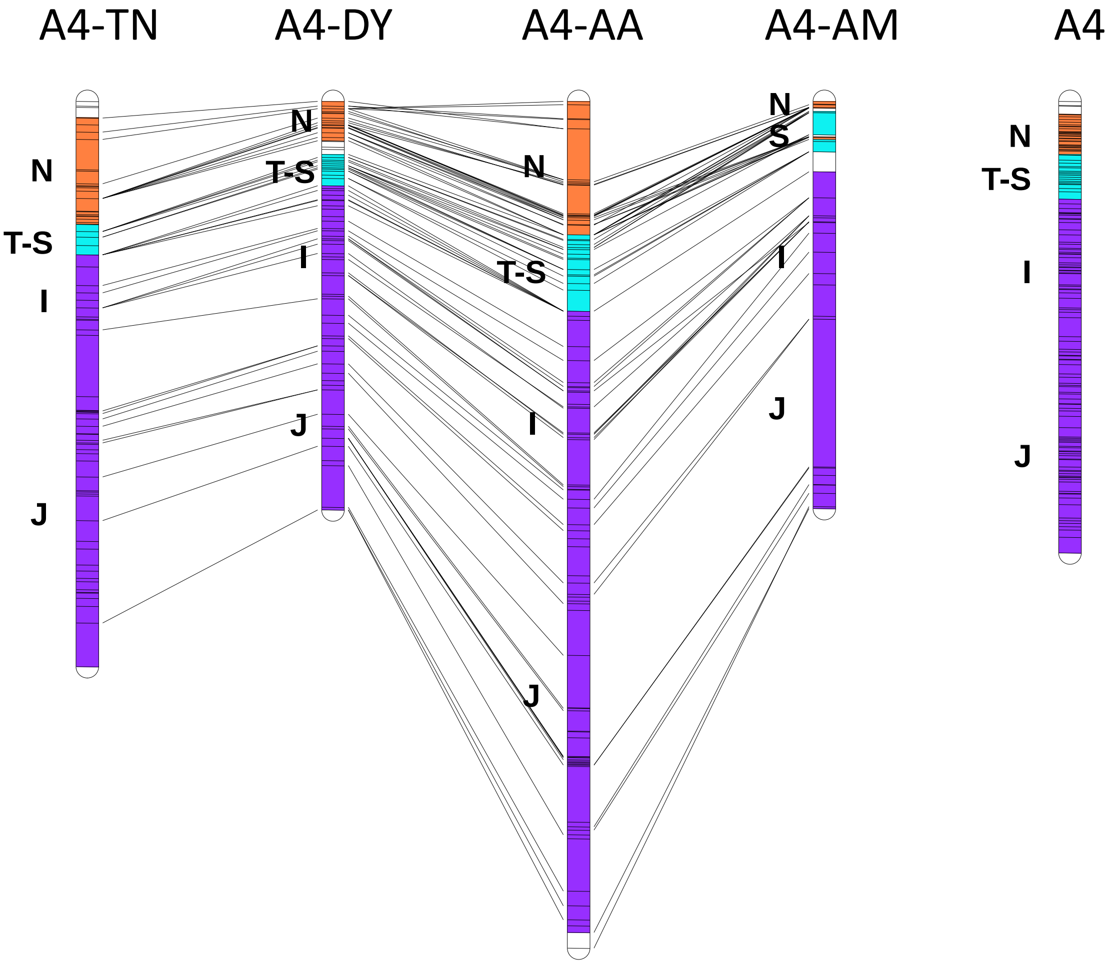

A4-TN
A4-DY
A4-AA
A4-AM
A4
N
T-S
I
J
N
T-S
I
J
N
T-S
I
J
N
S
I
J
N
T-S
I
J

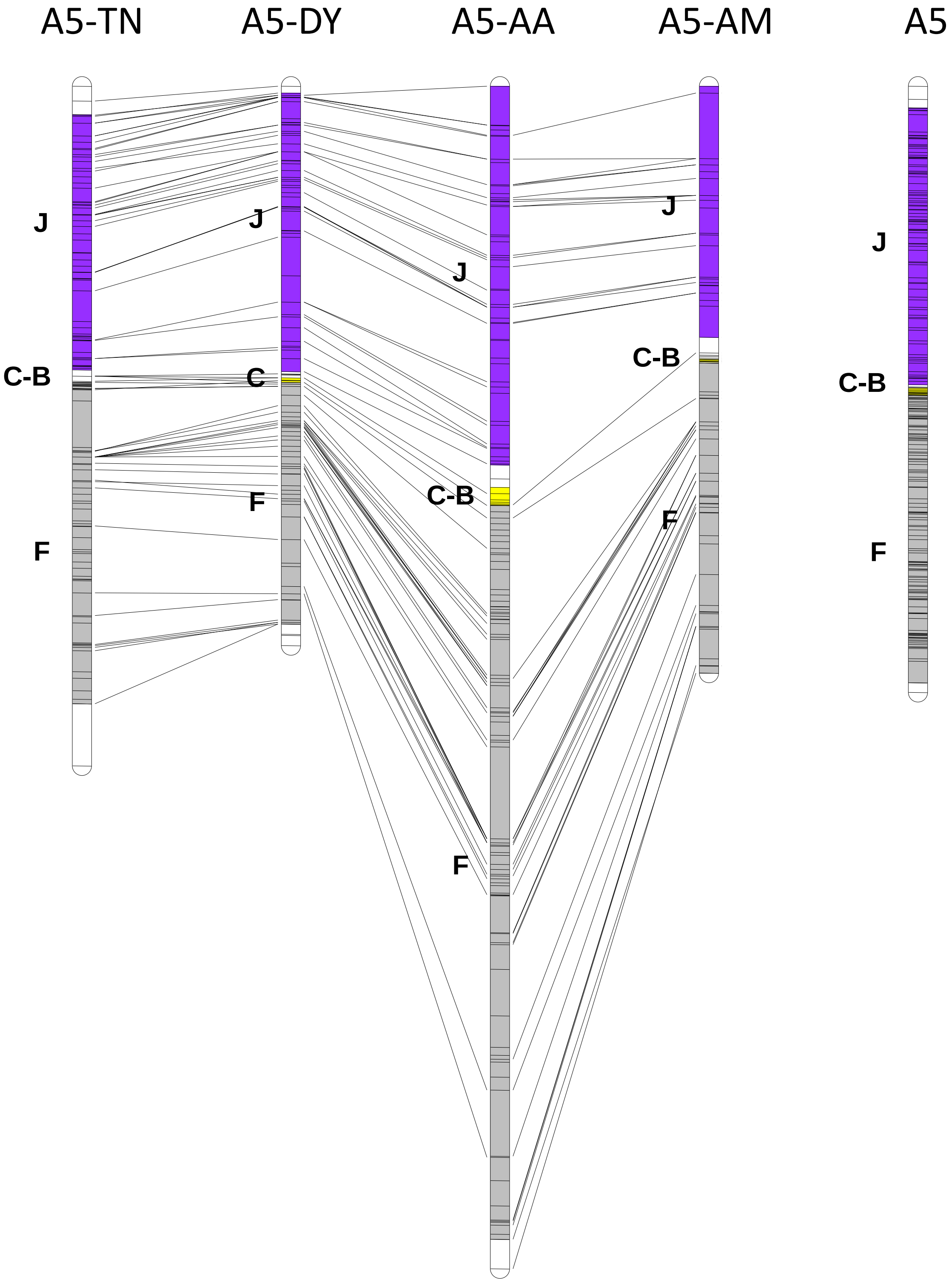

A5-TN
A5-DY
A5-AA
A5-AM
A5
J
C-B
F
J
C
F
J
C-B
F
J
C-B
F
J
C-B
F

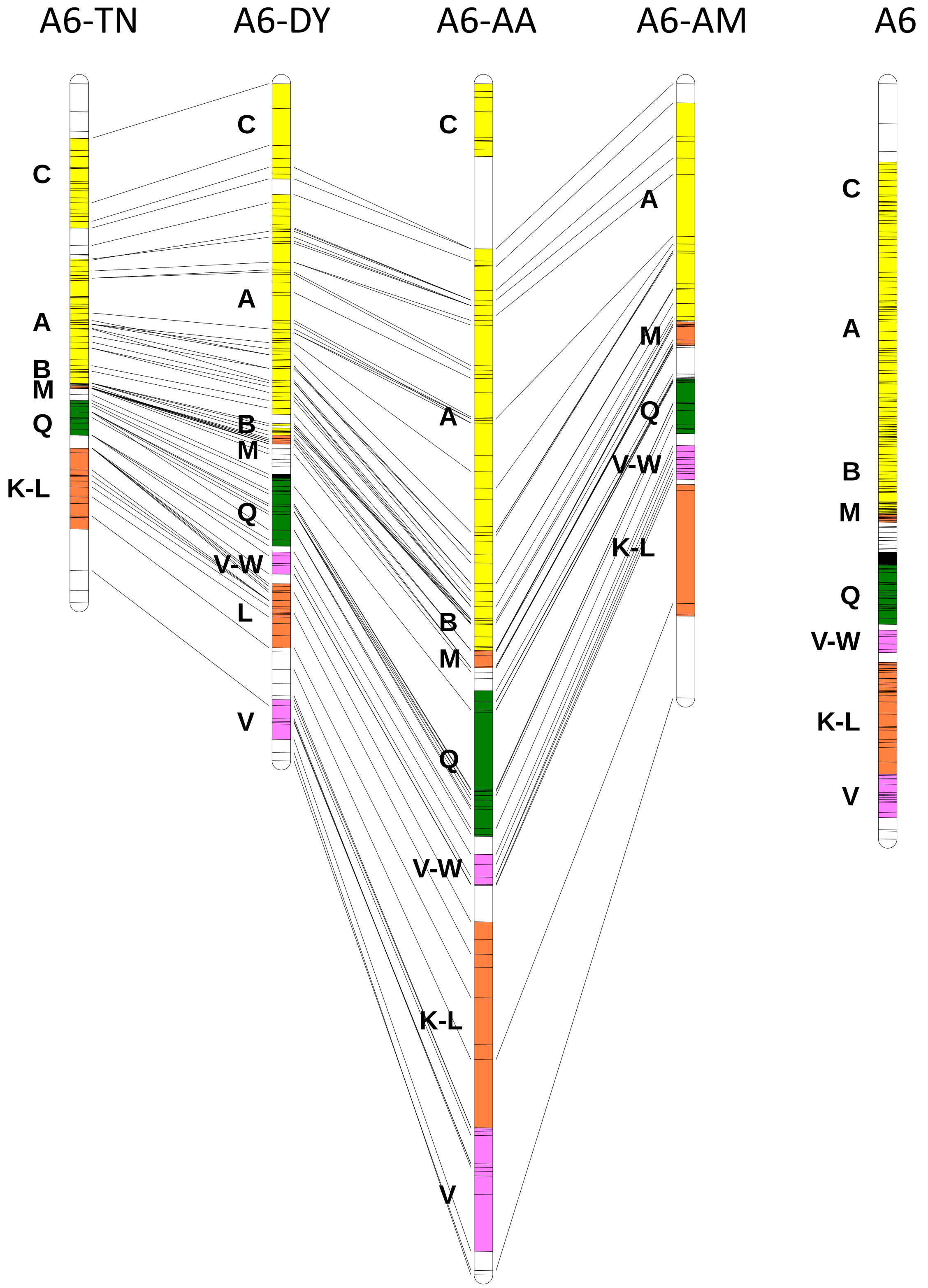

A6-TN
A6-DY
A6-AA
A6-AM
A6
C
A
B
M
Q
K-L
V-W
L
V

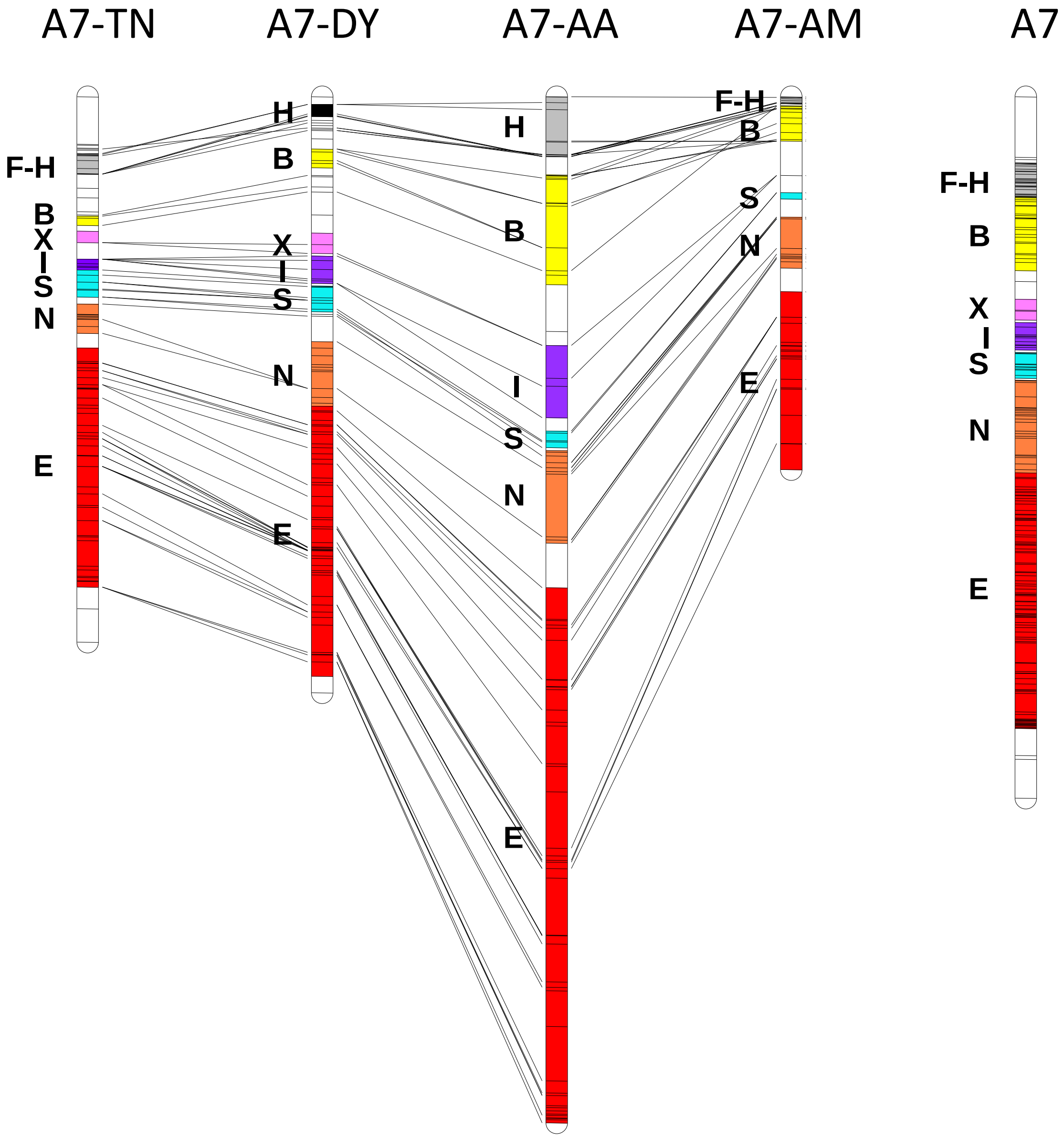
A7-TN
A7-DY
A7-AA
A7-AM
A7
F-H
B
X
I
S
N
E
H
B
X
I
S
N
E
H
B
I
S
N
E
F-H
B
S
N
E
F-H
B
X
I
S
N
E

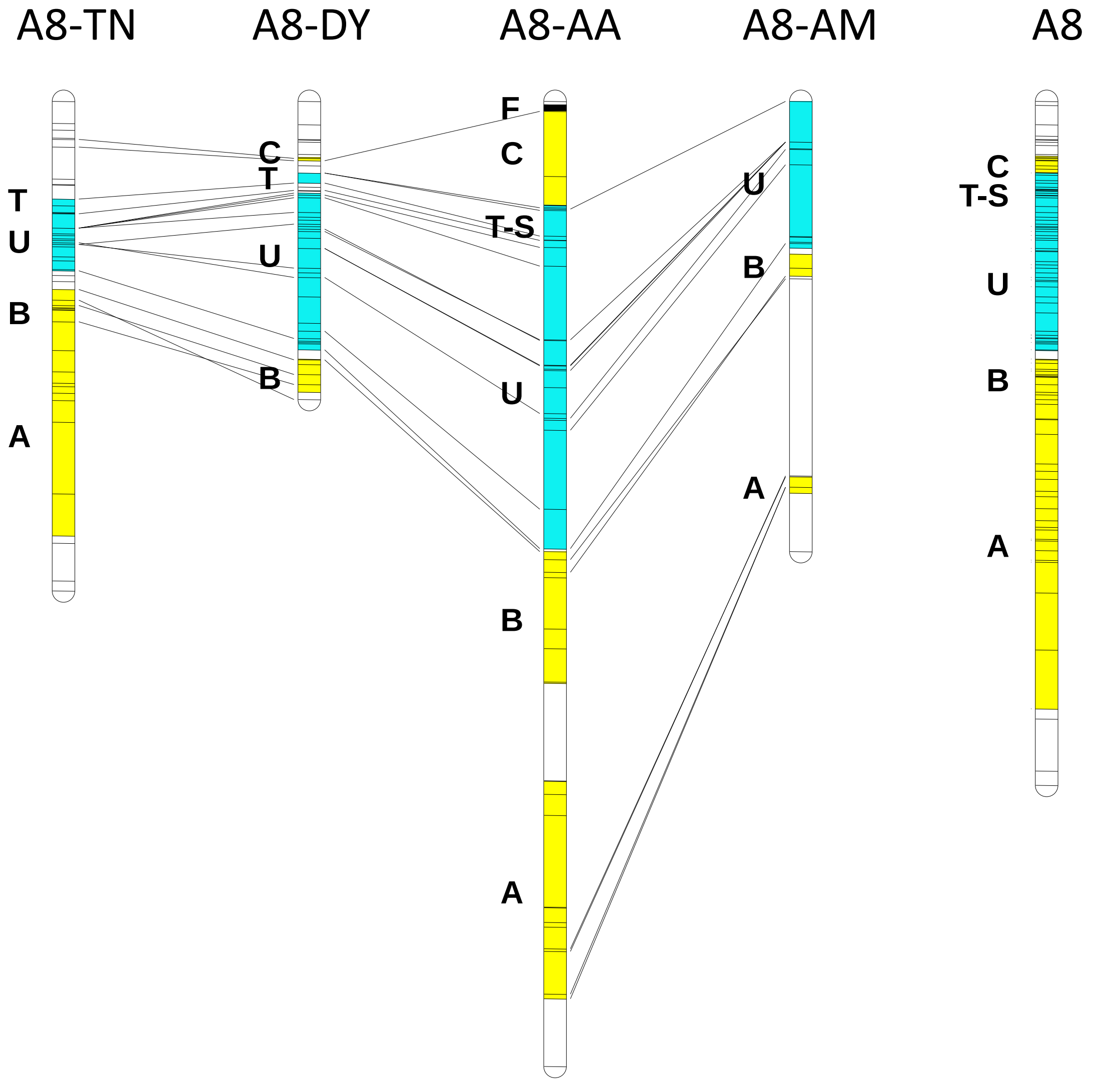

A8-TN
A8-DY
A8-AA
A8-AM
A8
T
U
B
A
C
T
U
B
F
C
T-S
U
B
A
U
B
A
C
T-S
U
B
A

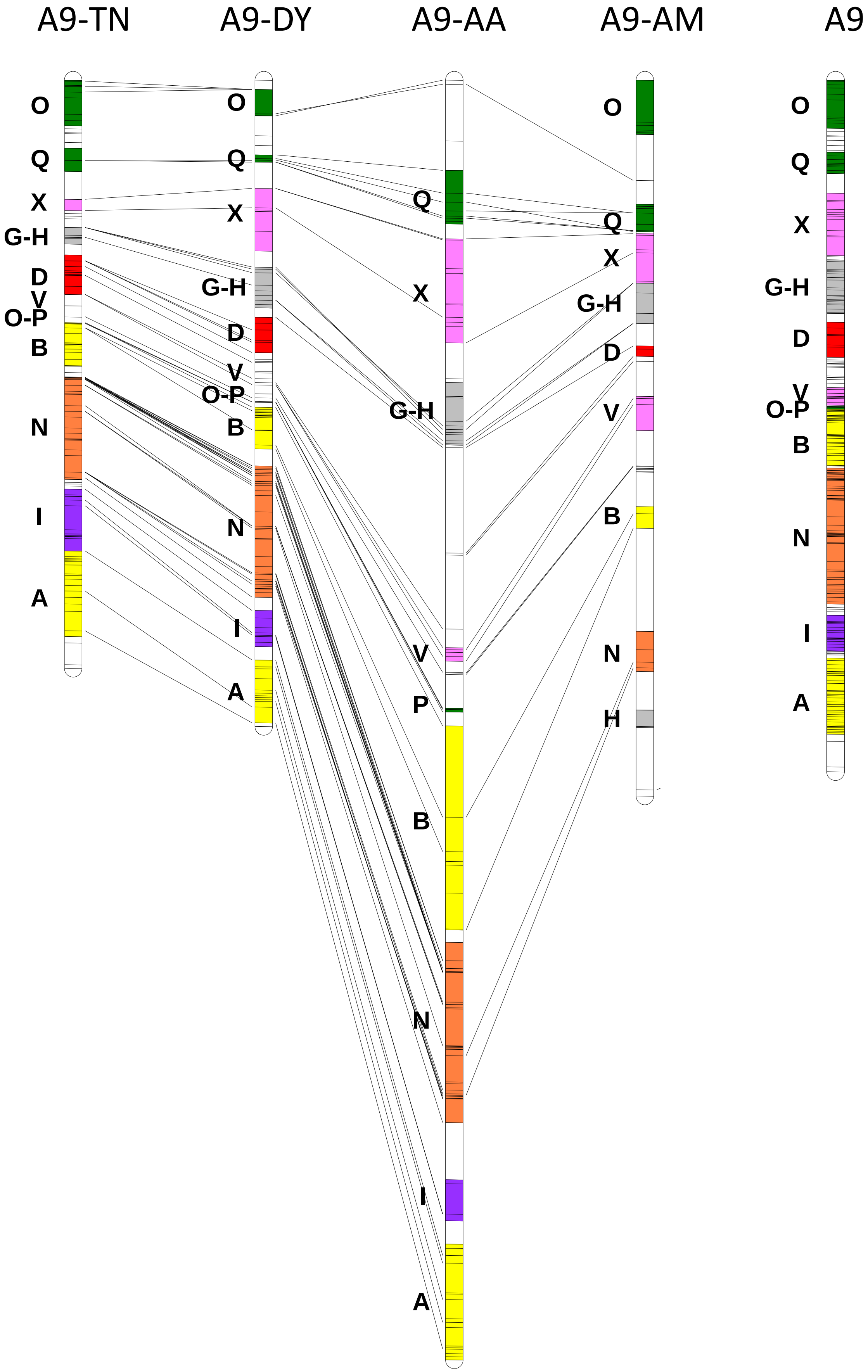
A9-TN
A9-DY
A9-AA
A9-AM
A9
O
Q
X
G-H
D
V
O-P
B
N
I
A
P
H

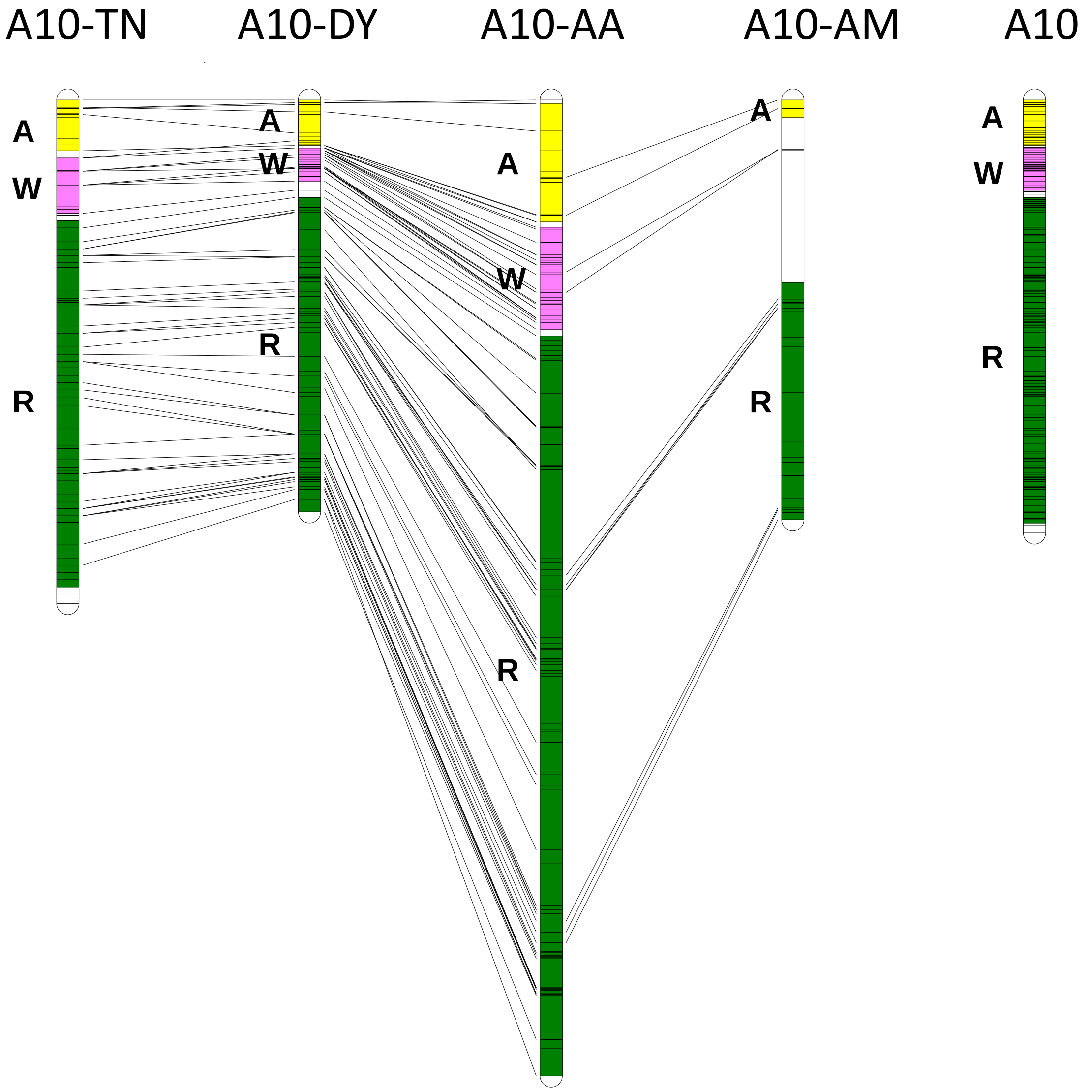
A10-TN
A10-DY
A10-AA
A10-AM
A10
A
W
R
A
W
R
A
W
R
A
R
A
W
R

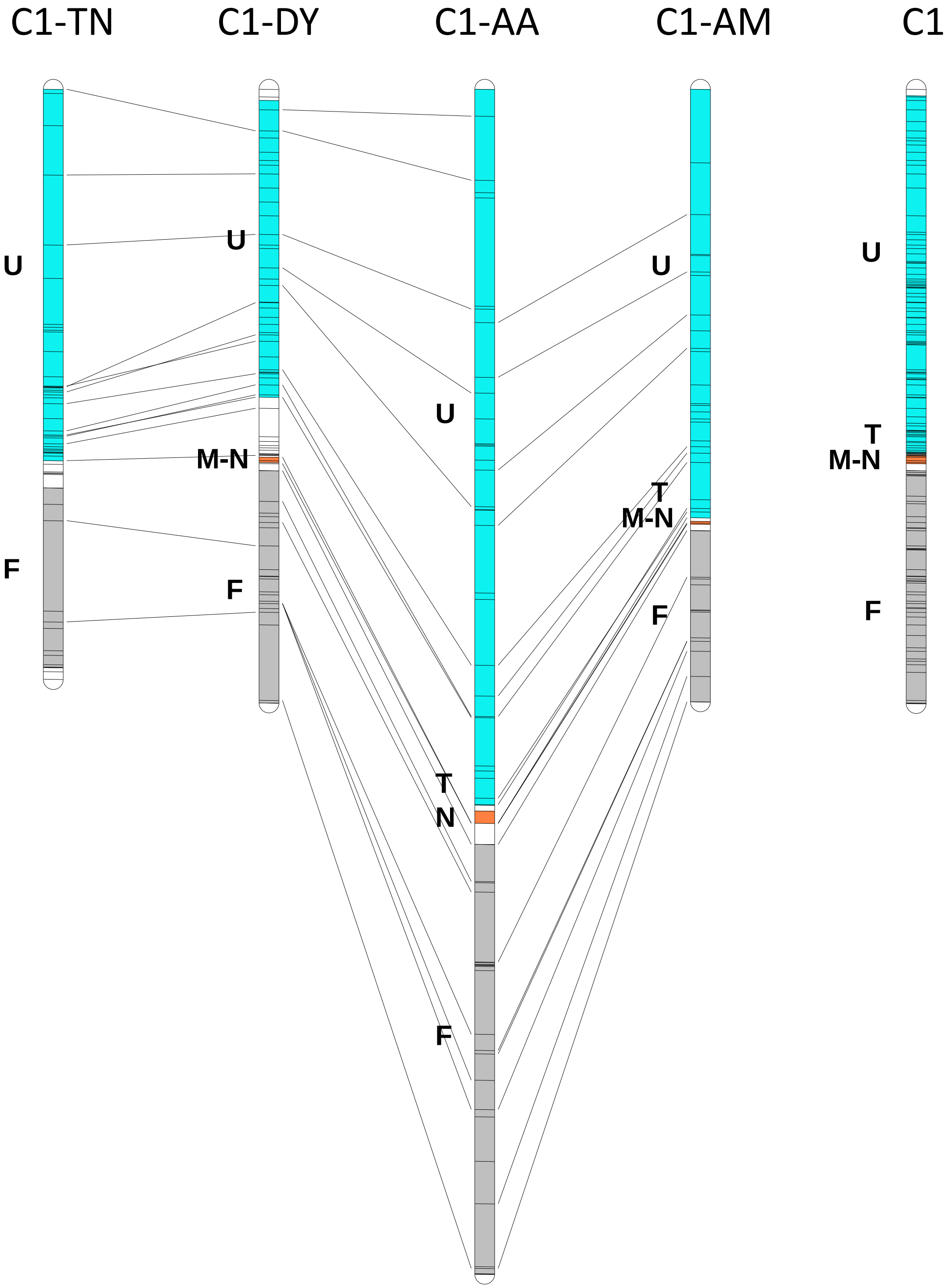
C1-TN
C1-DY
C1-AA
C1-AM
C1
U
U
U
U
U
M-N
T
N
T
M-N
T
M-N
F
F
F
F
F

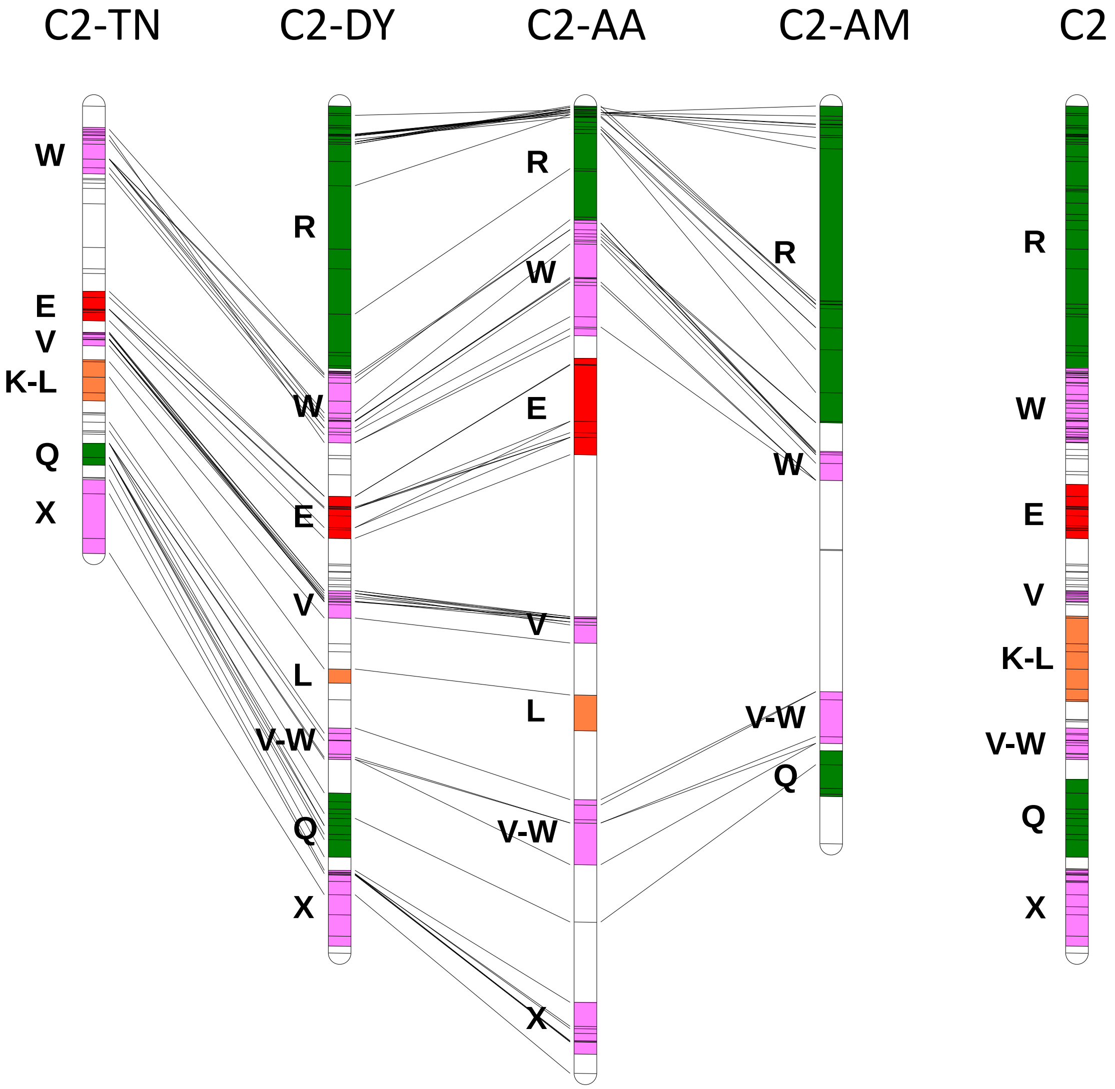
C2-TN
C2-DY
C2-AA
C2-AM
C2
W
E
V
K-L
Q
X
R
W
E
V
L
V-W
Q
X
R
W
E
V
L
V-W
X
R
W
V-W
Q
R
W
E
V
K-L
V-W
Q
X

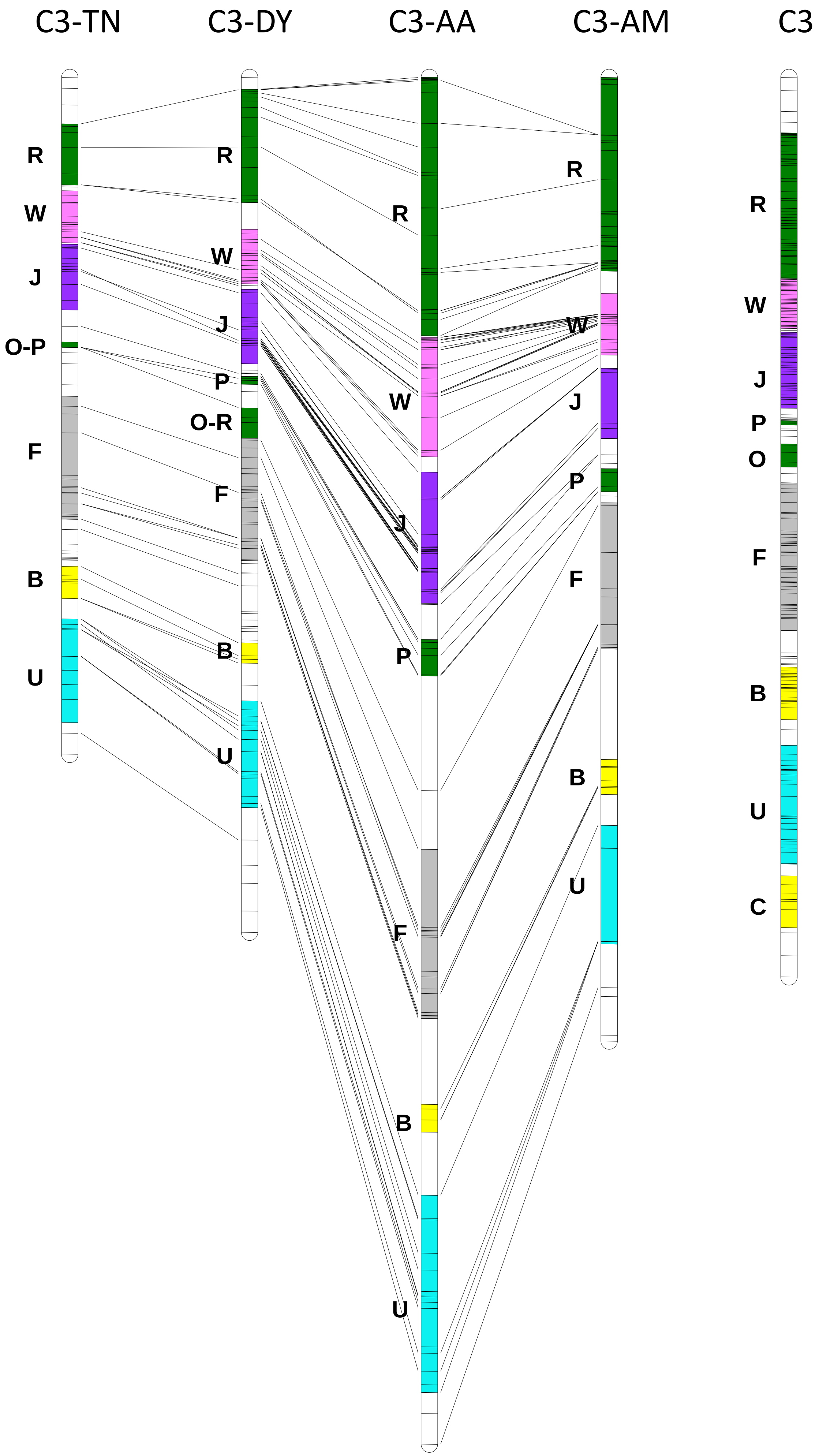
C3-TN
C3-DY
C3-AA
C3-AM
C3
R
W
J
O-P
F
B
U
R
W
J
P
O-R
F
B
U
R
W
J
P
F
B
U
R
W
J
P
F
B
U
R
W
J
P
O
F
B
U
C

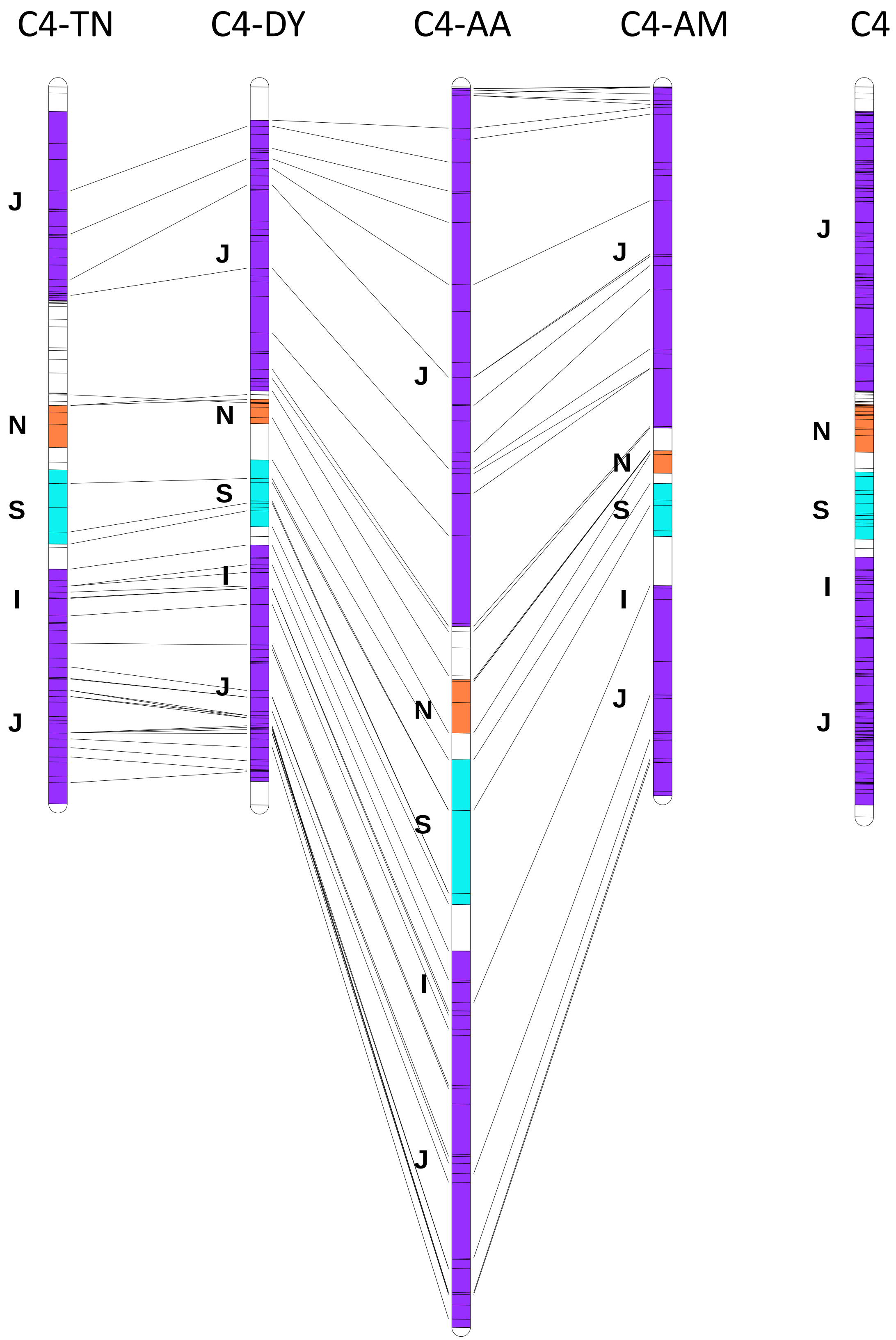
C4-TN
C4-DY
C4-AA
C4-AM
C4
J
N
S
I
J

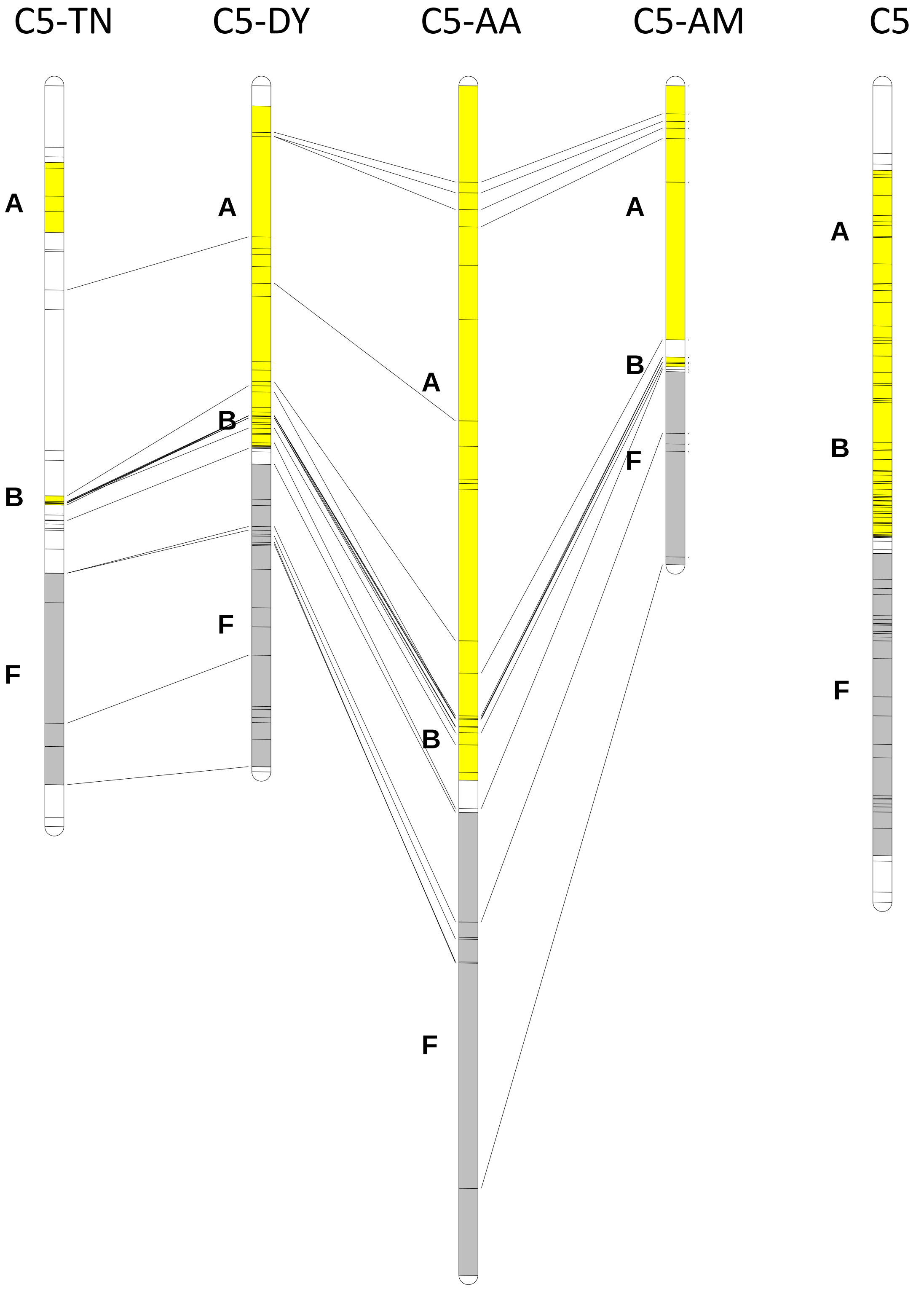
C5-TN
C5-DY
C5-AA
C5-AM
C5
A
B
F
A
B
F
A
B
F
A
B
F
A
B
F

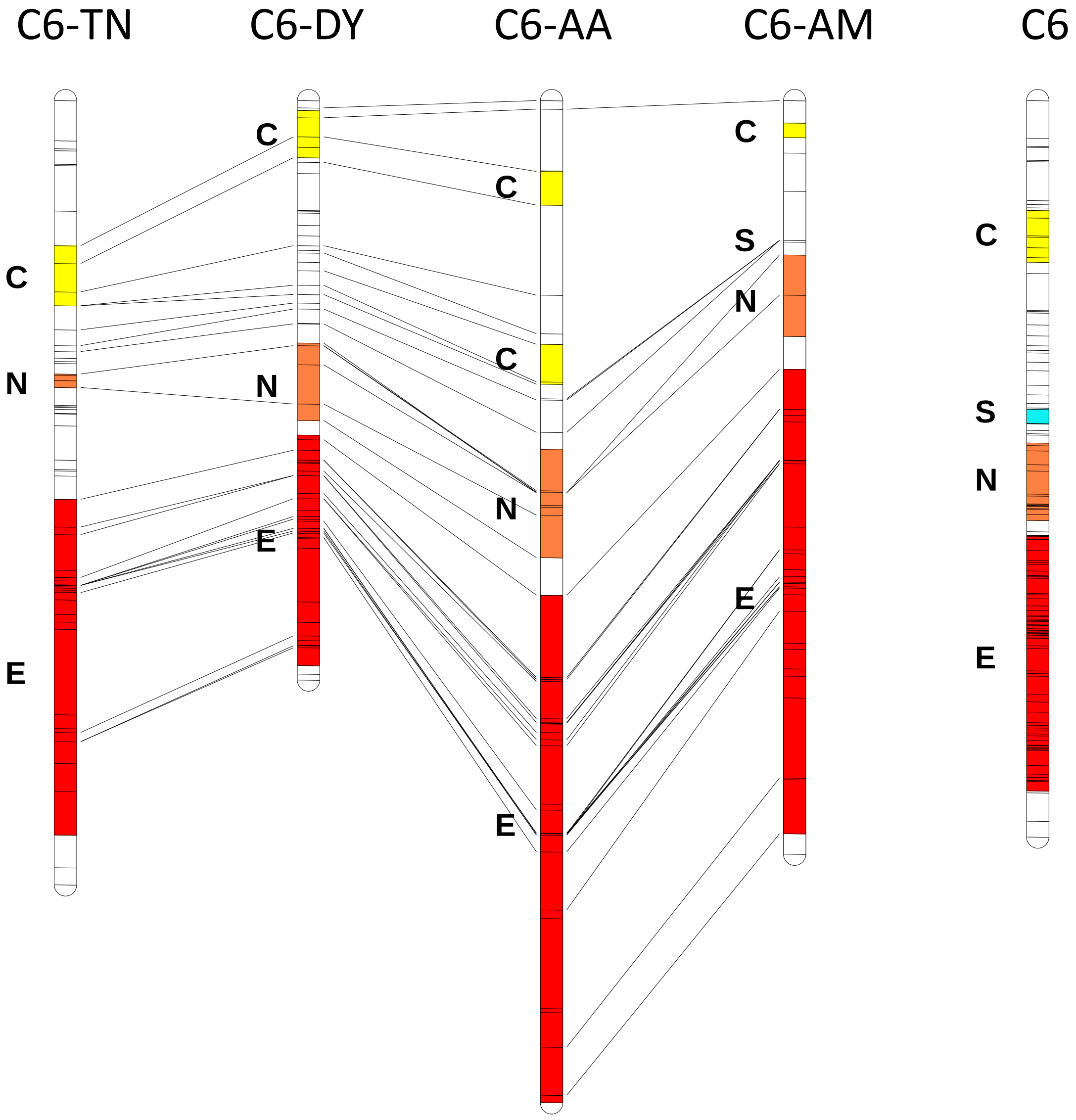

C6-TN
C6-DY
C6-AA
C6-AM
C6
C
N
E
C
N
E
C
C
N
E
C
S
N
E
C
S
N
E

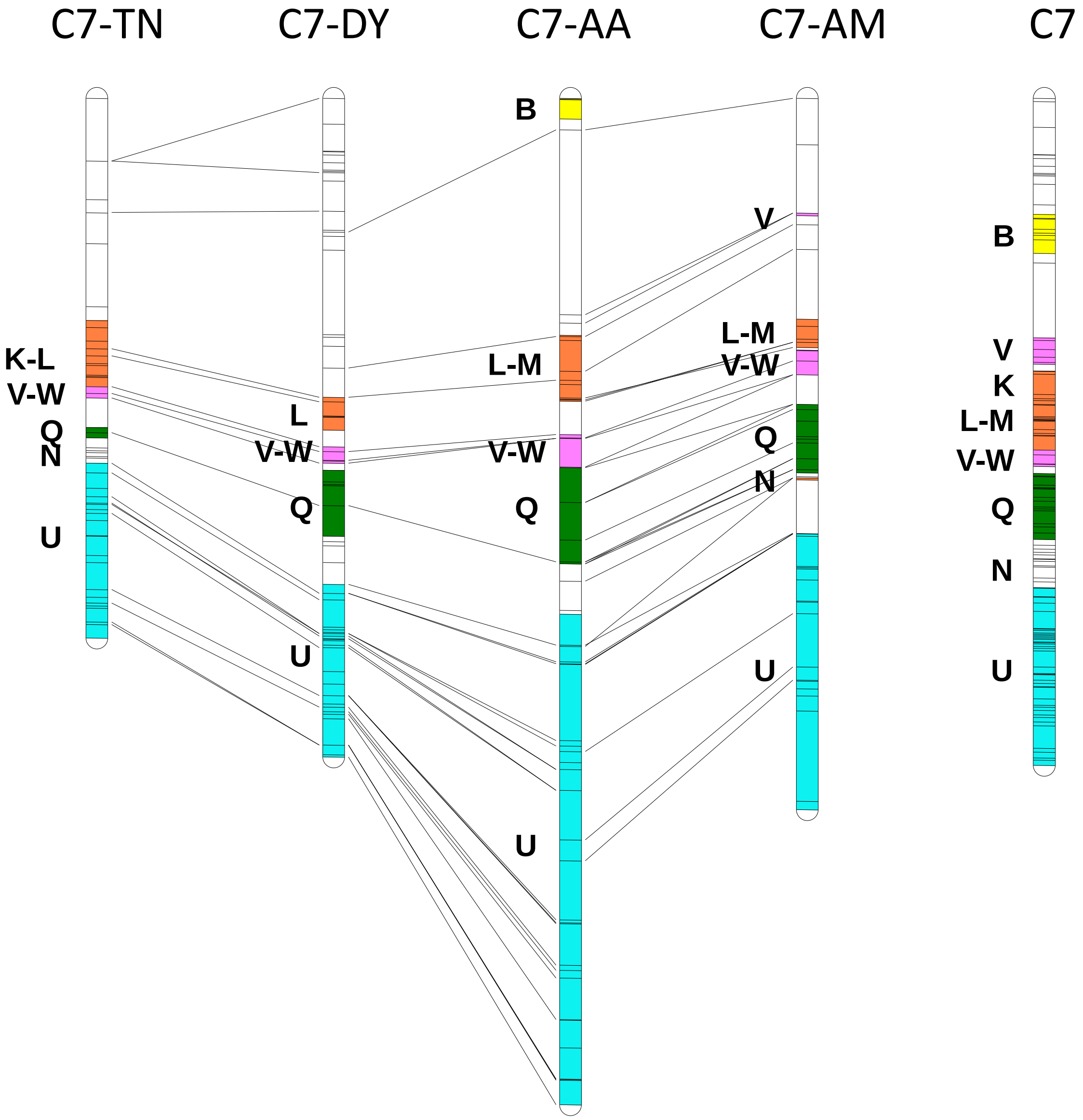
C7-TN
C7-DY
C7-AA
C7-AM
C7
K-L
V-W
Q
N
U
L
V-W
Q
U
B
L-M
V-W
Q
U
V
L-M
V-W
Q
N
U
B
V
K
L-M
V-W
Q
N
U

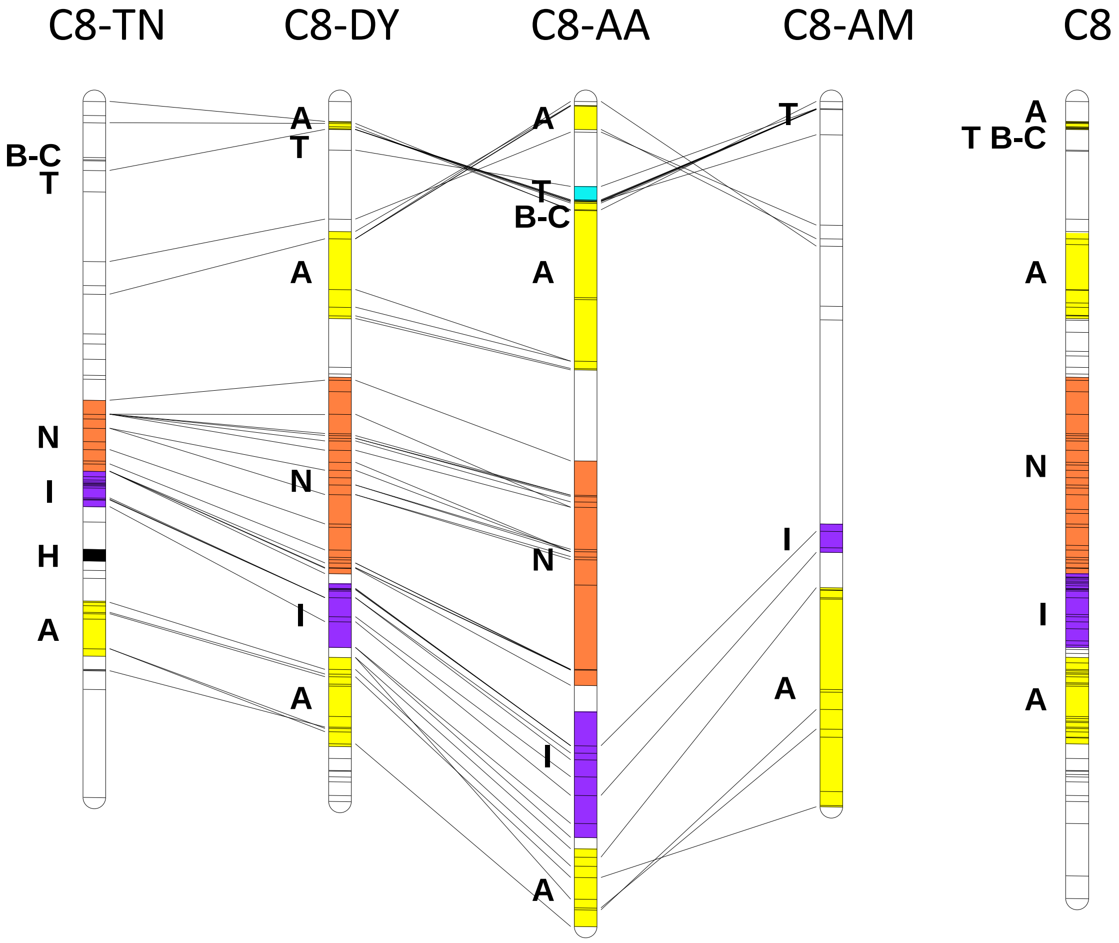

C8-TN
C8-DY
C8-AA
C8-AM
C8
B-C
T
N
I
H
A
A
T
A
N
I
A
A
T
B-C
A
N
I
A
T
I
A
A
T B-C
A
N
I
A

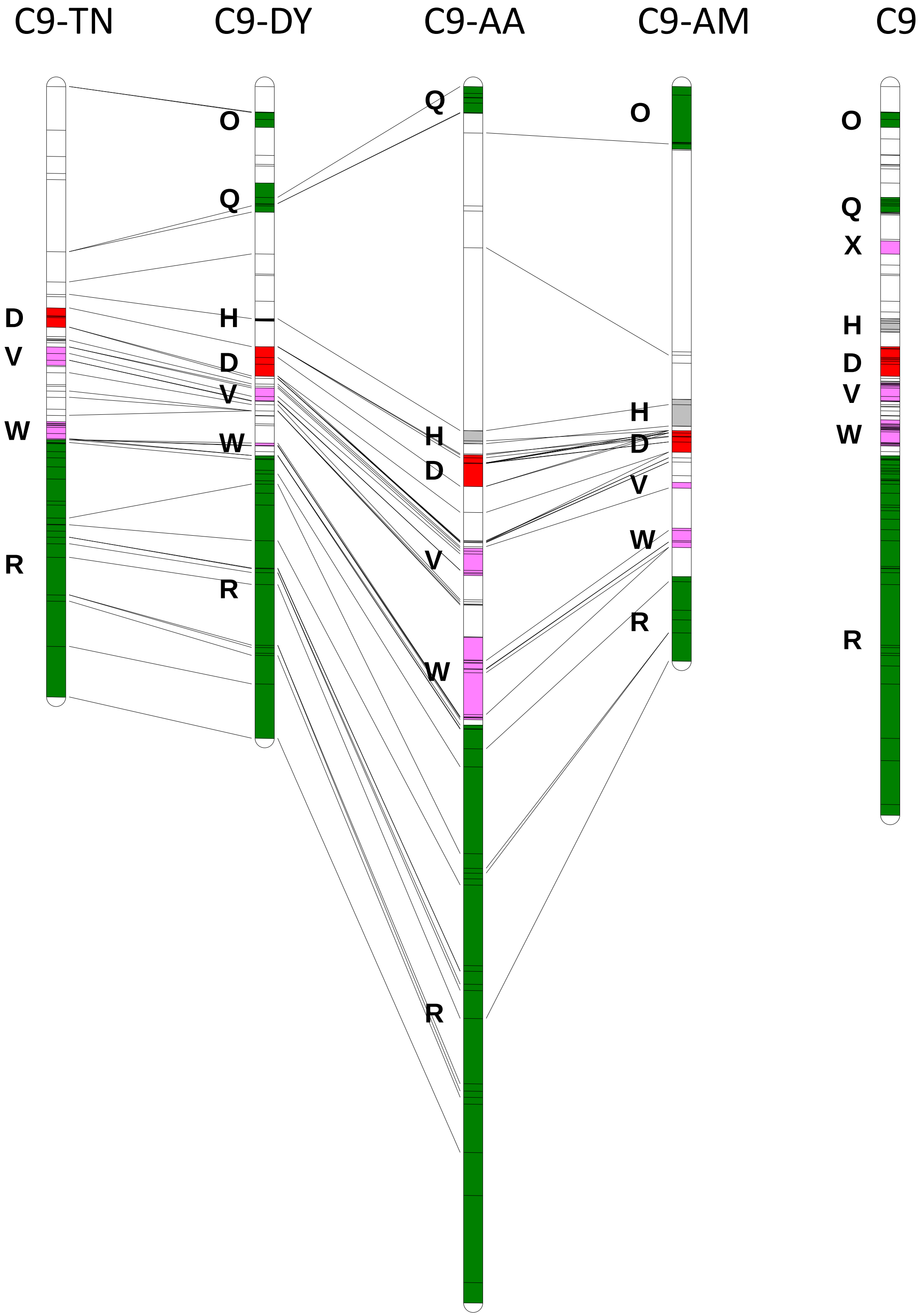
C9-TN
C9-DY
C9-AA
C9-AM
C9
O
Q
X
H
D
V
W
R
