# Supplementary material for: High-density SNP-based genetic map development and linkage disequilibrium assessment in Brassica napus L
Source: BMC Genomics. 2013 Feb 22;14:120. doi: 10.1186/1471-2164-14-120 (PMC3600037; doi:10.1186/1471-2164-14-120)
Supplement: Additional file 4: Figure S2 — Dot-plots obtained for each linkage group between the integrated map and all four individual TNDH, DYDH, AADH and AMDH maps. The marker order on the vertical axis is from the four individual maps and the marker order on the horizontal axis is from the integrated map. Cumulative genetic distance in cM is indicated on each axis. (PDF 471 kb) [file 1471-2164-14-120-S4.pdf]

Supplementary figure 2: Dot-plots obtained for each linkage group between the integrated map and all four individual TNDH, DYDH, AADH and AMDH maps. The marker order on the vertical axis is from the four individual maps and the marker order on the horizontal axis is from the integrated map. Cumulative genetic distance in cM is indicated on each axis.

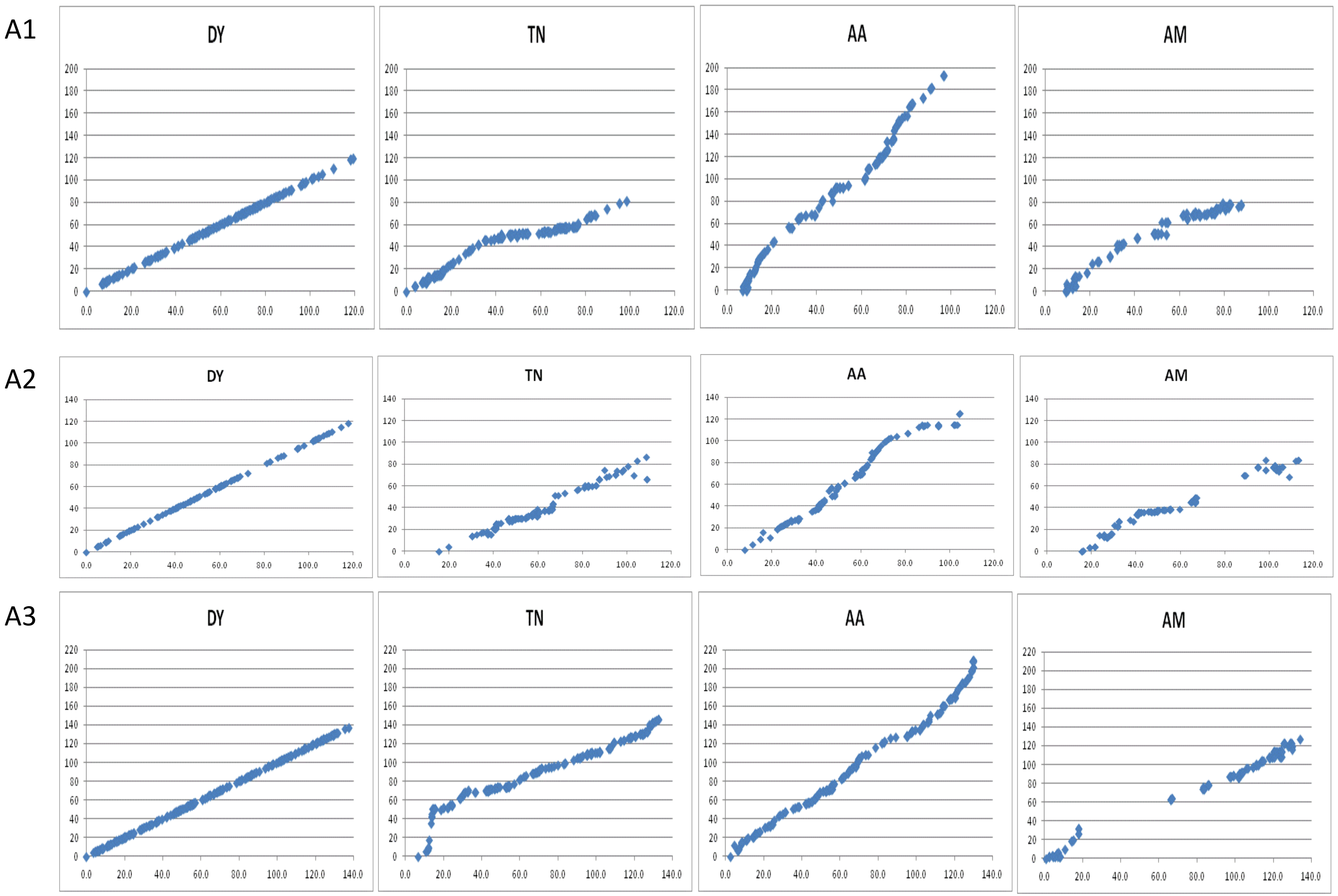
A1
DY
TN
AA
AM
A2
DY
TN
AA
AM
A3
DY
TN
AA
AM

A4

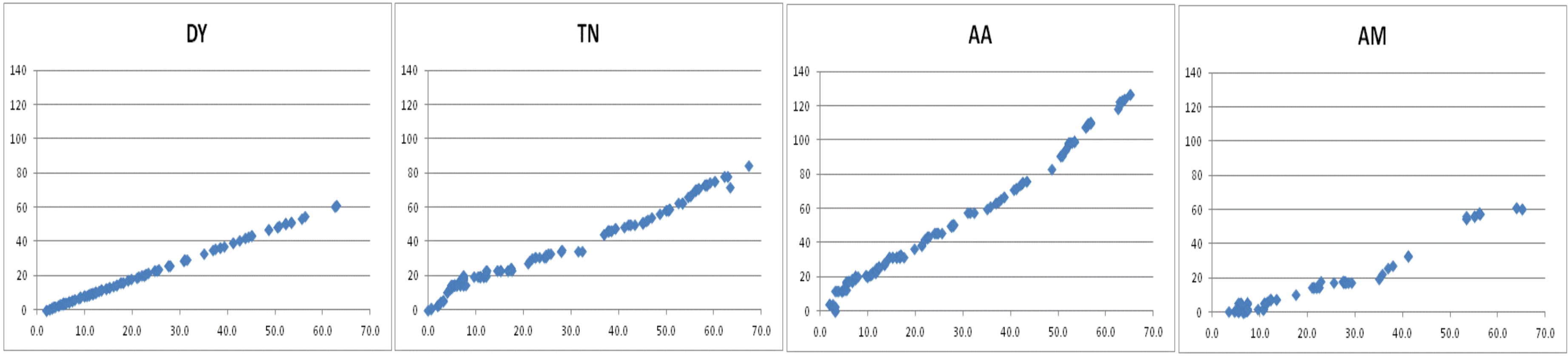
DY
TN
AA
AM
0 20 40 60 80 100 120 140
0.0 10.0 20.0 30.0 40.0 50.0 60.0 70.0

A5

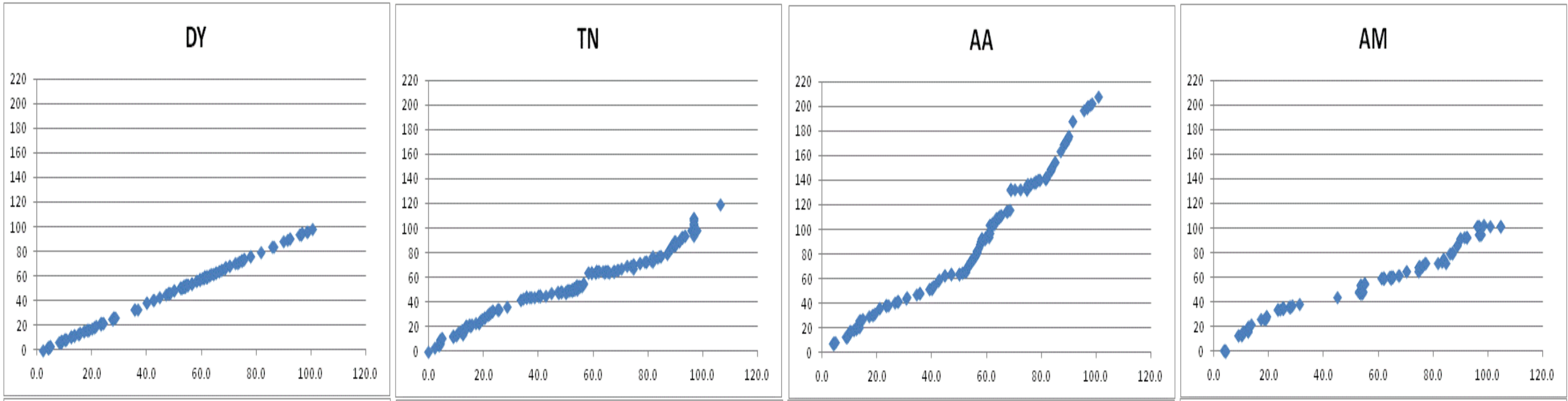
DY
TN
AA
AM
0 20 40 60 80 100 120 140 160 180 200 220
0.0 20.0 40.0 60.0 80.0 100.0 120.0

A6

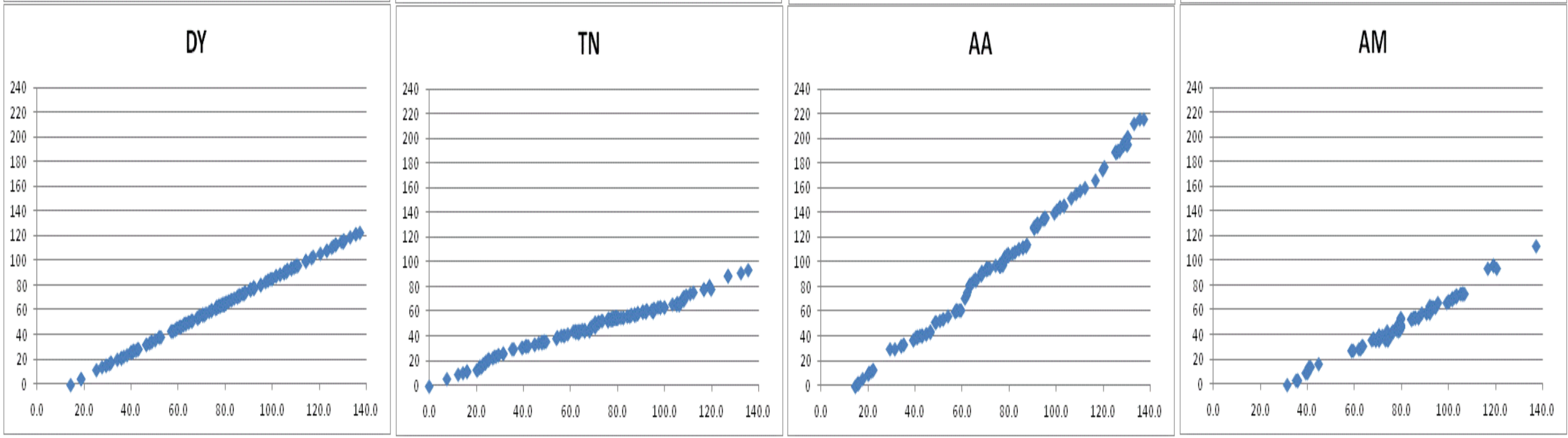
DY
TN
AA
AM
0 20 40 60 80 100 120 140 160 180 200 220 240
0.0 20.0 40.0 60.0 80.0 100.0 120.0 140.0

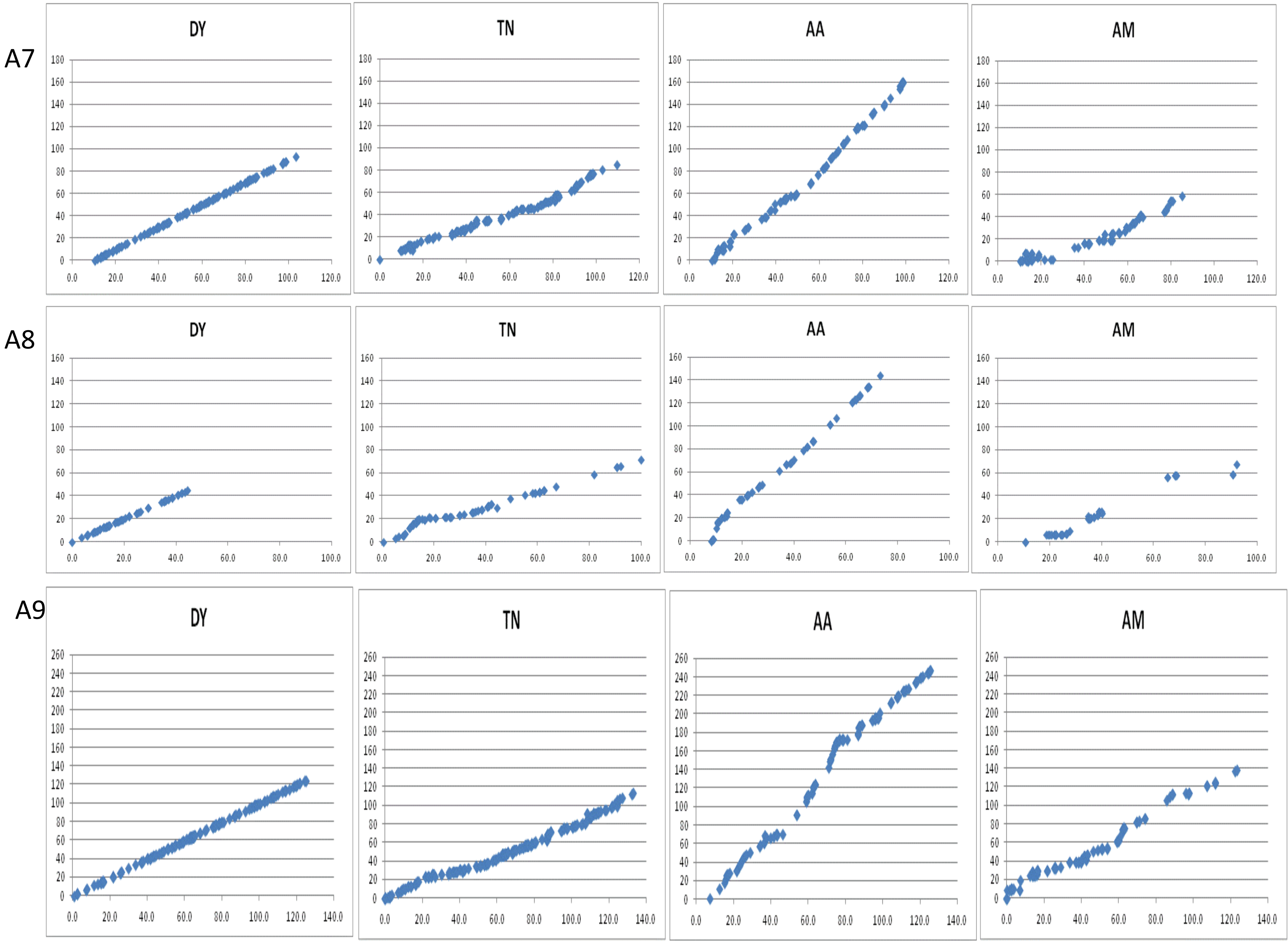
A7
DY
TN
AA
AM
180
160
140
120
100
80
60
40
20
0
0.0
20.0
40.0
60.0
80.0
100.0
120.0
A8
DY
TN
AA
AM
160
140
120
100
80
60
40
20
0
0.0
20.0
40.0
60.0
80.0
100.0
A9
DY
TN
AA
AM
260
240
220
200
180
160
140
120
100
80
60
40
20
0
0.0
20.0
40.0
60.0
80.0
100.0
120.0
140.0

A10

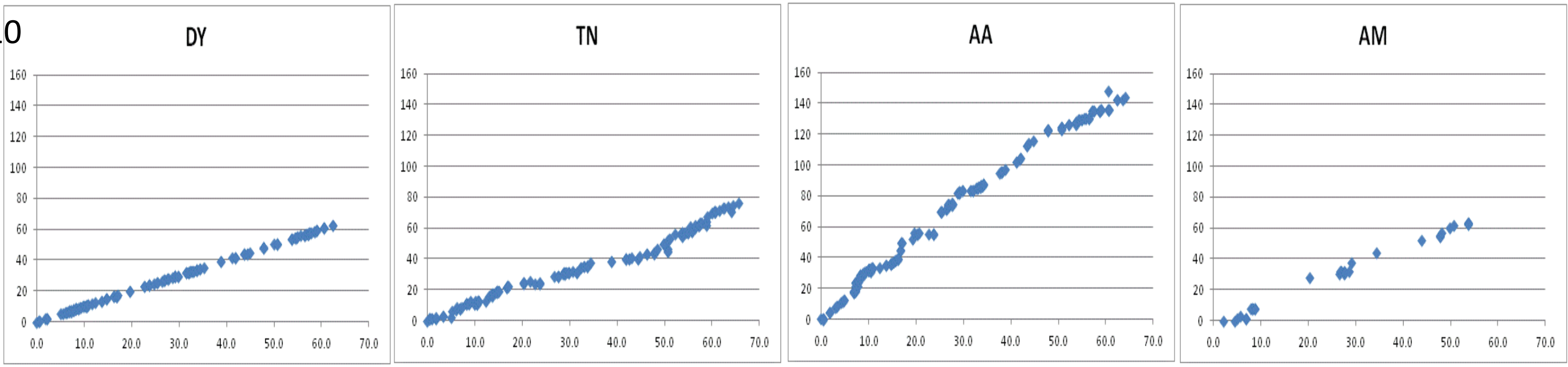
DY
TN
AA
AM
0
20
40
60
80
100
120
140
160
0.0
10.0
20.0
30.0
40.0
50.0
60.0
70.0

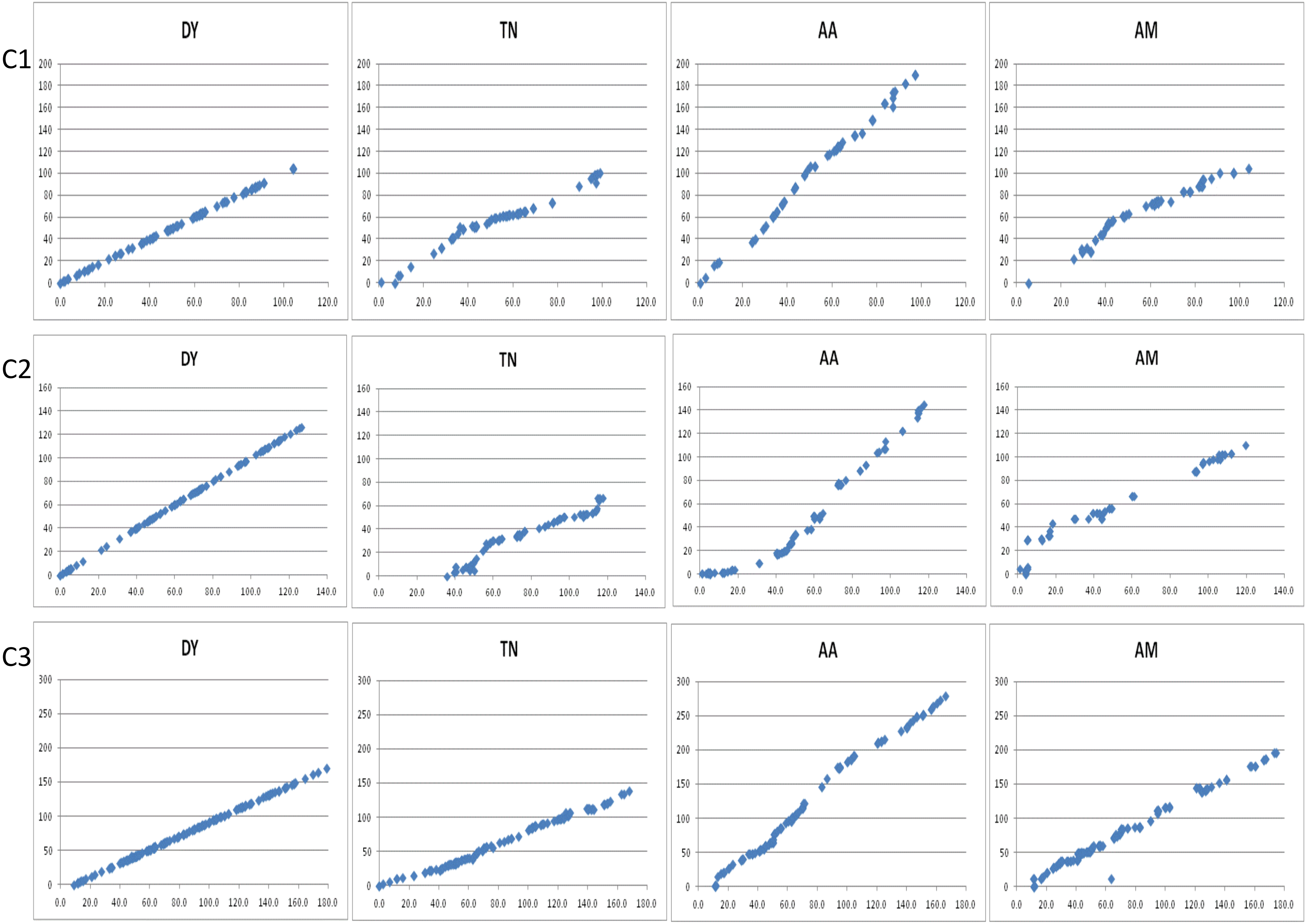

C1
C2
C3
DY
TN
AA
AM

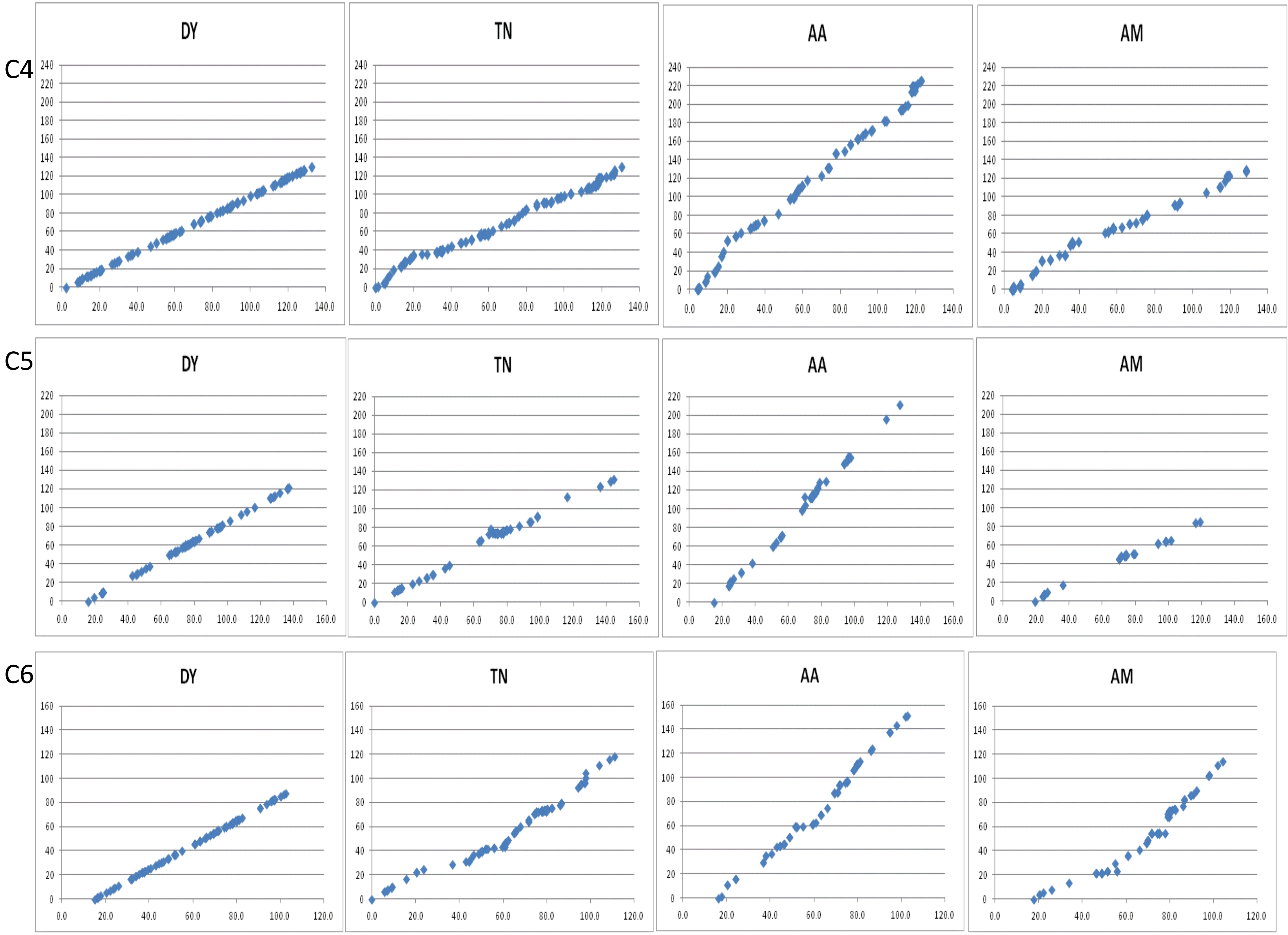
C4
DY
TN
AA
AM
C5
DY
TN
AA
AM
C6
DY
TN
AA
AM

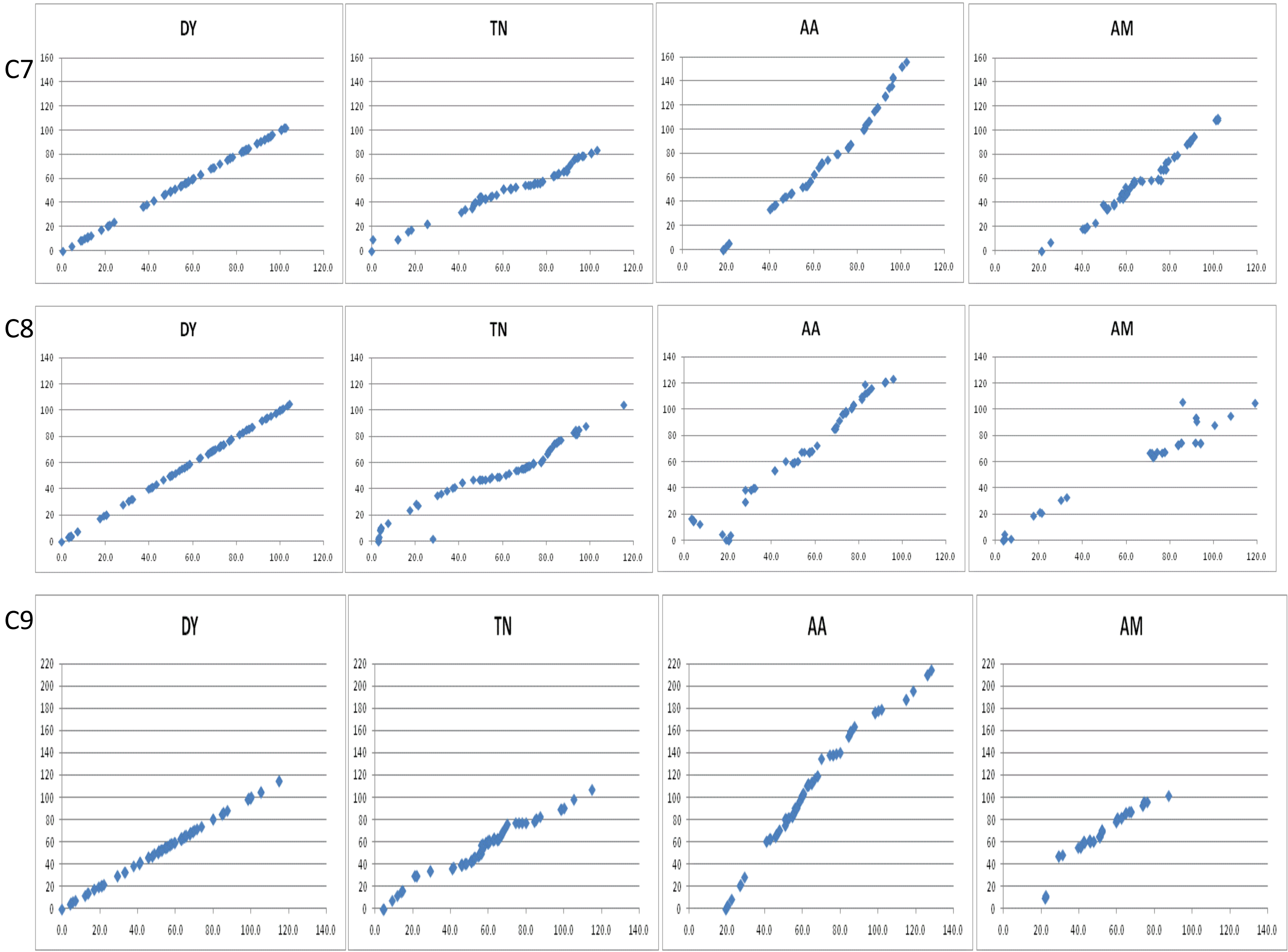
C7
DY
TN
AA
AM
C8
DY
TN
AA
AM
C9
DY
TN
AA
AM
