# Supplementary material for: High-density SNP-based genetic map development and linkage disequilibrium assessment in Brassica napus L
Source: BMC Genomics. 2013 Feb 22;14:120. doi: 10.1186/1471-2164-14-120 (PMC3600037; doi:10.1186/1471-2164-14-120)
Supplement: Additional file 7: Figure S3 — Distribution of PIC values along the linkage groups in the fodder rape (FO), spring (SOSR) and winter (WOSR) oilseed rape and the different seed quality subgroups (“++”,” 0+”,” 00”) within WOSR. PIC was averaged across a sliding window of 10 cM with a step of one cM. [file 1471-2164-14-120-S7.pdf]

Supplementary figure 3: Distribution of PIC values along the linkage groups in the fodder rape (FO), spring (SOSR) and winter (WOSR) oilseed rape and the different seed quality subgroups (++, 0+, 00) within WOSR. PIC was averaged across a sliding window of 10 cM with a step of one cM.

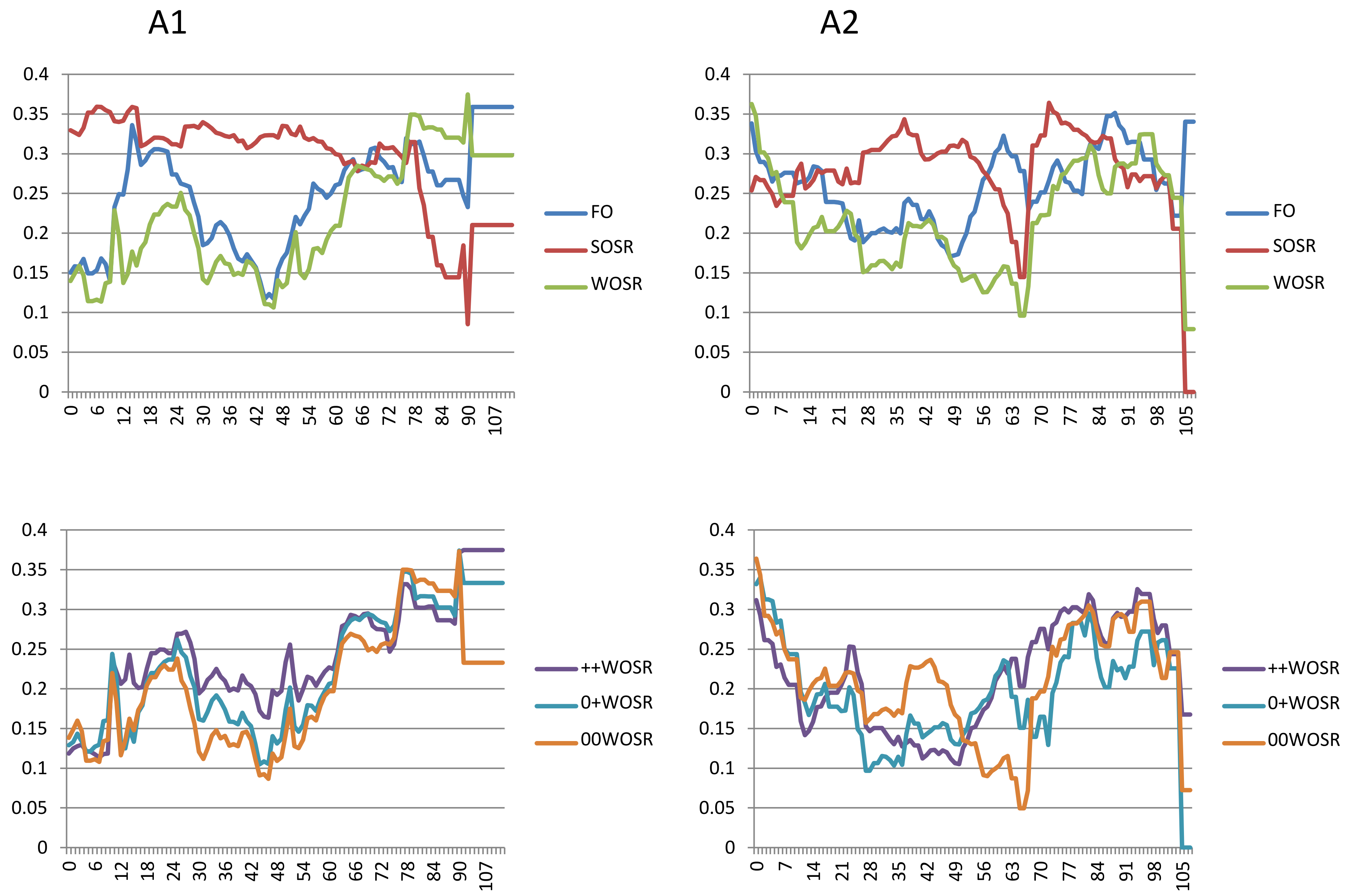

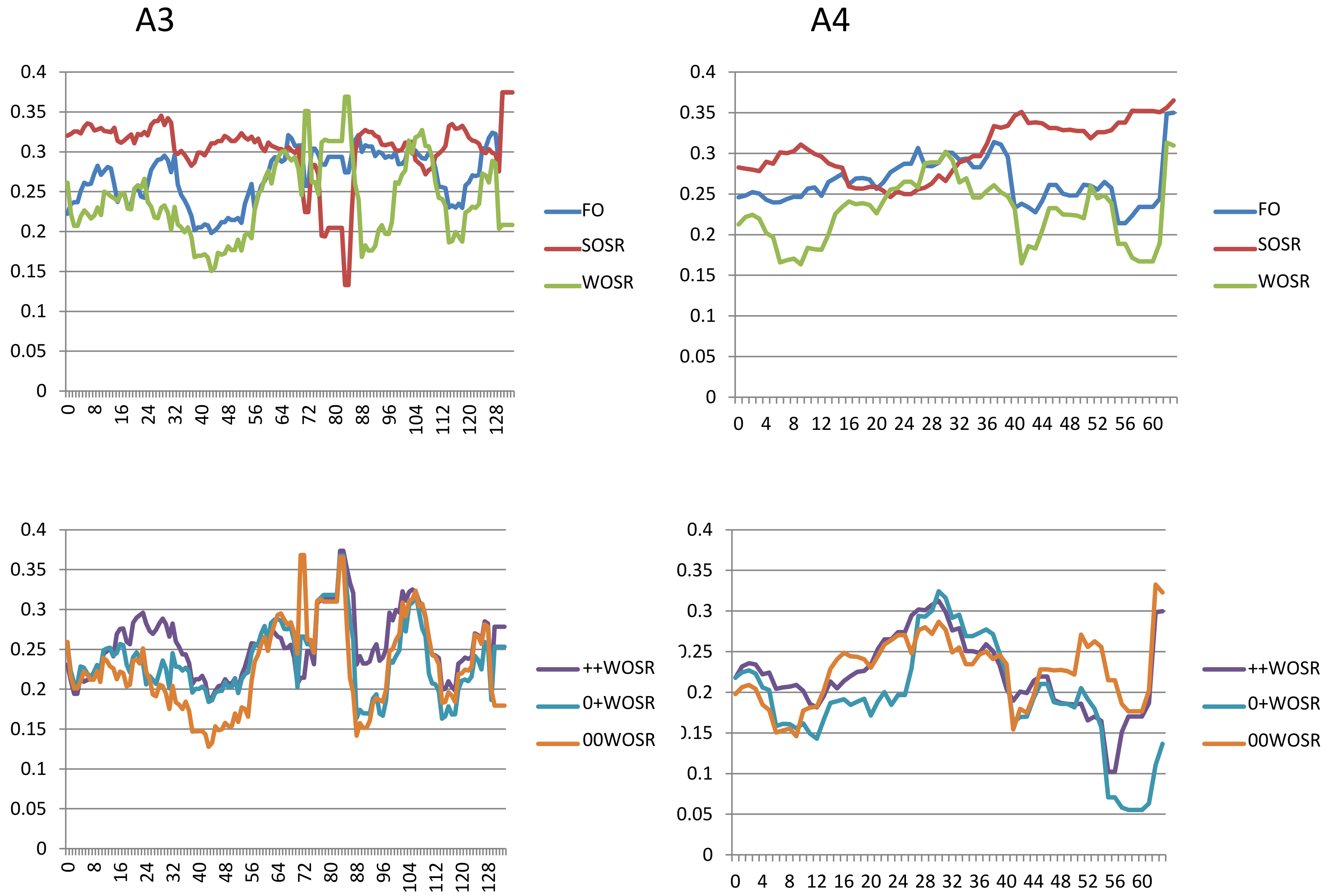
A3
A4
0.4
0.35
0.3
0.25
0.2
0.15
0.1
0.05
0
0 8 16 24 32 40 48 56 64 72 80 88 96 104 112 120 128
0 4 8 12 16 20 24 28 32 36 40 44 48 52 56 60
FO
SOSR
WOSR
++WOSR
0+WOSR
00WOSR

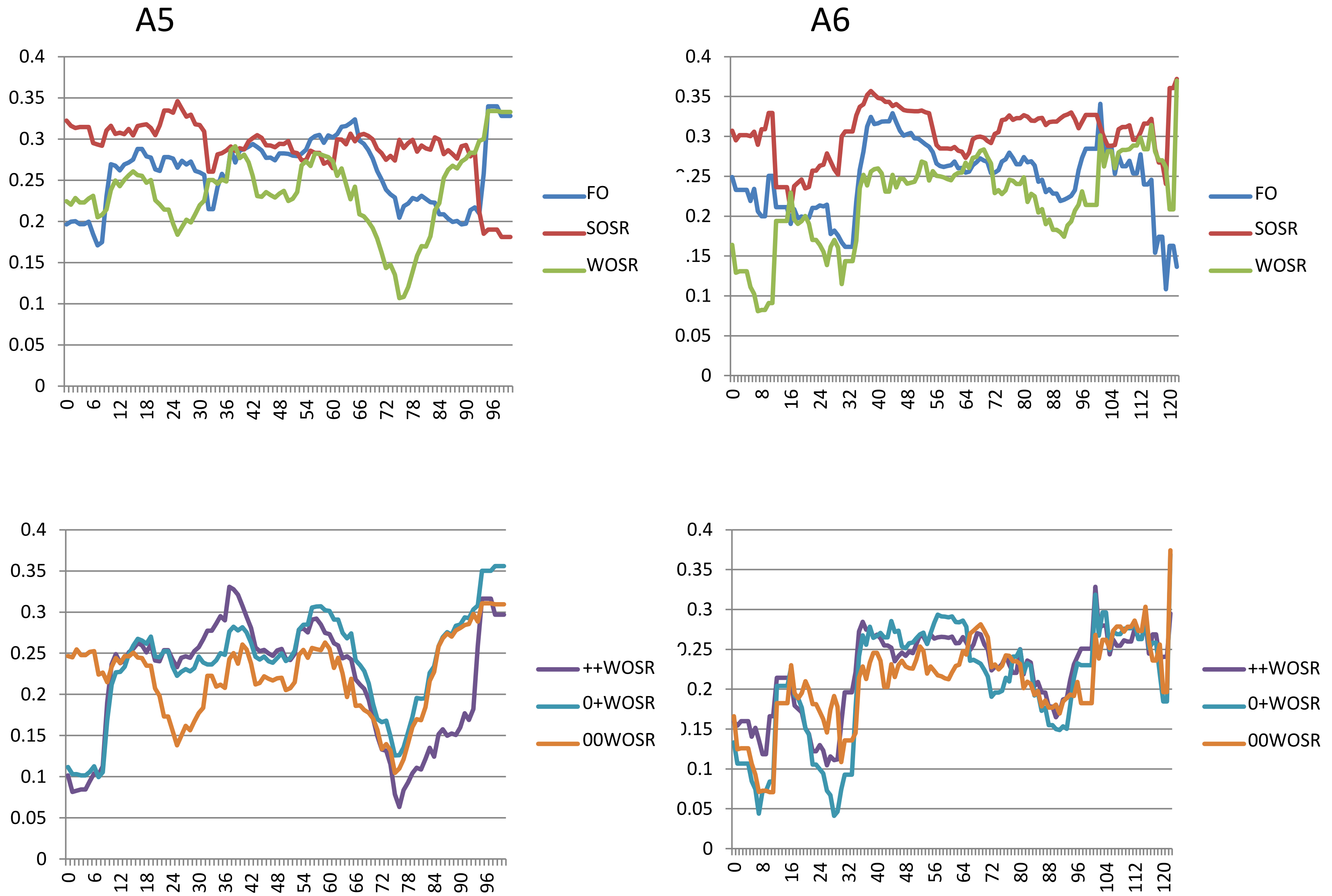
A5
A6
FO
SOSR
WOSR
++WOSR
0+WOSR
00WOSR

A7

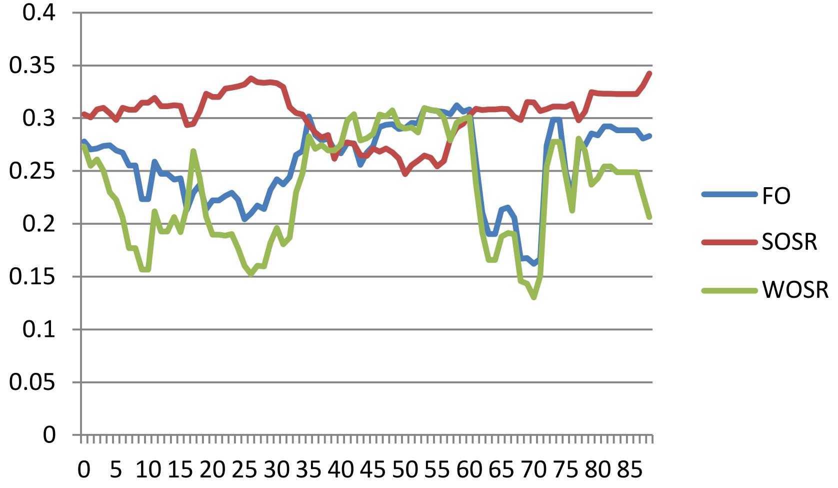
0.4
0.35
0.3
0.25
0.2
0.15
0.1
0.05
0
0 5 10 15 20 25 30 35 40 45 50 55 60 65 70 75 80 85
FO
SOSR
WOSR

A8

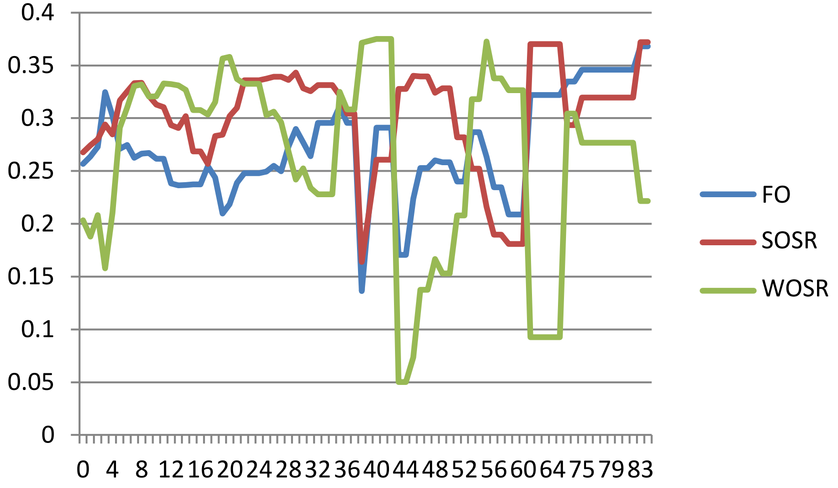
0.4
0.35
0.3
0.25
0.2
0.15
0.1
0.05
0
0 4 8 12 16 20 24 28 32 36 40 44 48 52 56 60 64 75 79 83
FO
SOSR
WOSR

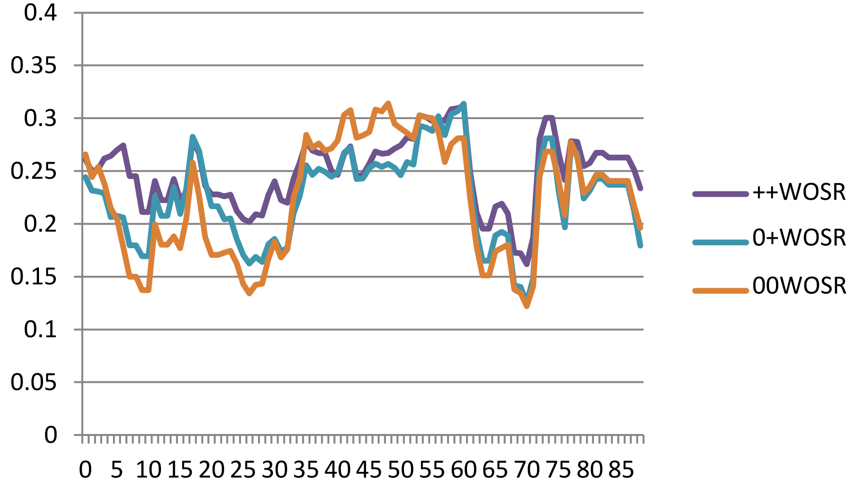
0.4
0.35
0.3
0.25
0.2
0.15
0.1
0.05
0
0 5 10 15 20 25 30 35 40 45 50 55 60 65 70 75 80 85
++WOSR
0+WOSR
00WOSR

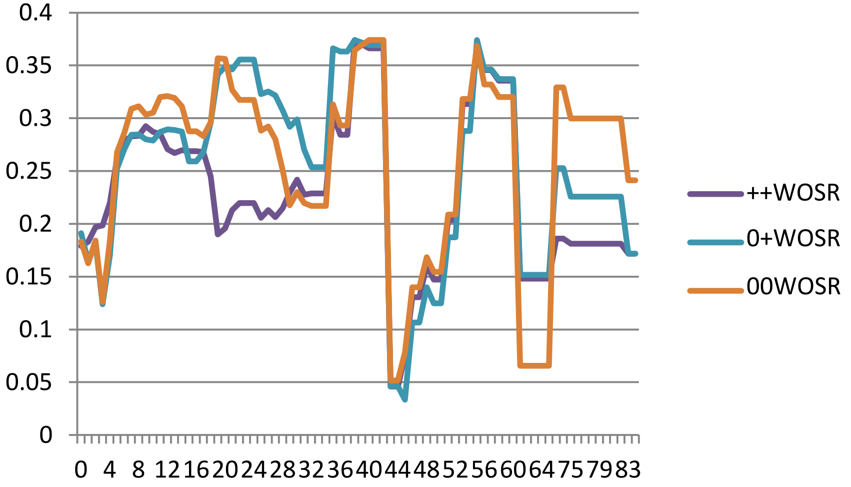
0.4
0.35
0.3
0.25
0.2
0.15
0.1
0.05
0
0 4 8 12 16 20 24 28 32 36 40 44 48 52 56 60 64 75 79 83
++WOSR
0+WOSR
00WOSR

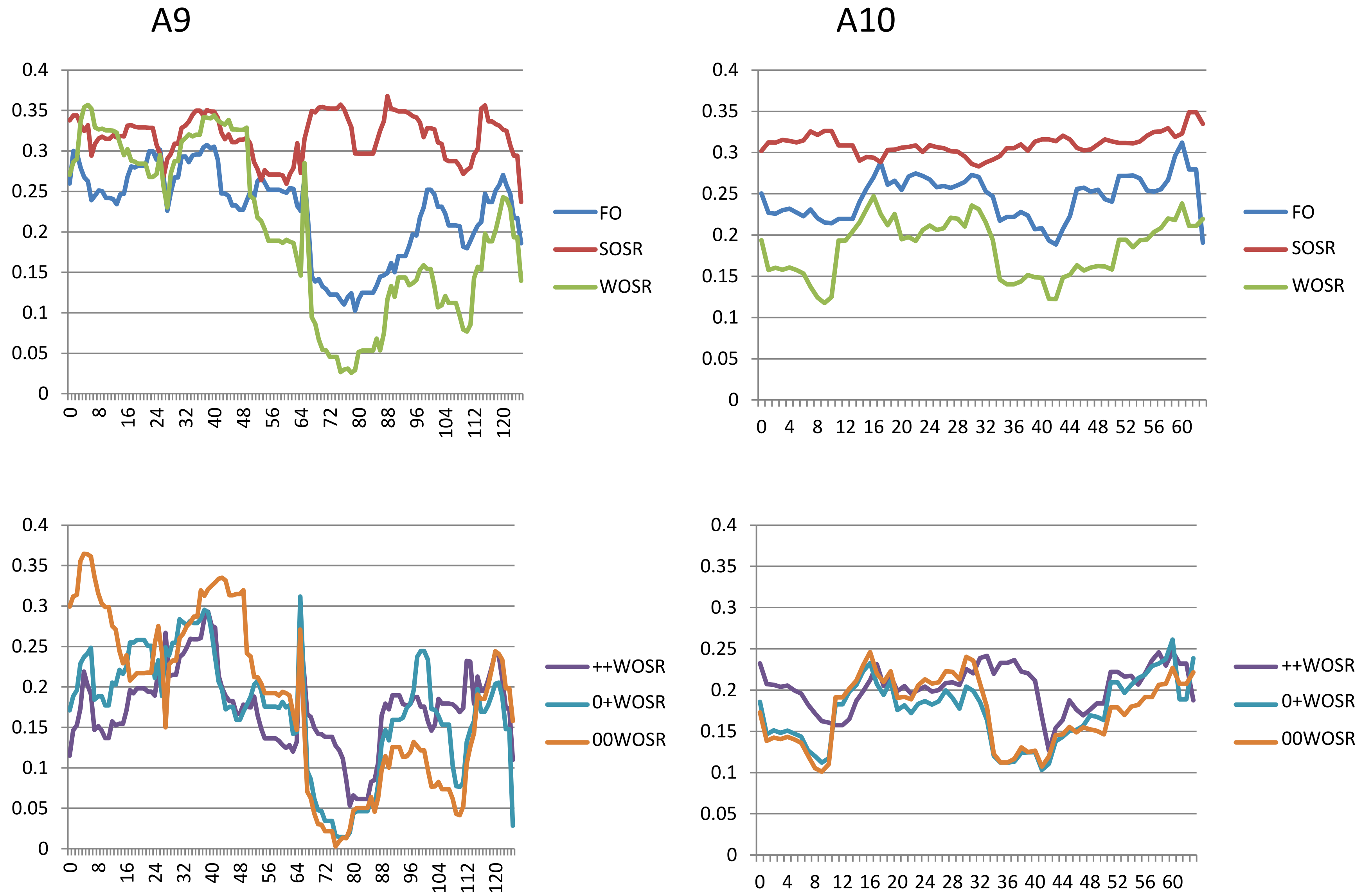

A9
A10
FO
SOSR
WOSR
++WOSR
0+WOSR
00WOSR
0.4
0.35
0.3
0.25
0.2
0.15
0.1
0.05
0
0 8 16 24 32 40 48 56 64 72 80 88 96 104 112 120
0 4 8 12 16 20 24 28 32 36 40 44 48 52 56 60

C1 C2

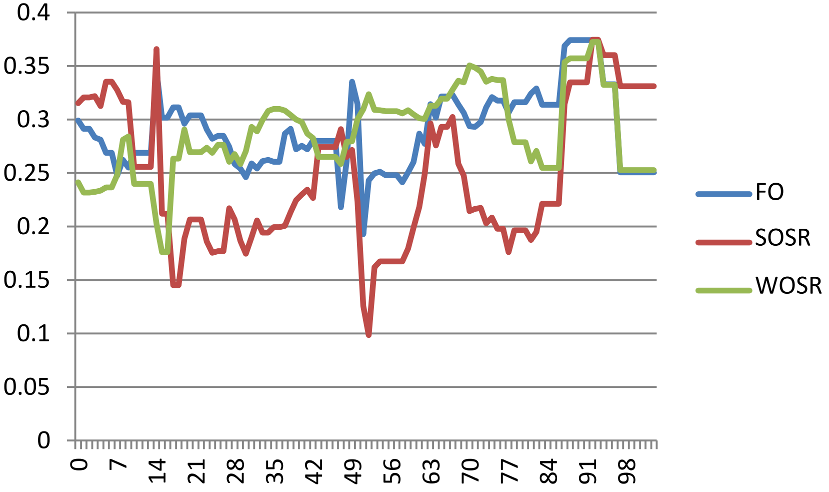
0.4
0.35
0.3
0.25
0.2
0.15
0.1
0.05
0
0 7 14 21 28 35 42 49 56 63 70 77 84 91 98
FO
SOSR
WOSR

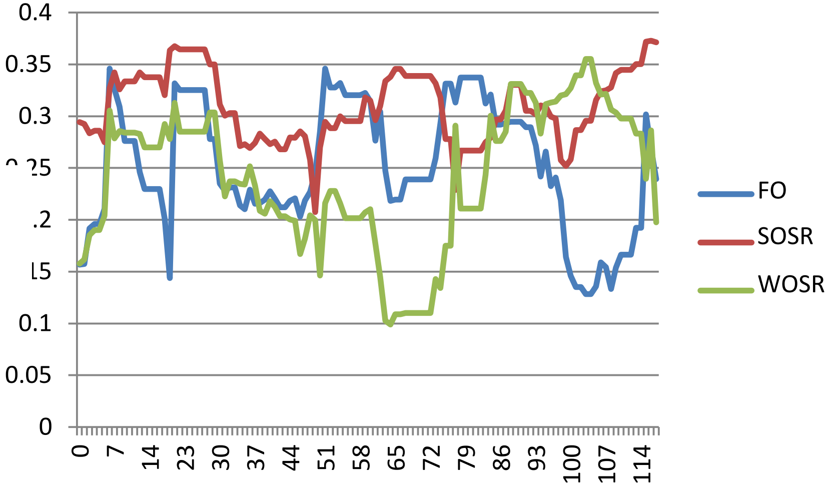
0.4
0.35
0.3
0.1
0.05
0
0 7 14 23 30 37 44 51 58 65 72 79 86 93 100 107 114
FO
SOSR
WOSR

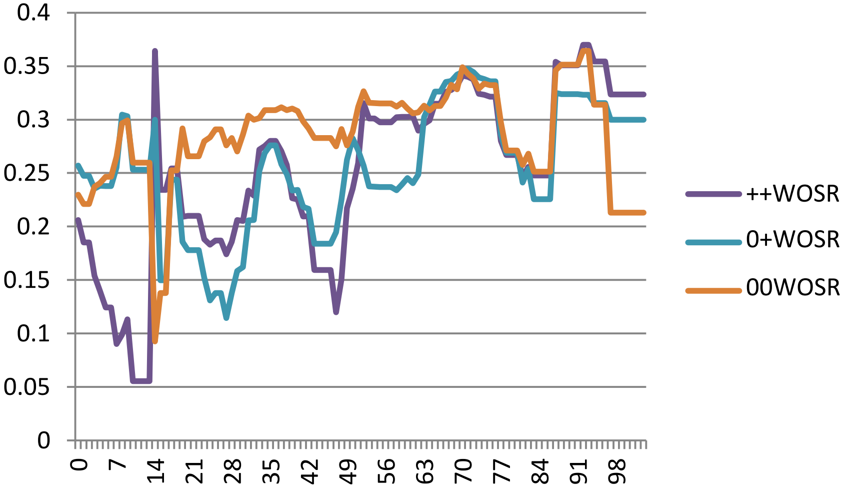
0.4
0.35
0.3
0.25
0.2
0.15
0.1
0.05
0
0 7 14 21 28 35 42 49 56 63 70 77 84 91 98
++WOSR
0+WOSR
00WOSR

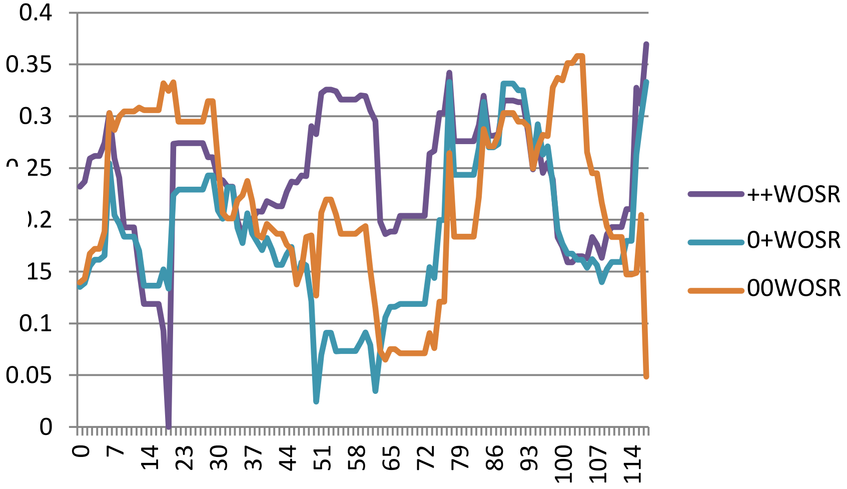
0.4
0.35
0.3
0.1
0.05
0
0 7 14 23 30 37 44 51 58 65 72 79 86 93 100 107 114
++WOSR
0+WOSR
00WOSR

C3

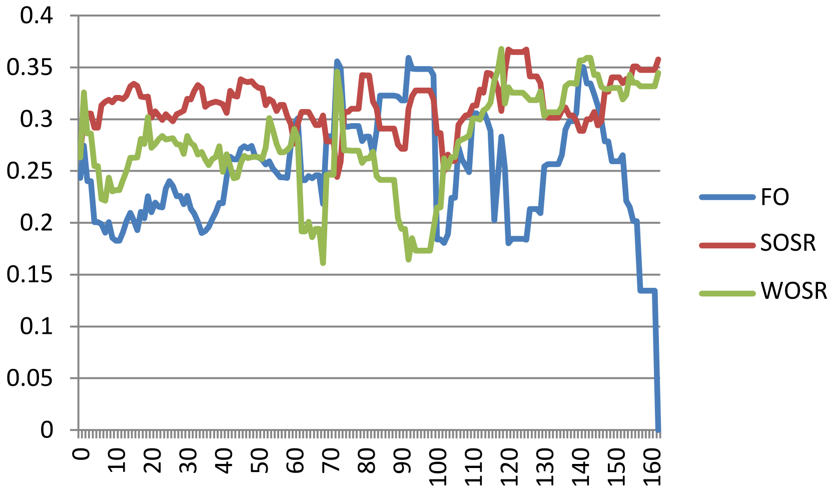
0.4
0.35
0.3
0.25
0.2
0.15
0.1
0.05
0
0 10 20 30 40 50 60 70 80 90 100 110 120 130 140 150 160
FO
SOSR
WOSR

C4

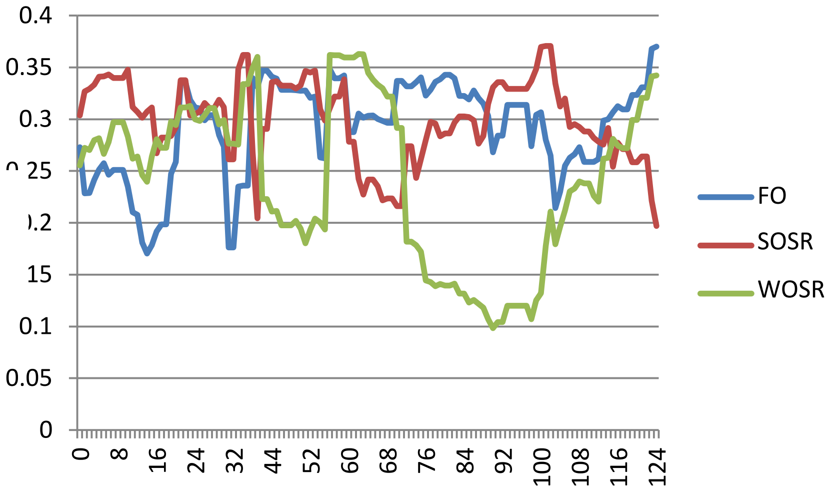
0.4
0.35
0.3
0.1
0.05
0
0 8 16 24 32 44 52 60 68 76 84 92 100 108 116 124
FO
SOSR
WOSR

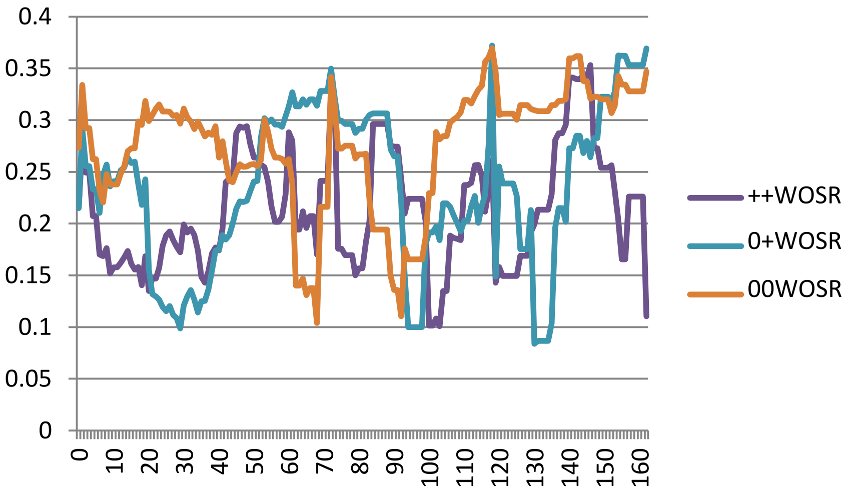
0.4
0.35
0.3
0.25
0.2
0.15
0.1
0.05
0
0 10 20 30 40 50 60 70 80 90 100 110 120 130 140 150 160
++WOSR
0+WOSR
00WOSR

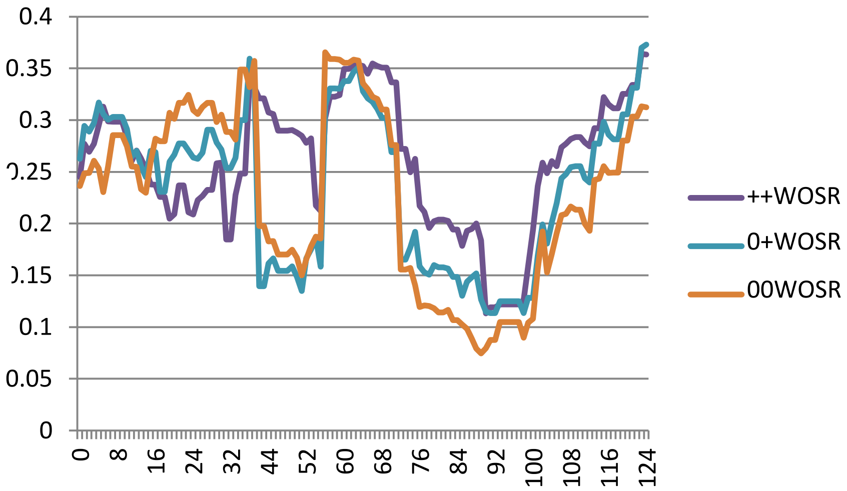
0.4
0.35
0.3
0.2
0.1
0.05
0
0 8 16 24 32 44 52 60 68 76 84 92 100 108 116 124
++WOSR
0+WOSR
00WOSR

C5

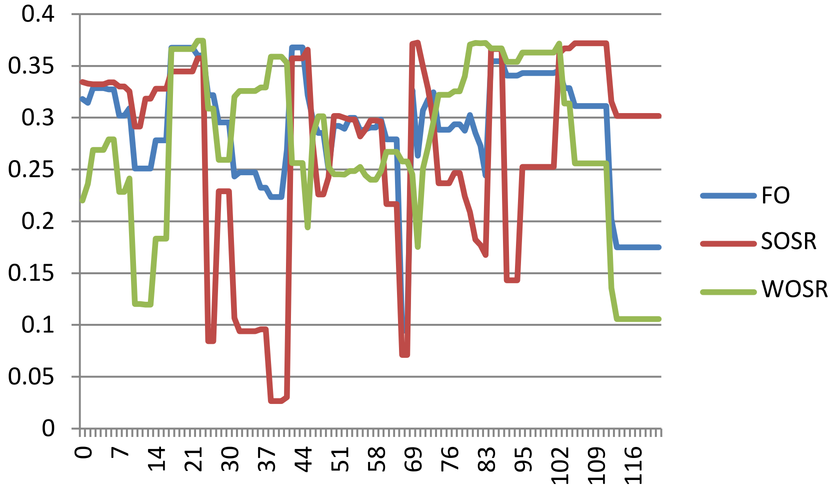
0.4
0.35
0.3
0.25
0.2
0.15
0.1
0.05
0
0 7 14 21 30 37 44 51 58 69 76 83 95 102 109 116
FO
SOSR
WOSR

C6

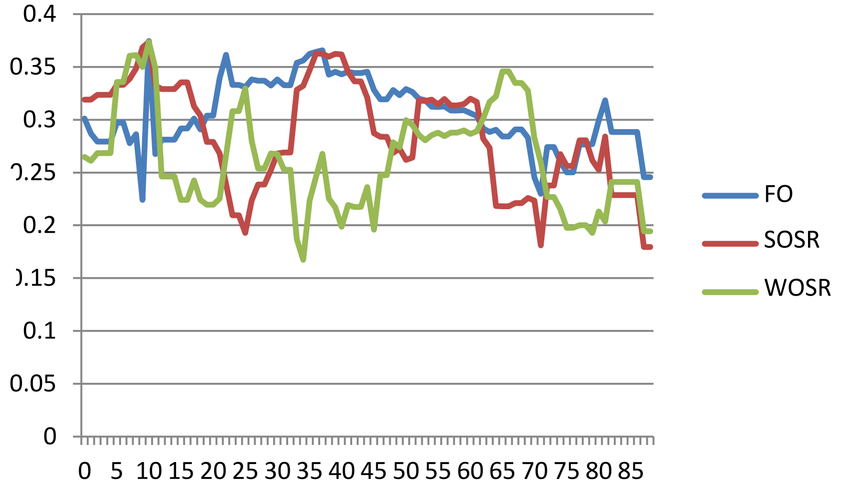
0.4
0.35
0.3
).25
0.2
).15
0.1
0.05
0
0 5 10 15 20 25 30 35 40 45 50 55 60 65 70 75 80 85
FO
SOSR
WOSR

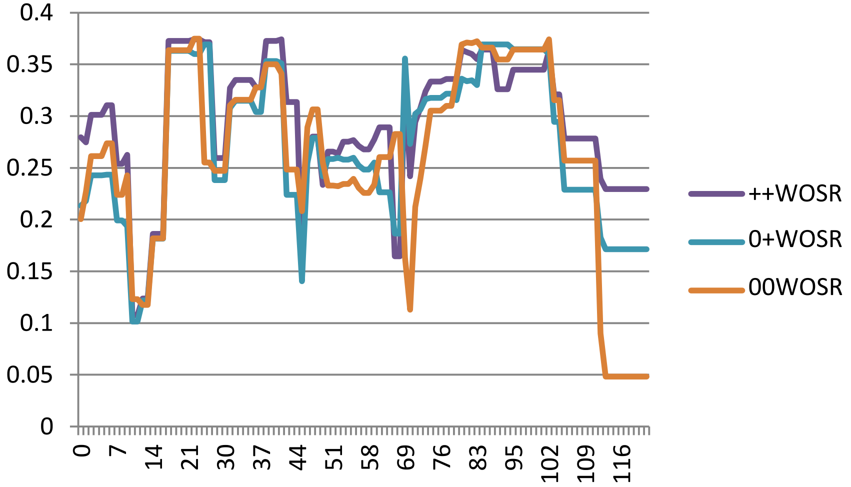
0.4
0.35
0.3
0.25
0.2
0.15
0.1
0.05
0
0 7 14 21 30 37 44 51 58 69 76 83 95 102 109 116
++WOSR
0+WOSR
00WOSR

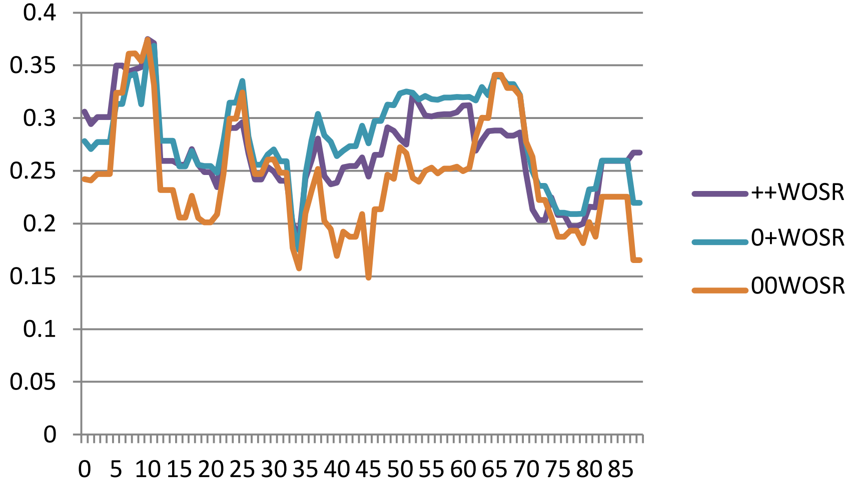
0.4
0.35
0.3
0.25
0.2
0.15
0.1
0.05
0
0 5 10 15 20 25 30 35 40 45 50 55 60 65 70 75 80 85
++WOSR
0+WOSR
00WOSR

C7 C8

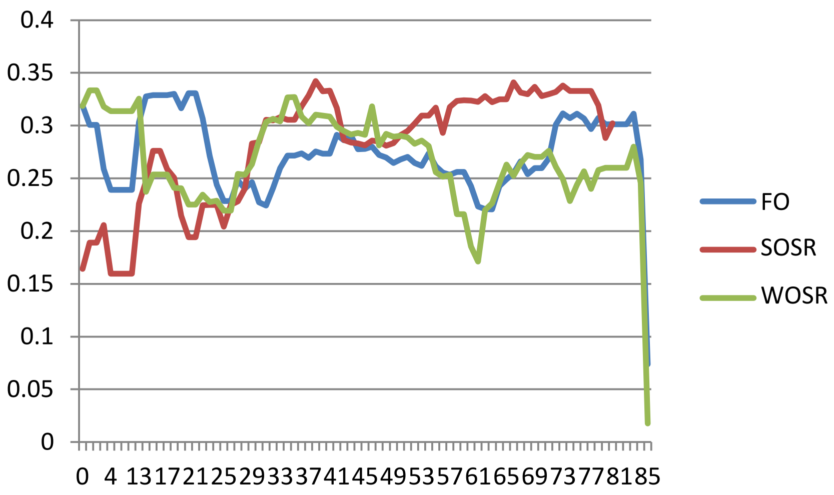
0.4
0.35
0.3
0.25
0.2
0.15
0.1
0.05
0
0 4 131721252933374145495357616569737778185
FO
SOSR
WOSR

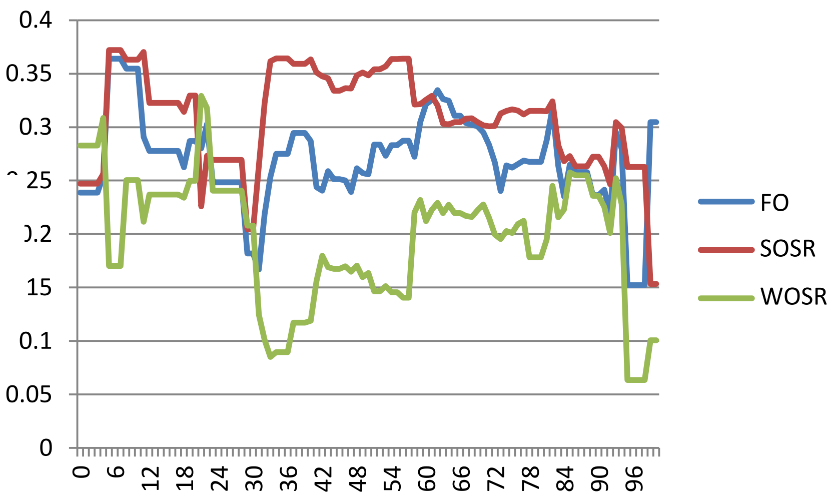
0.4
0.35
0.3
0.1
0.05
0
0 6 12 18 24 30 36 42 48 54 60 66 72 78 84 90 96
FO
SOSR
WOSR

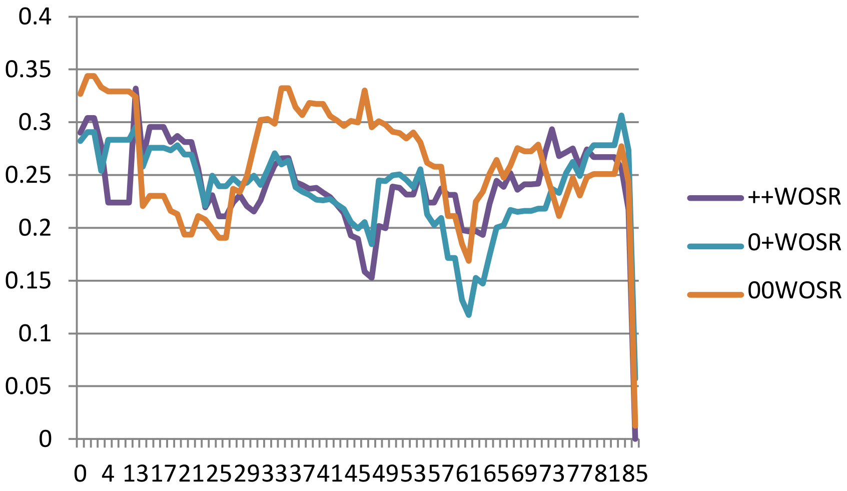
0.4
0.35
0.3
0.25
0.2
0.15
0.1
0.05
0
0 4 131721252933374145495357616569737778185
++WOSR
0+WOSR
00WOSR

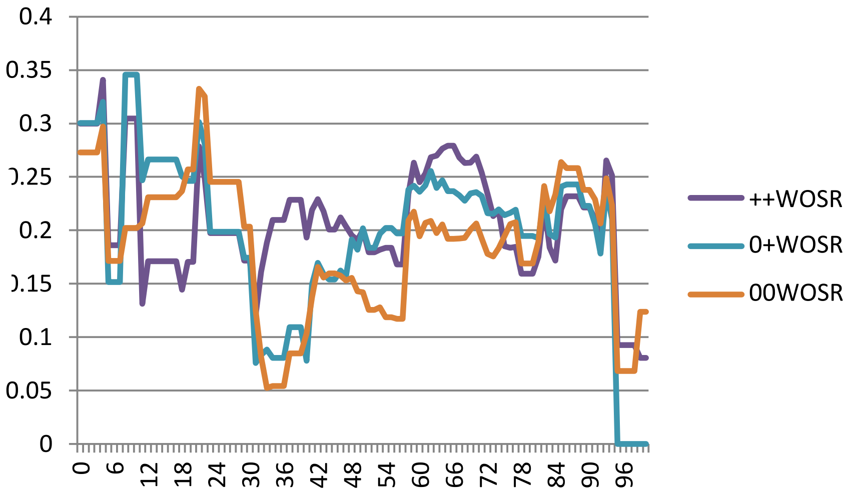
0.4
0.35
0.3
0.2
0.1
0.05
0
0 6 12 18 24 30 36 42 48 54 60 66 72 78 84 90 96
++WOSR
0+WOSR
00WOSR

C9

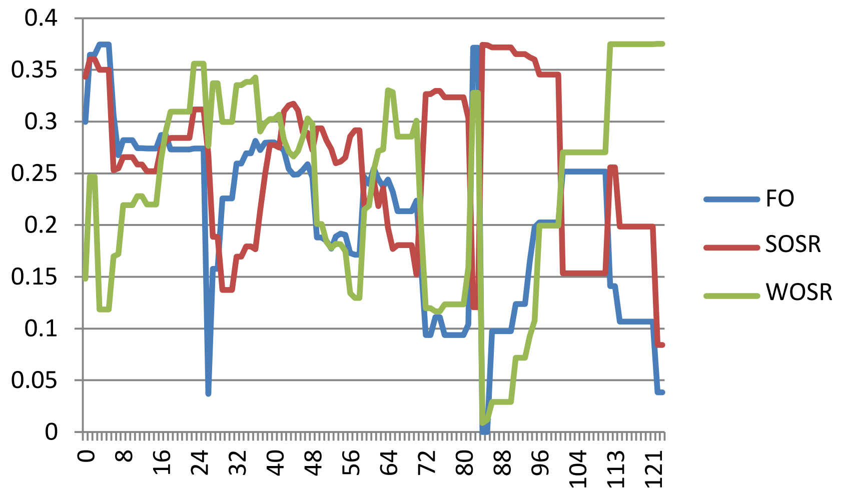
0.4
0.35
0.3
0.25
0.2
0.15
0.1
0.05
0
0 8 16 24 32 40 48 56 64 72 80 88 96 104 113 121
FO
SOSR
WOSR

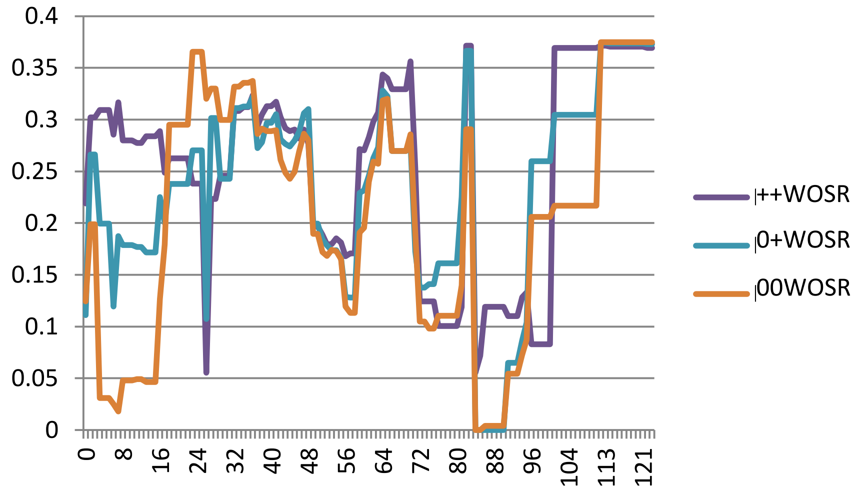
0.4
0.35
0.3
0.25
0.2
0.15
0.1
0.05
0
0 8 16 24 32 40 48 56 64 72 80 88 96 104 113 121
++WOSR
0+WOSR
00WOSR
