# Supplementary material for: High-density SNP-based genetic map development and linkage disequilibrium assessment in Brassica napus L
Source: BMC Genomics. 2013 Feb 22;14:120. doi: 10.1186/1471-2164-14-120 (PMC3600037; doi:10.1186/1471-2164-14-120)
Supplement: Additional file 8: Figure S4 — Plots of r2 as a function of genetic distance (in cM) between pairs of SNPs on each linkage group in the whole, spring, winter and “00” winter oilseed rape collections. Red curves show the non-linear regressions trend line of r2 versus genetic distance. [file 1471-2164-14-120-S8.pdf]

**Supplementary figure 4:** Plots of r² as a function of genetic distance (in cM) between pairs of SNPs on each linkage group in the whole, spring, winter and « 00 » winter oilseed rape collections. Red curves show the non-inear regressions trend line of r² versus genetic distance

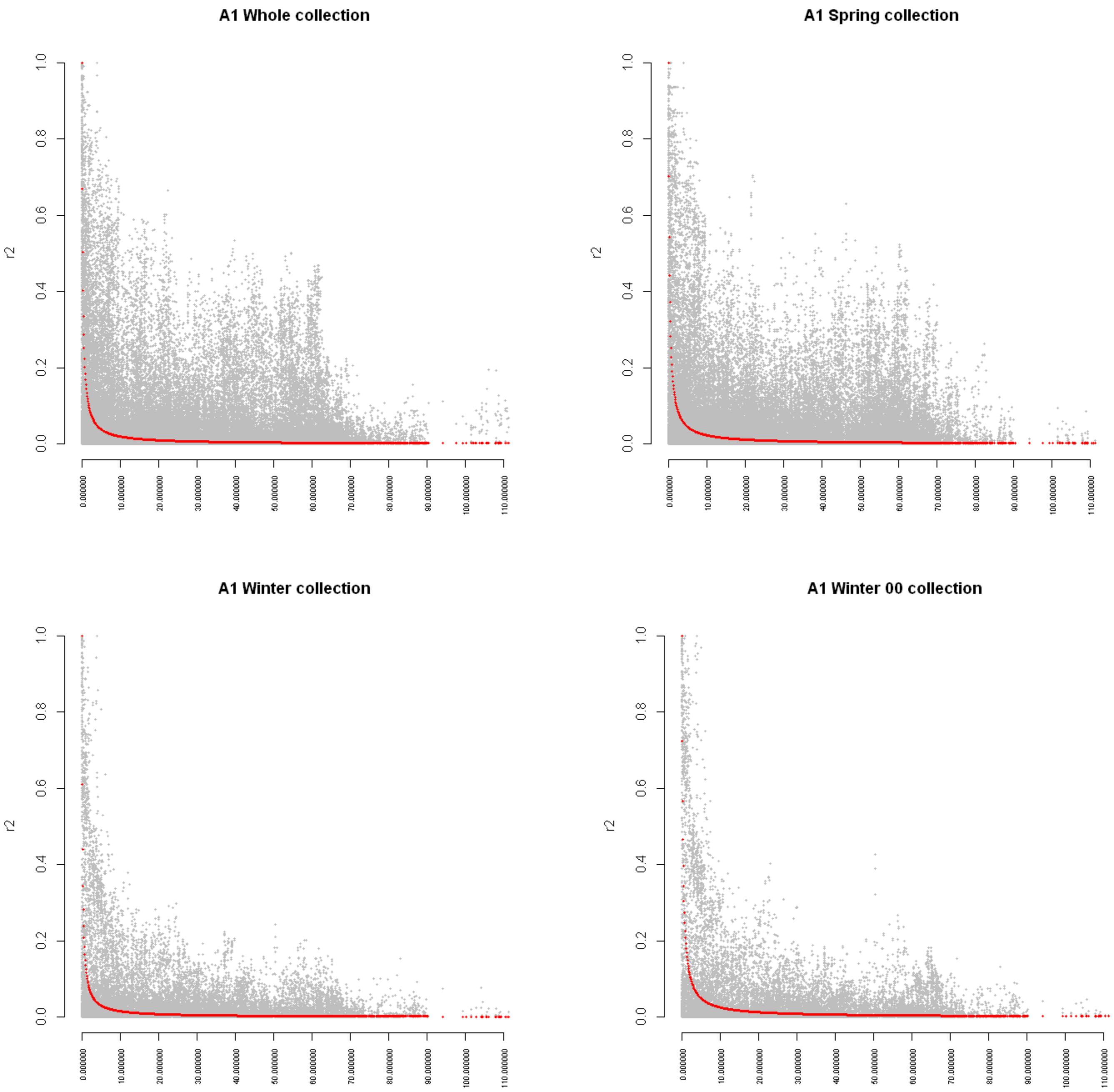

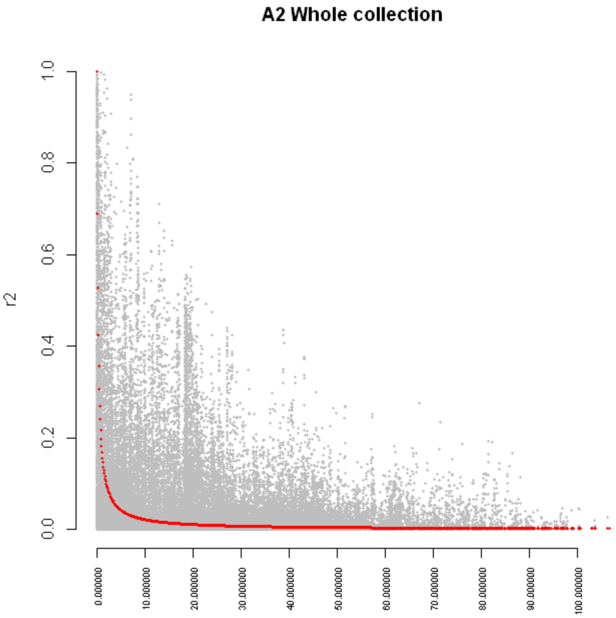
A2 Whole collection
r2
0.0
0.2
0.4
0.6
0.8
1.0
0.000000
10.000000
20.000000
30.000000
40.000000
50.000000
60.000000
70.000000
80.000000
90.000000
100.000000

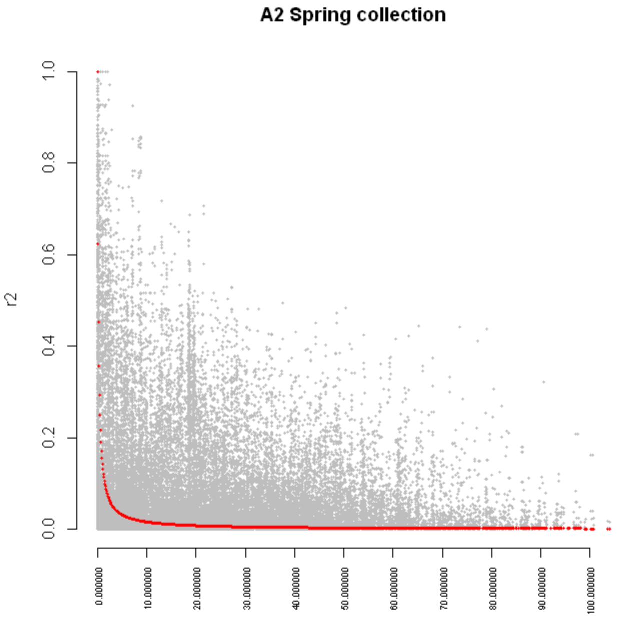
A2 Spring collection
r2
0.0
0.2
0.4
0.6
0.8
1.0
0.000000
10.000000
20.000000
30.000000
40.000000
50.000000
60.000000
70.000000
80.000000
90.000000
100.000000

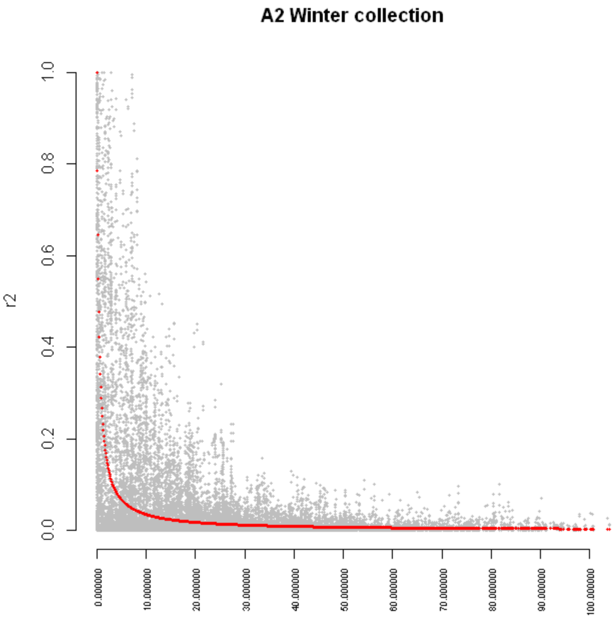
A2 Winter collection
r2
0.0
0.2
0.4
0.6
0.8
1.0
0.000000
10.000000
20.000000
30.000000
40.000000
50.000000
60.000000
70.000000
80.000000
90.000000
100.000000

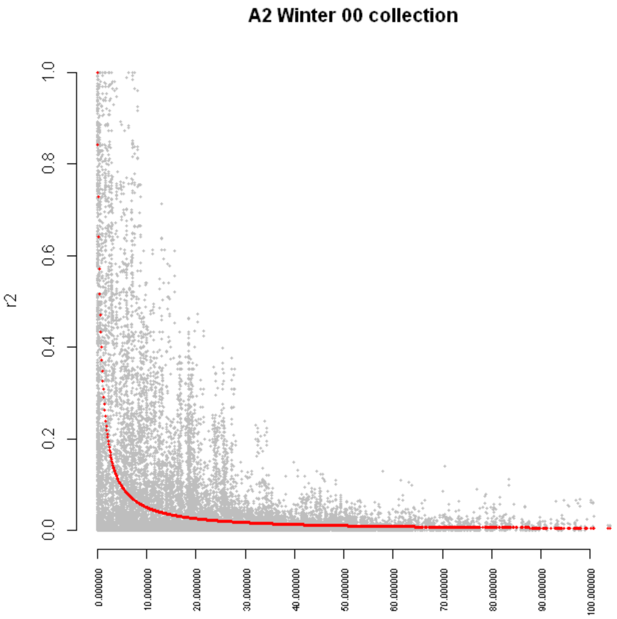
A2 Winter 00 collection
r2
0.0
0.2
0.4
0.6
0.8
1.0
0.000000
10.000000
20.000000
30.000000
40.000000
50.000000
60.000000
70.000000
80.000000
90.000000
100.000000

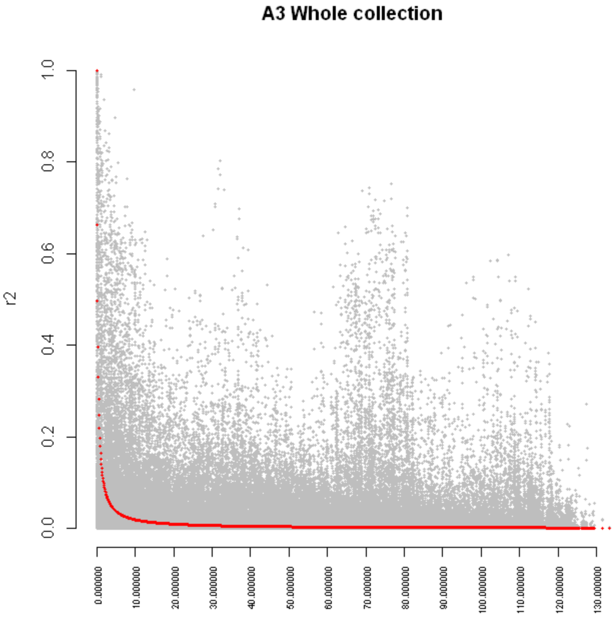
A3 Whole collection
r2
0.0
0.2
0.4
0.6
0.8
1.0
0.000000
10.000000
20.000000
30.000000
40.000000
50.000000
60.000000
70.000000
80.000000
90.000000
100.000000
110.000000
120.000000
130.000000

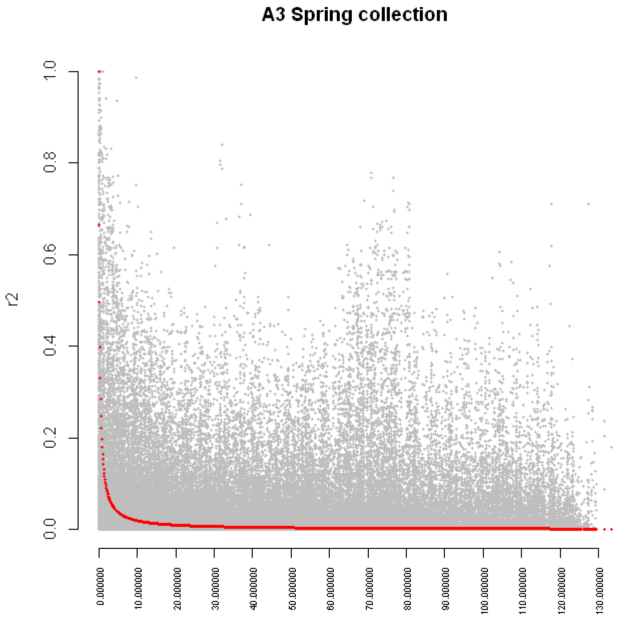
A3 Spring collection
r2
0.0
0.2
0.4
0.6
0.8
1.0
0.000000
10.000000
20.000000
30.000000
40.000000
50.000000
60.000000
70.000000
80.000000
90.000000
100.000000
110.000000
120.000000
130.000000

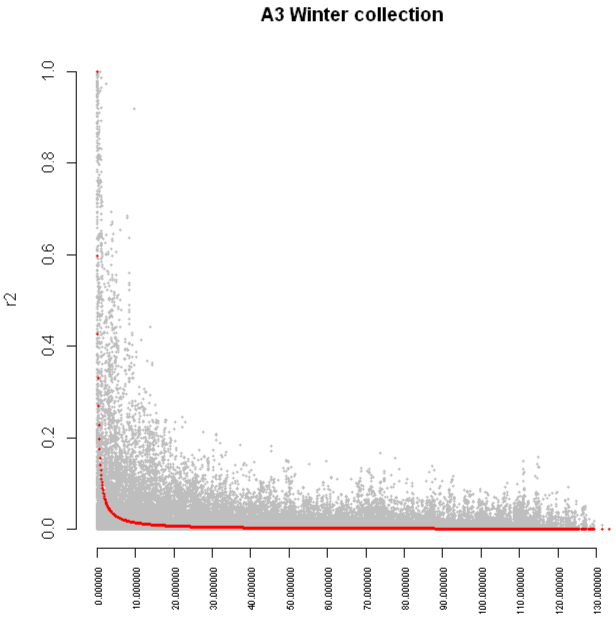
A3 Winter collection
r2
0.0
0.2
0.4
0.6
0.8
1.0
0.000000
10.000000
20.000000
30.000000
40.000000
50.000000
60.000000
70.000000
80.000000
90.000000
100.000000
110.000000
120.000000
130.000000

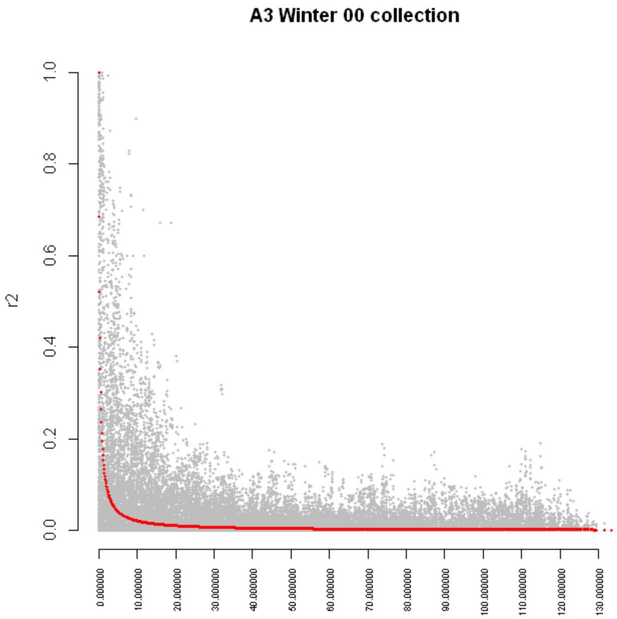
A3 Winter 00 collection
r2
0.0
0.2
0.4
0.6
0.8
1.0
0.000000
10.000000
20.000000
30.000000
40.000000
50.000000
60.000000
70.000000
80.000000
90.000000
100.000000
110.000000
120.000000
130.000000

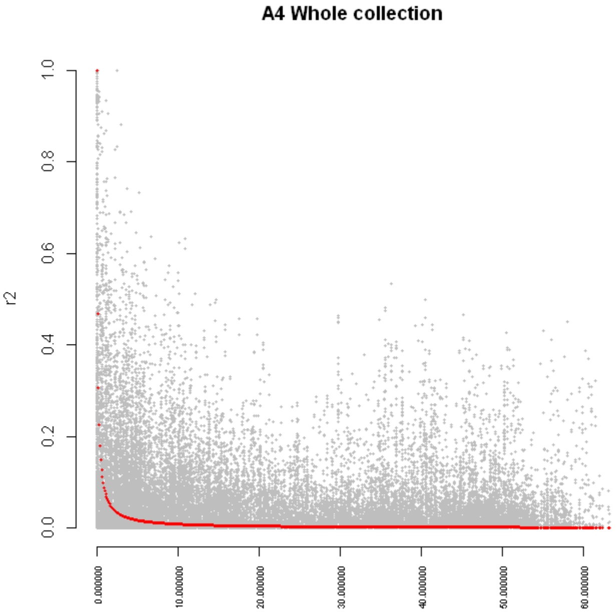
A4 Whole collection
r2
0.0
0.2
0.4
0.6
0.8
1.0
0.000000
10.000000
20.000000
30.000000
40.000000
50.000000
60.000000

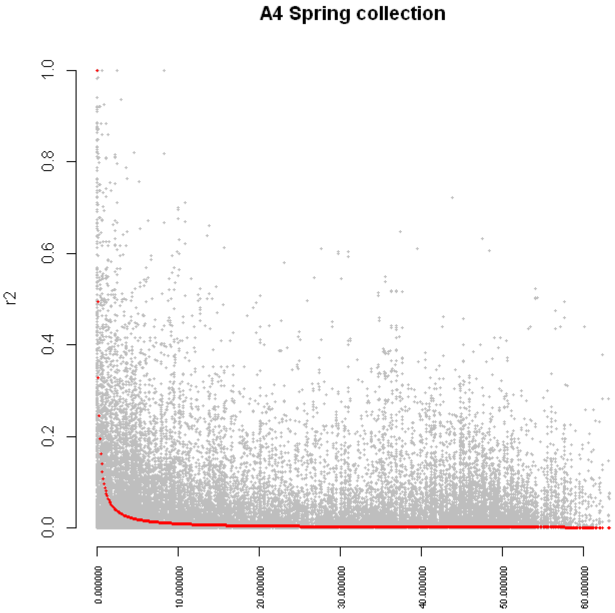
A4 Spring collection
r2
0.0
0.2
0.4
0.6
0.8
1.0
0.000000
10.000000
20.000000
30.000000
40.000000
50.000000
60.000000

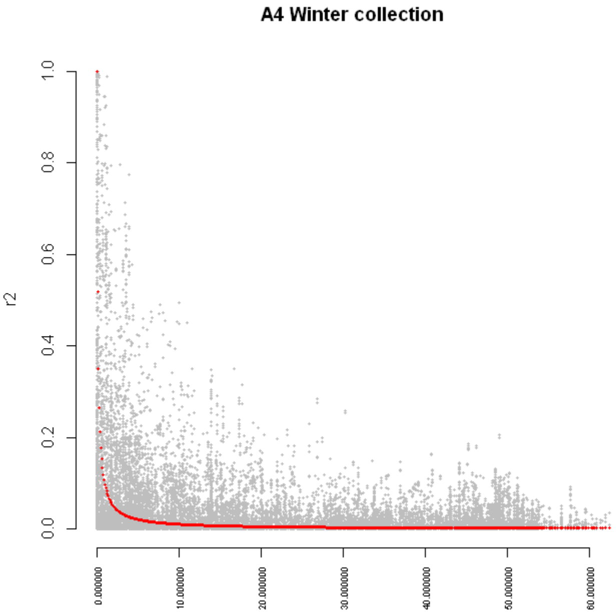
A4 Winter collection
r2
0.0
0.2
0.4
0.6
0.8
1.0
0.000000
10.000000
20.000000
30.000000
40.000000
50.000000
60.000000

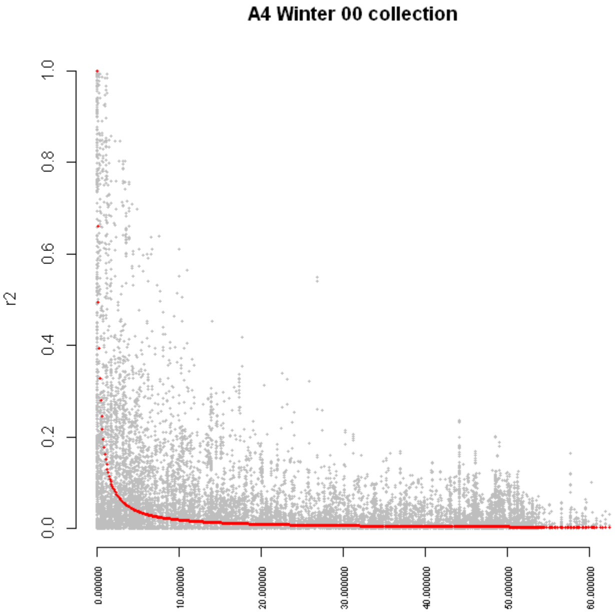
A4 Winter 00 collection
r2
0.0
0.2
0.4
0.6
0.8
1.0
0.000000
10.000000
20.000000
30.000000
40.000000
50.000000
60.000000

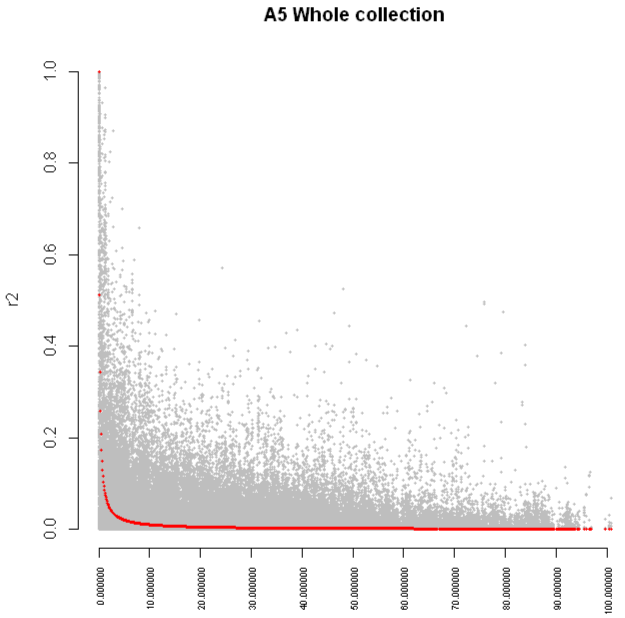
A5 Whole collection
r2
0.0
0.2
0.4
0.6
0.8
1.0
0.000000
10.000000
20.000000
30.000000
40.000000
50.000000
60.000000
70.000000
80.000000
90.000000
100.000000

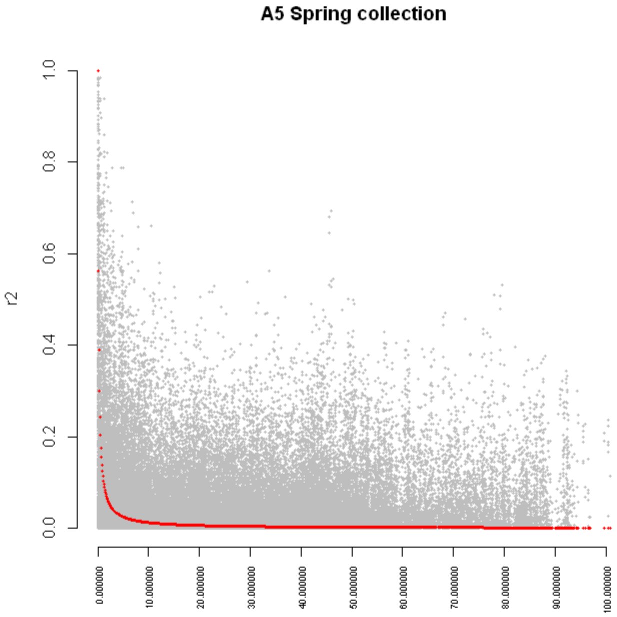
A5 Spring collection
r2
0.0
0.2
0.4
0.6
0.8
1.0
0.000000
10.000000
20.000000
30.000000
40.000000
50.000000
60.000000
70.000000
80.000000
90.000000
100.000000

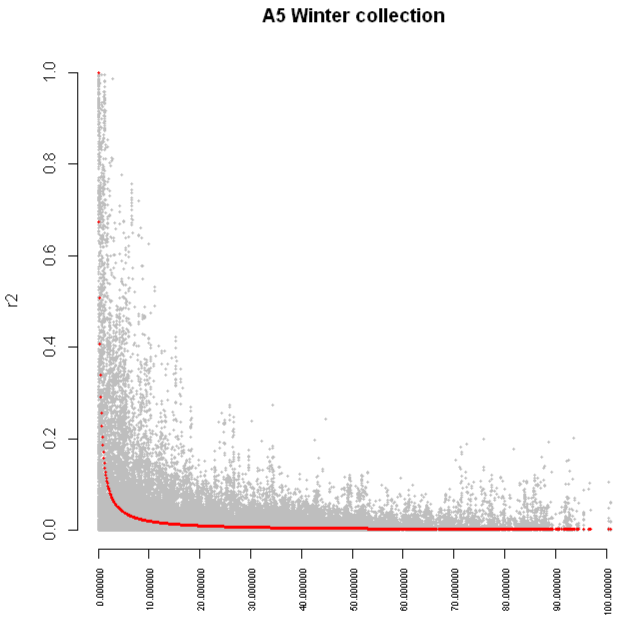
A5 Winter collection
r2
0.0
0.2
0.4
0.6
0.8
1.0
0.000000
10.000000
20.000000
30.000000
40.000000
50.000000
60.000000
70.000000
80.000000
90.000000
100.000000

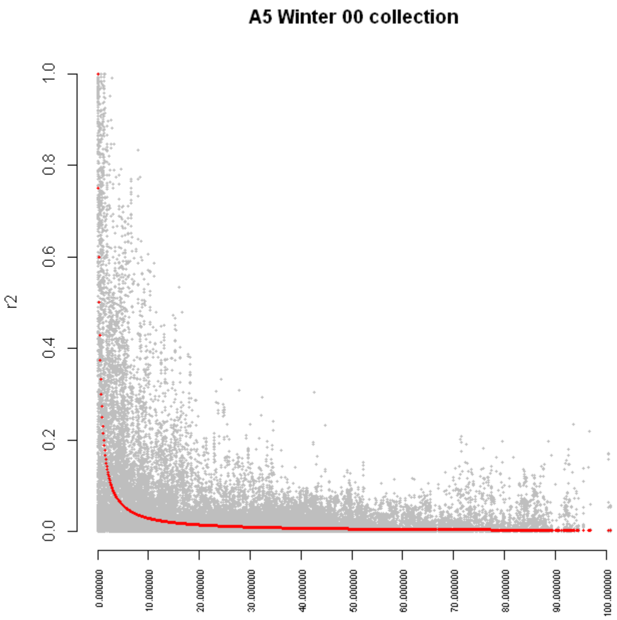
A5 Winter 00 collection
r2
0.0
0.2
0.4
0.6
0.8
1.0
0.000000
10.000000
20.000000
30.000000
40.000000
50.000000
60.000000
70.000000
80.000000
90.000000
100.000000

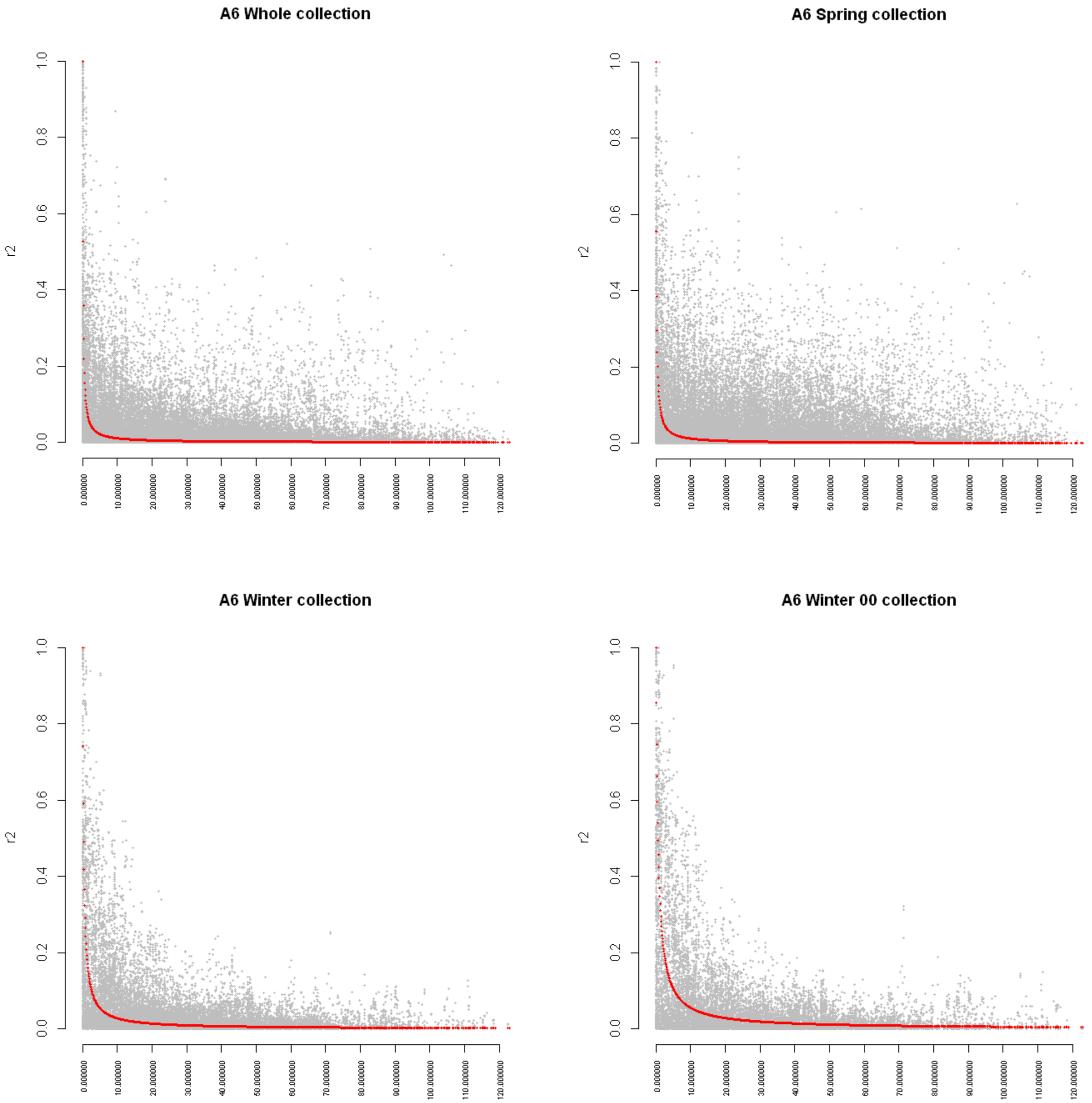
A6 Whole collection
A6 Spring collection
A6 Winter collection
A6 Winter 00 collection
r2
0.0
0.2
0.4
0.6
0.8
1.0
0.000000
10.000000
20.000000
30.000000
40.000000
50.000000
60.000000
70.000000
80.000000
90.000000
100.000000
110.000000
120.000000

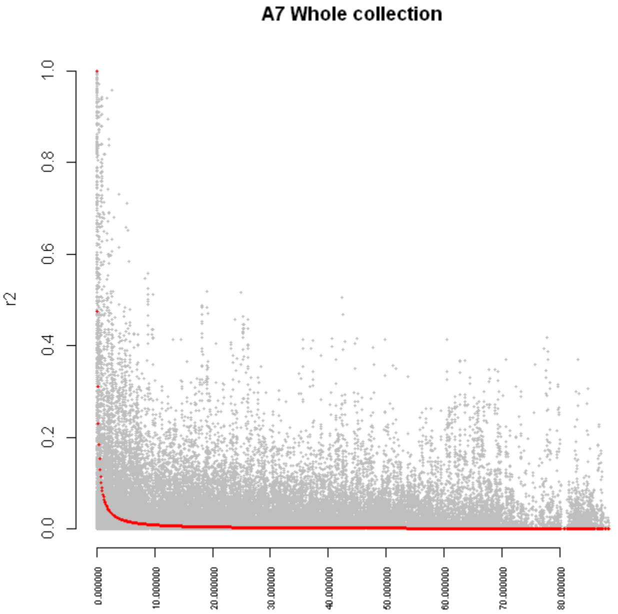
A7 Whole collection
r2
0.0
0.2
0.4
0.6
0.8
1.0
0.000000
10.000000
20.000000
30.000000
40.000000
50.000000
60.000000
70.000000
80.000000

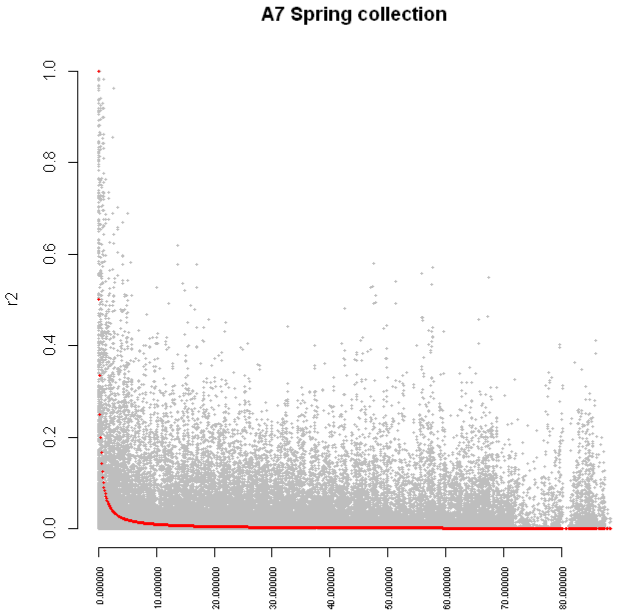
A7 Spring collection
r2
0.0
0.2
0.4
0.6
0.8
1.0
0.000000
10.000000
20.000000
30.000000
40.000000
50.000000
60.000000
70.000000
80.000000

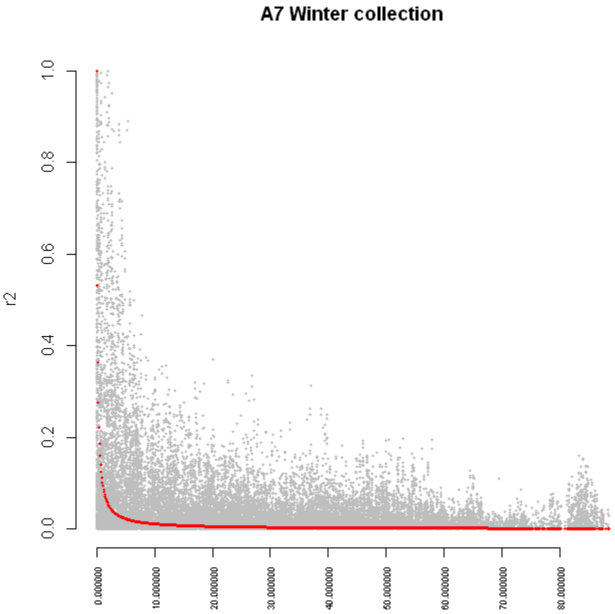
A7 Winter collection
r2
0.0
0.2
0.4
0.6
0.8
1.0
0.000000
10.000000
20.000000
30.000000
40.000000
50.000000
60.000000
70.000000
80.000000

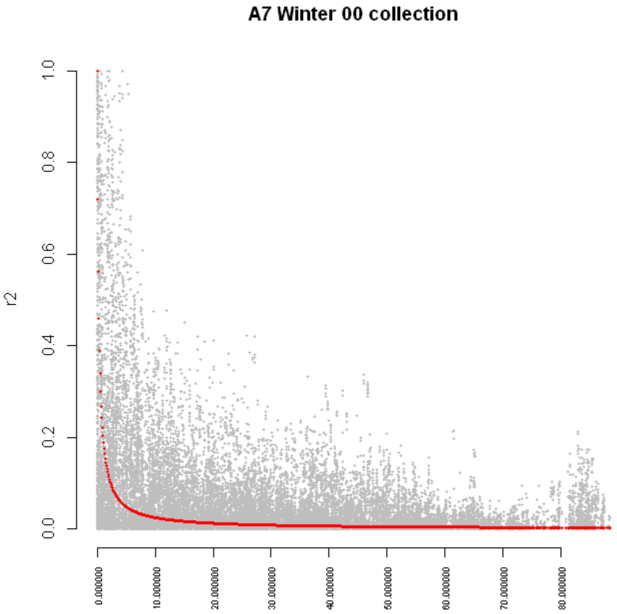
A7 Winter 00 collection
r2
0.0
0.2
0.4
0.6
0.8
1.0
0.000000
10.000000
20.000000
30.000000
40.000000
50.000000
60.000000
70.000000
80.000000

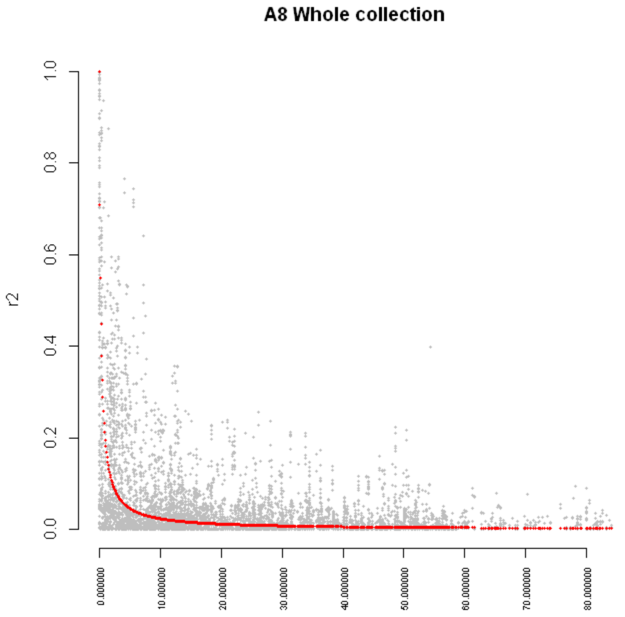
A8 Whole collection
r2
0.0
0.2
0.4
0.6
0.8
1.0
0.000000
10.000000
20.000000
30.000000
40.000000
50.000000
60.000000
70.000000
80.000000

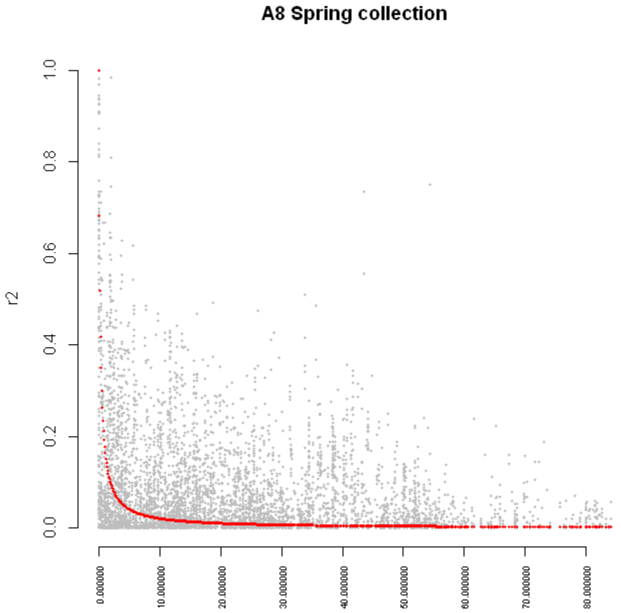
A8 Spring collection
r2
0.0
0.2
0.4
0.6
0.8
1.0
0.000000
10.000000
20.000000
30.000000
40.000000
50.000000
60.000000
70.000000
80.000000

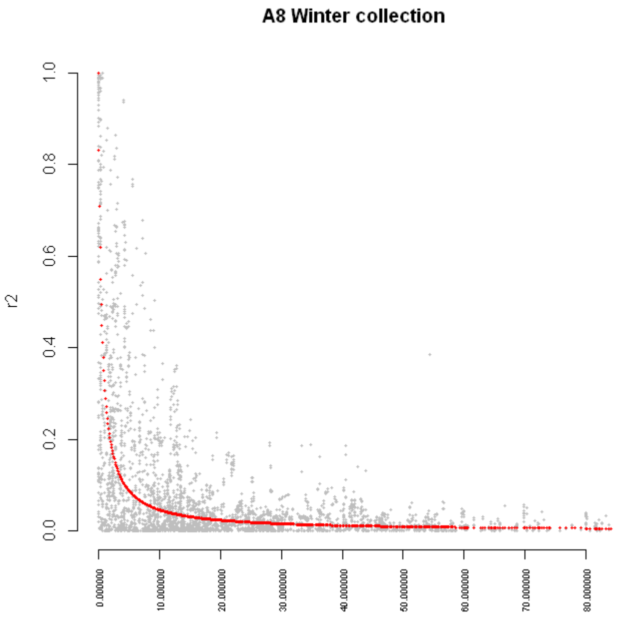
A8 Winter collection
r2
0.0
0.2
0.4
0.6
0.8
1.0
0.000000
10.000000
20.000000
30.000000
40.000000
50.000000
60.000000
70.000000
80.000000

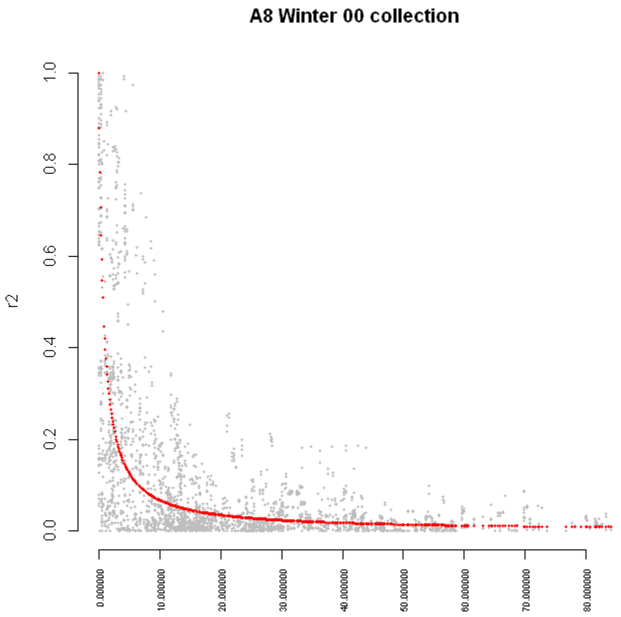
A8 Winter 00 collection
r2
0.0
0.2
0.4
0.6
0.8
1.0
0.000000
10.000000
20.000000
30.000000
40.000000
50.000000
60.000000
70.000000
80.000000

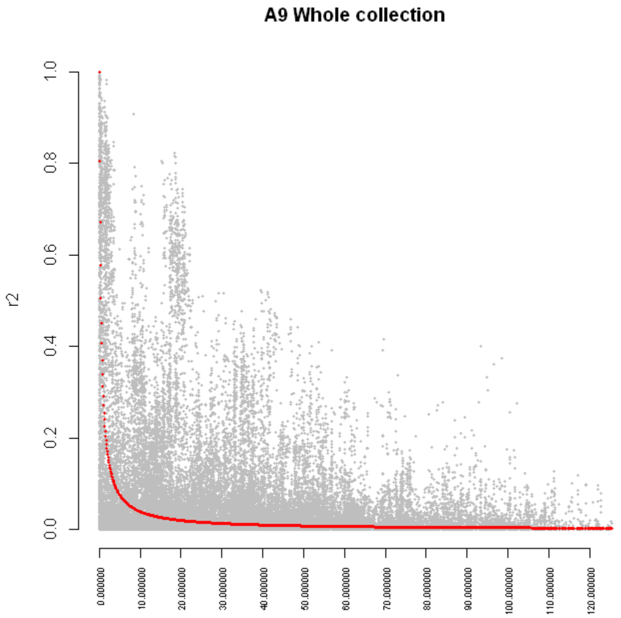
A9 Whole collection
r2
0.0
0.2
0.4
0.6
0.8
1.0
0.000000
10.000000
20.000000
30.000000
40.000000
50.000000
60.000000
70.000000
80.000000
90.000000
100.000000
110.000000
120.000000

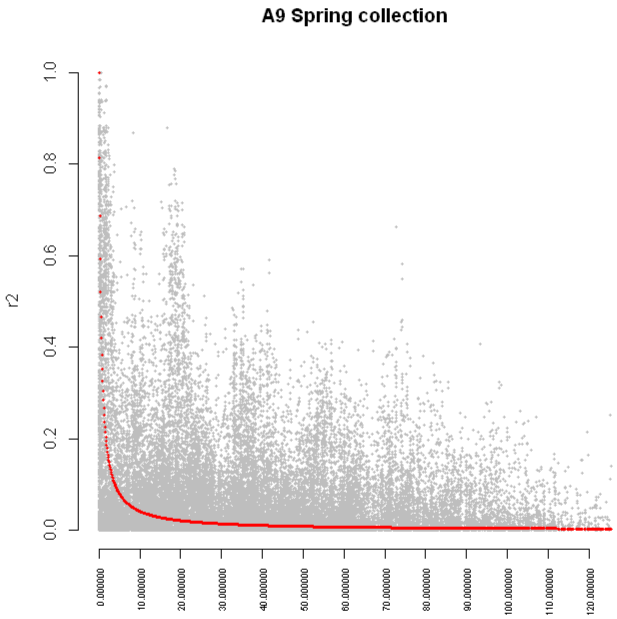
A9 Spring collection
r2
0.0
0.2
0.4
0.6
0.8
1.0
0.000000
10.000000
20.000000
30.000000
40.000000
50.000000
60.000000
70.000000
80.000000
90.000000
100.000000
110.000000
120.000000

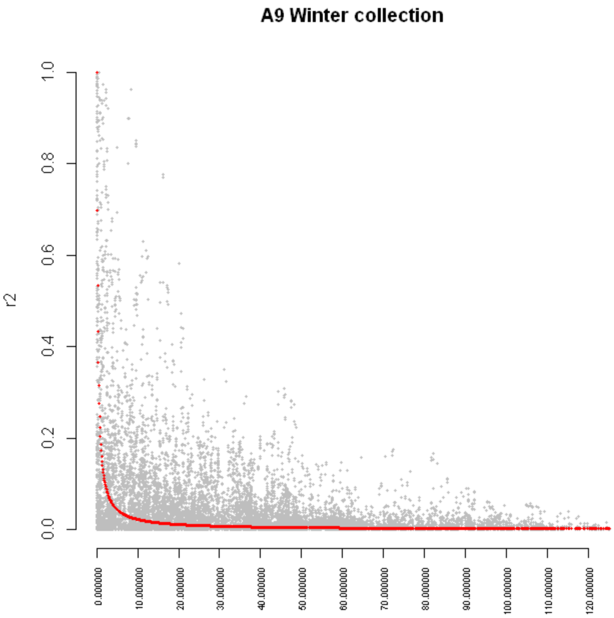
A9 Winter collection
r2
0.0
0.2
0.4
0.6
0.8
1.0
0.000000
10.000000
20.000000
30.000000
40.000000
50.000000
60.000000
70.000000
80.000000
90.000000
100.000000
110.000000
120.000000

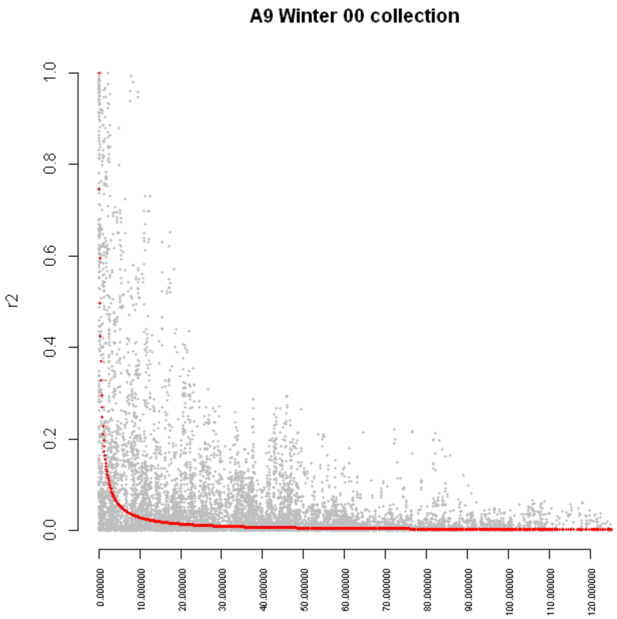
A9 Winter 00 collection
r2
0.0
0.2
0.4
0.6
0.8
1.0
0.000000
10.000000
20.000000
30.000000
40.000000
50.000000
60.000000
70.000000
80.000000
90.000000
100.000000
110.000000
120.000000

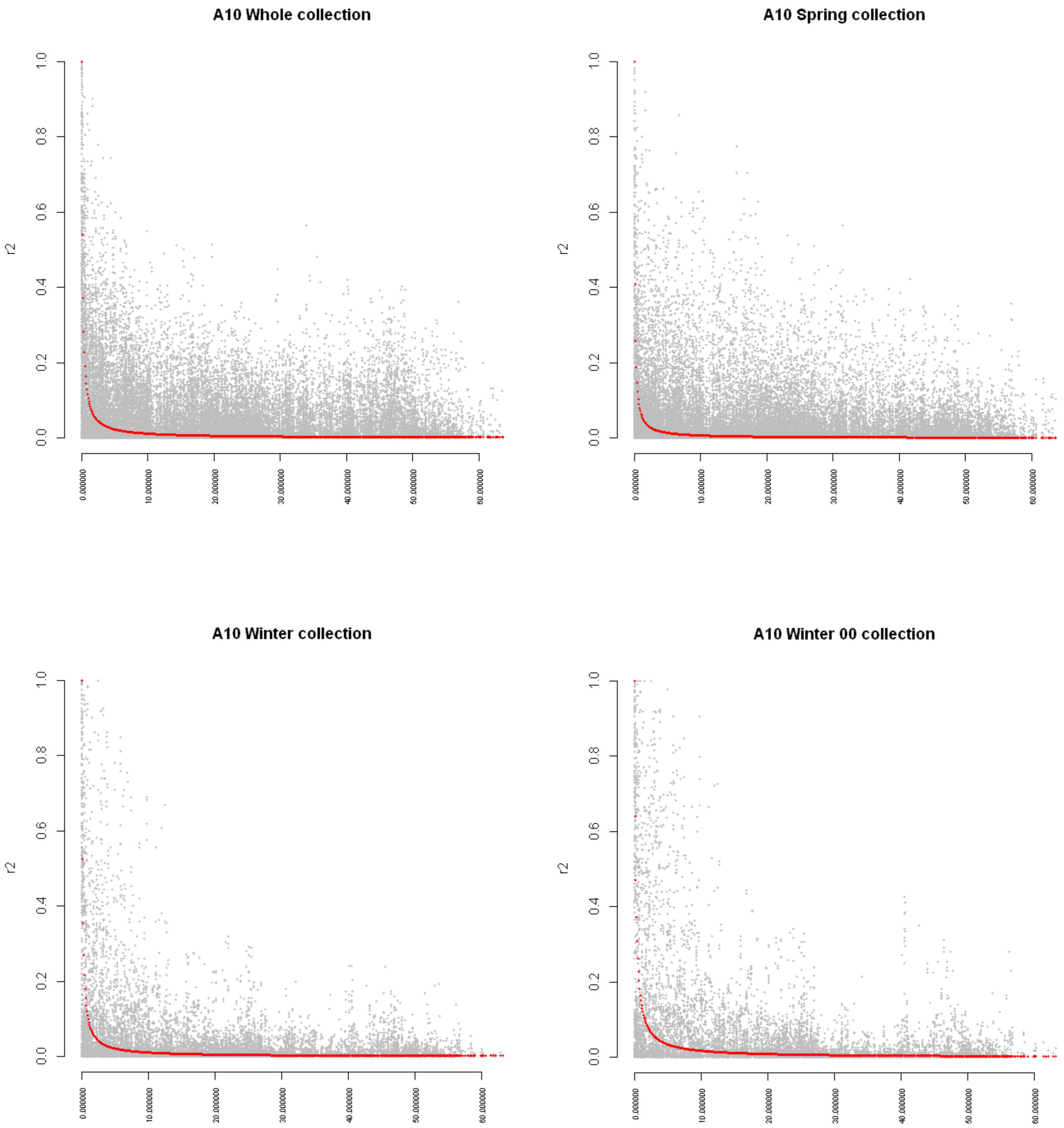
A10 Whole collection
A10 Spring collection
A10 Winter collection
A10 Winter 00 collection
r2
0.0
0.2
0.4
0.6
0.8
1.0
0.000000
10.000000
20.000000
30.000000
40.000000
50.000000
60.000000

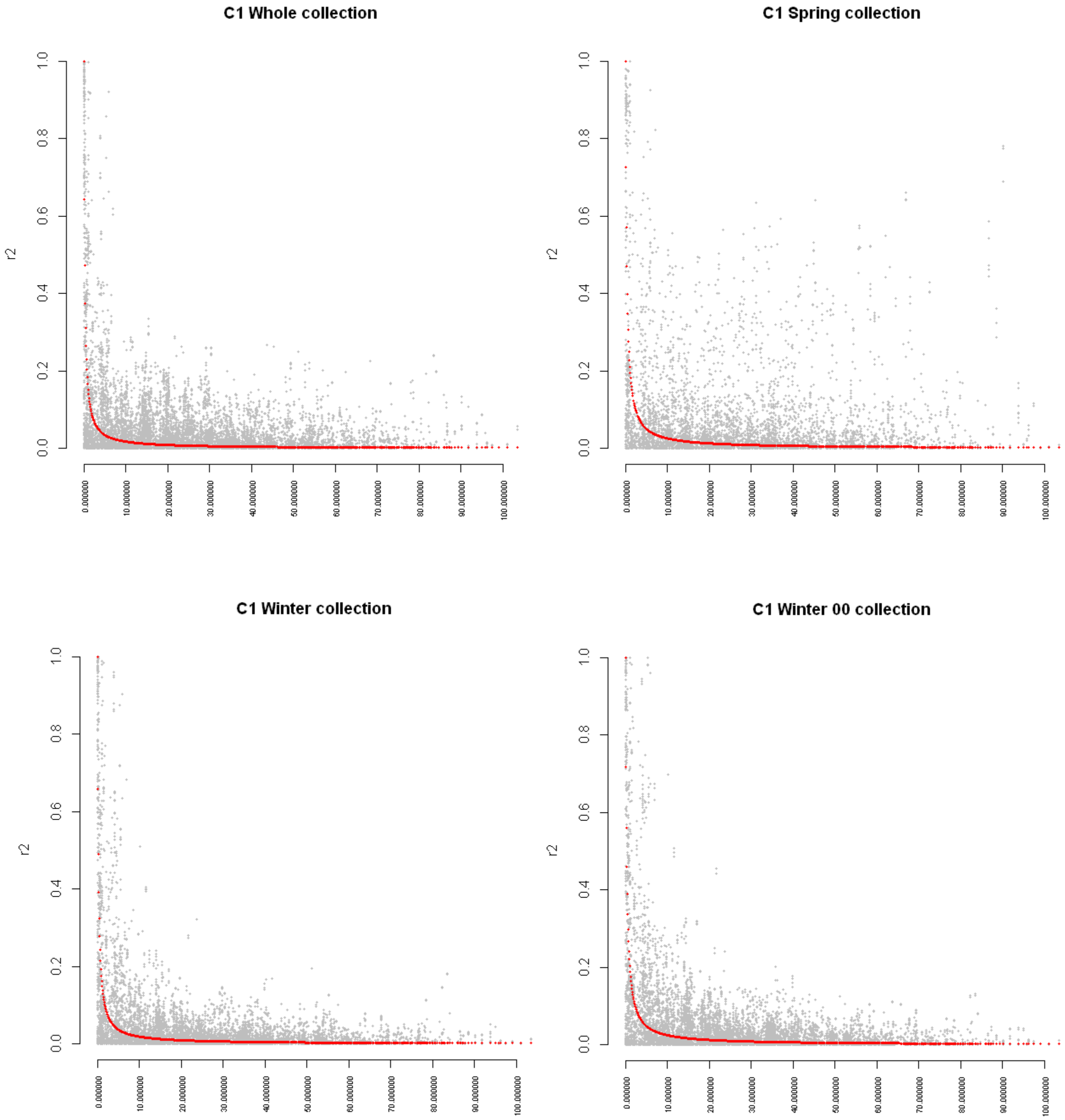
C1 Whole collection
C1 Spring collection
C1 Winter collection
C1 Winter 00 collection
r2
0.0
0.2
0.4
0.6
0.8
1.0
0.000000
10.000000
20.000000
30.000000
40.000000
50.000000
60.000000
70.000000
80.000000
90.000000
100.000000

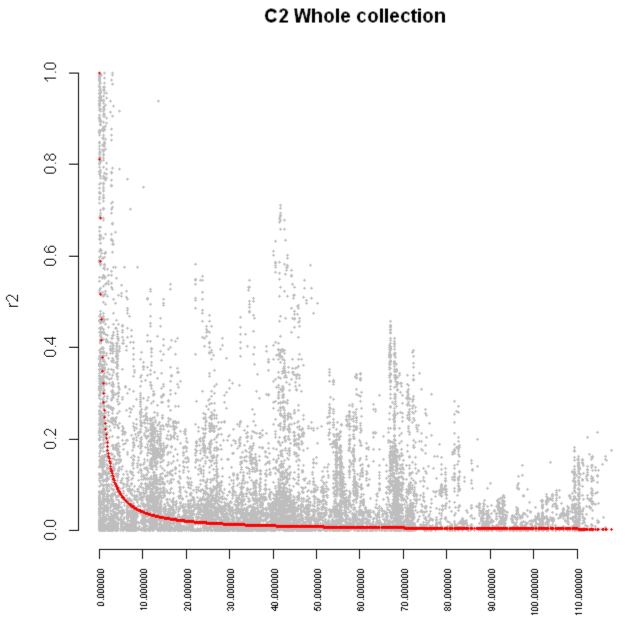
C2 Whole collection
r2
1.0
0.8
0.6
0.4
0.2
0.0
0.000000
10.000000
20.000000
30.000000
40.000000
50.000000
60.000000
70.000000
80.000000
90.000000
100.000000
110.000000

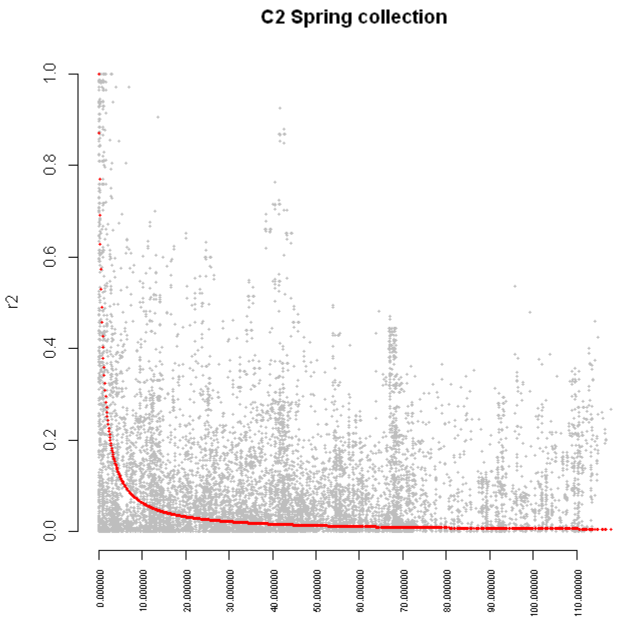
C2 Spring collection
r2
1.0
0.8
0.6
0.4
0.2
0.0
0.000000
10.000000
20.000000
30.000000
40.000000
50.000000
60.000000
70.000000
80.000000
90.000000
100.000000
110.000000

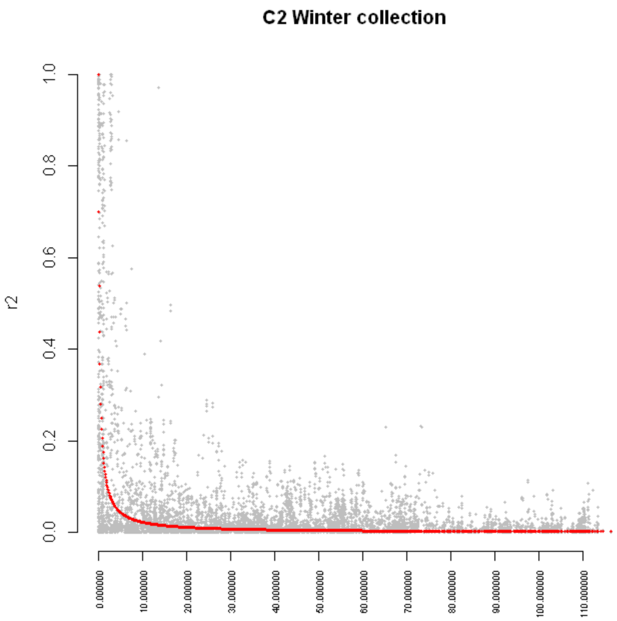
C2 Winter collection
r2
1.0
0.8
0.6
0.4
0.2
0.0
0.000000
10.000000
20.000000
30.000000
40.000000
50.000000
60.000000
70.000000
80.000000
90.000000
100.000000
110.000000

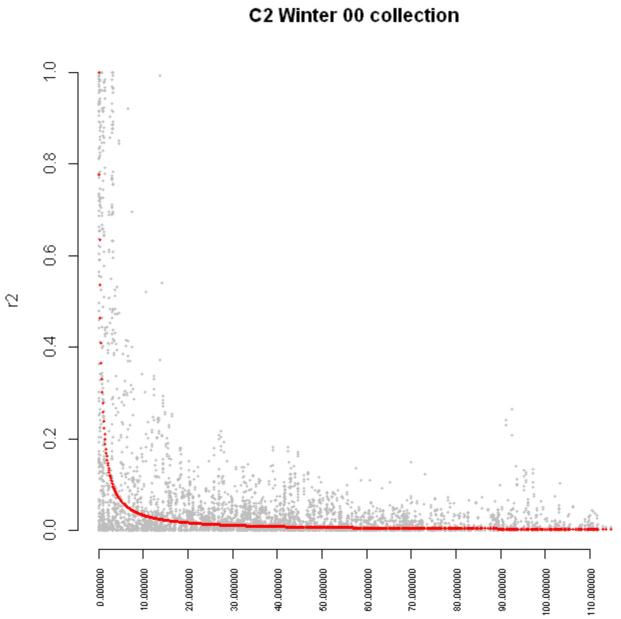
C2 Winter 00 collection
r2
1.0
0.8
0.6
0.4
0.2
0.0
0.000000
10.000000
20.000000
30.000000
40.000000
50.000000
60.000000
70.000000
80.000000
90.000000
100.000000
110.000000

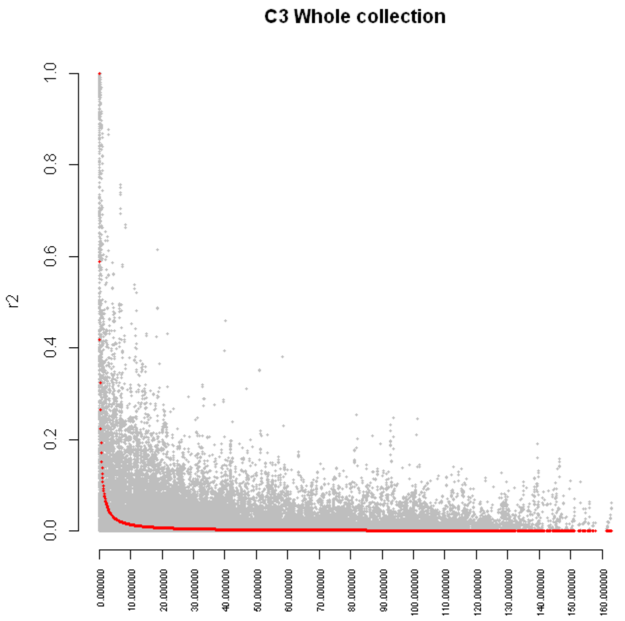
C3 Whole collection
r2
0.0
0.2
0.4
0.6
0.8
1.0
0.000000
10.000000
20.000000
30.000000
40.000000
50.000000
60.000000
70.000000
80.000000
90.000000
100.000000
110.000000
120.000000
130.000000
140.000000
150.000000
160.000000

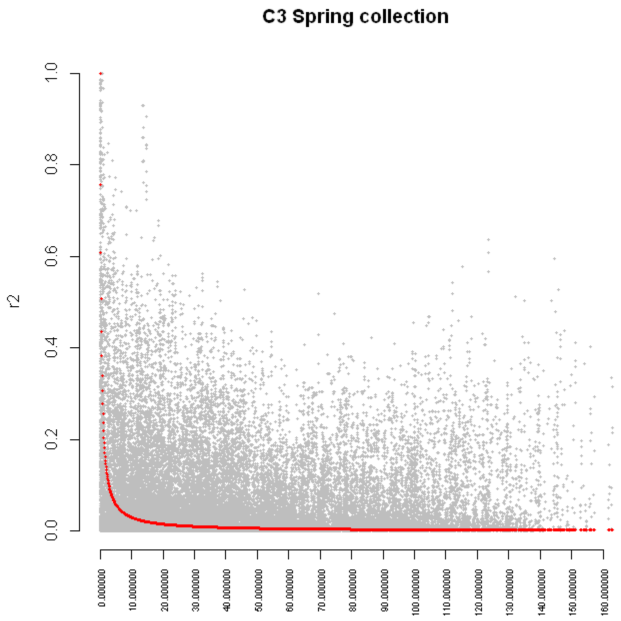
C3 Spring collection
r2
0.0
0.2
0.4
0.6
0.8
1.0
0.000000
10.000000
20.000000
30.000000
40.000000
50.000000
60.000000
70.000000
80.000000
90.000000
100.000000
110.000000
120.000000
130.000000
140.000000
150.000000
160.000000

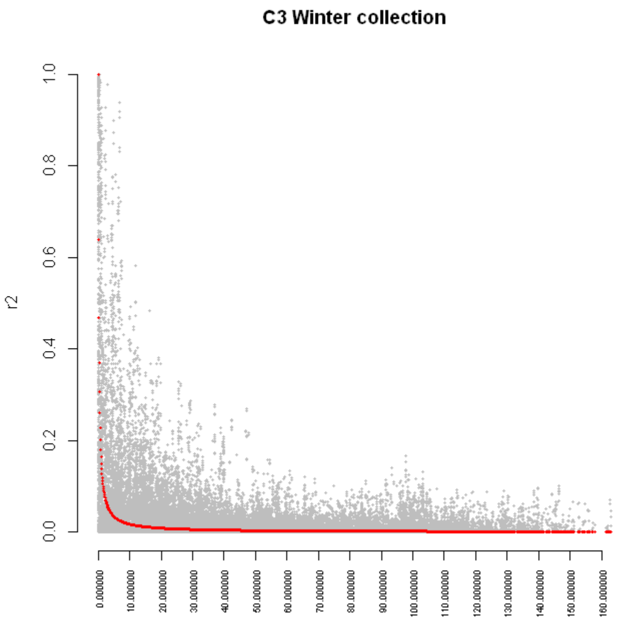
C3 Winter collection
r2
0.0
0.2
0.4
0.6
0.8
1.0
0.000000
10.000000
20.000000
30.000000
40.000000
50.000000
60.000000
70.000000
80.000000
90.000000
100.000000
110.000000
120.000000
130.000000
140.000000
150.000000
160.000000

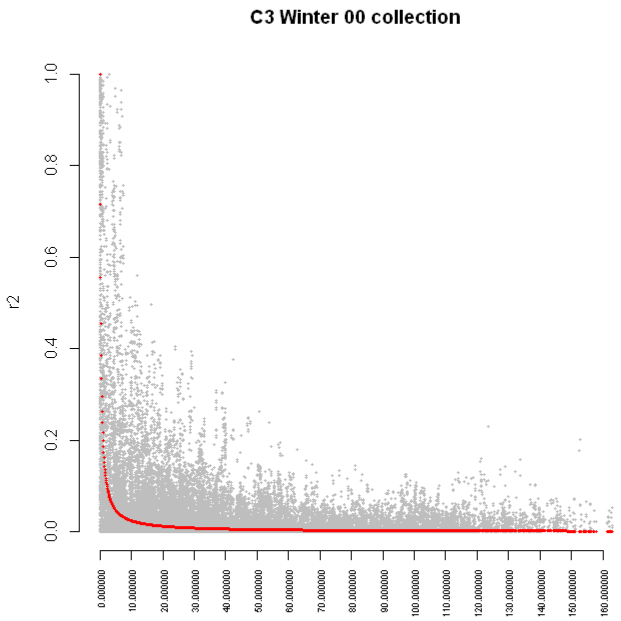
C3 Winter 00 collection
r2
0.0
0.2
0.4
0.6
0.8
1.0
0.000000
10.000000
20.000000
30.000000
40.000000
50.000000
60.000000
70.000000
80.000000
90.000000
100.000000
110.000000
120.000000
130.000000
140.000000
150.000000
160.000000

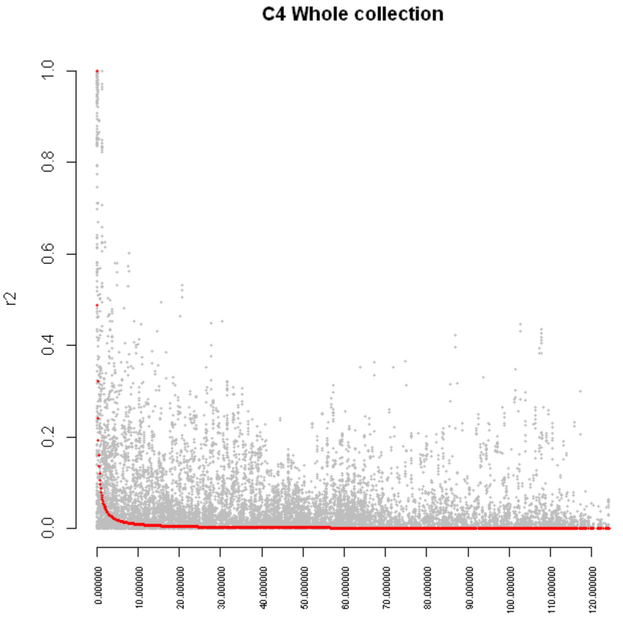
C4 Whole collection
r2
0.0
0.2
0.4
0.6
0.8
1.0
0.000000
10.000000
20.000000
30.000000
40.000000
50.000000
60.000000
70.000000
80.000000
90.000000
100.000000
110.000000
120.000000

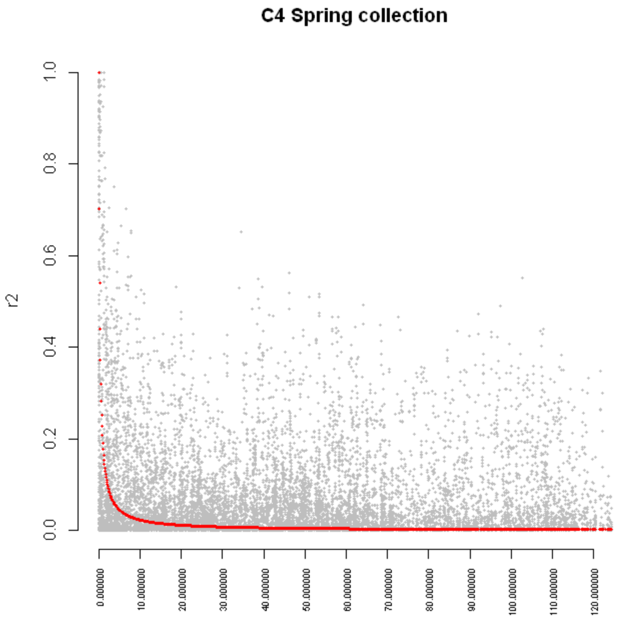
C4 Spring collection
r2
0.0
0.2
0.4
0.6
0.8
1.0
0.000000
10.000000
20.000000
30.000000
40.000000
50.000000
60.000000
70.000000
80.000000
90.000000
100.000000
110.000000
120.000000

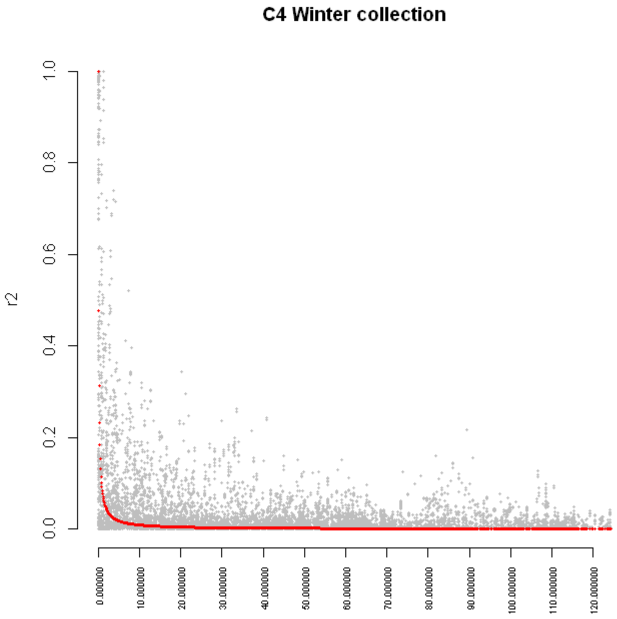
C4 Winter collection
r2
0.0
0.2
0.4
0.6
0.8
1.0
0.000000
10.000000
20.000000
30.000000
40.000000
50.000000
60.000000
70.000000
80.000000
90.000000
100.000000
110.000000
120.000000

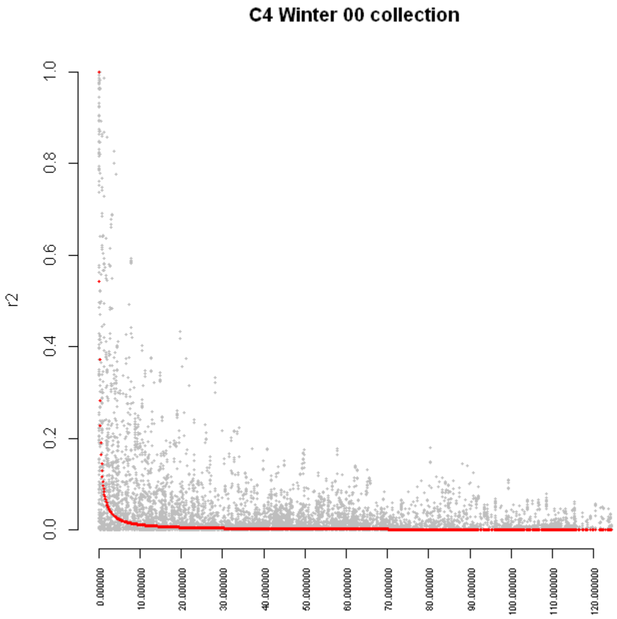
C4 Winter 00 collection
r2
0.0
0.2
0.4
0.6
0.8
1.0
0.000000
10.000000
20.000000
30.000000
40.000000
50.000000
60.000000
70.000000
80.000000
90.000000
100.000000
110.000000
120.000000

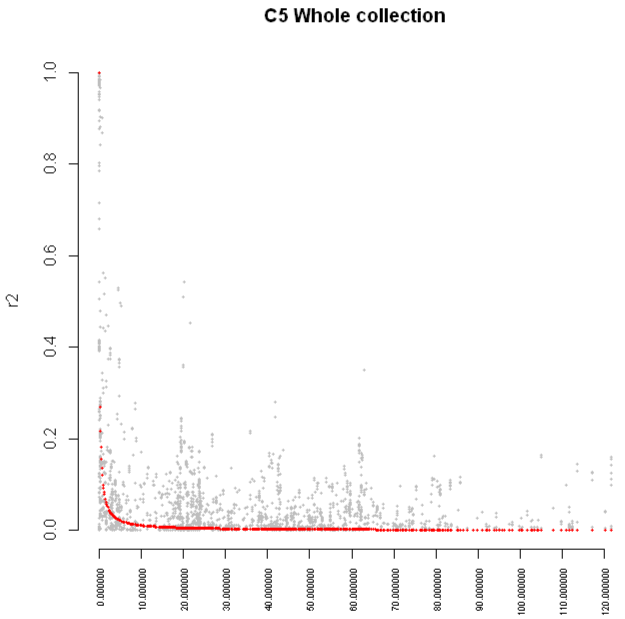
C5 Whole collection
r2
0.0
0.2
0.4
0.6
0.8
1.0
0.000000
10.000000
20.000000
30.000000
40.000000
50.000000
60.000000
70.000000
80.000000
90.000000
100.000000
110.000000
120.000000

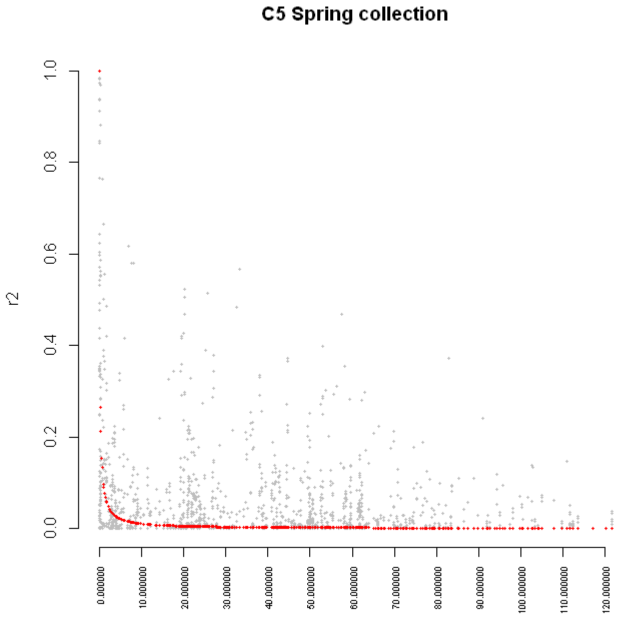
C5 Spring collection
r2
0.0
0.2
0.4
0.6
0.8
1.0
0.000000
10.000000
20.000000
30.000000
40.000000
50.000000
60.000000
70.000000
80.000000
90.000000
100.000000
110.000000
120.000000

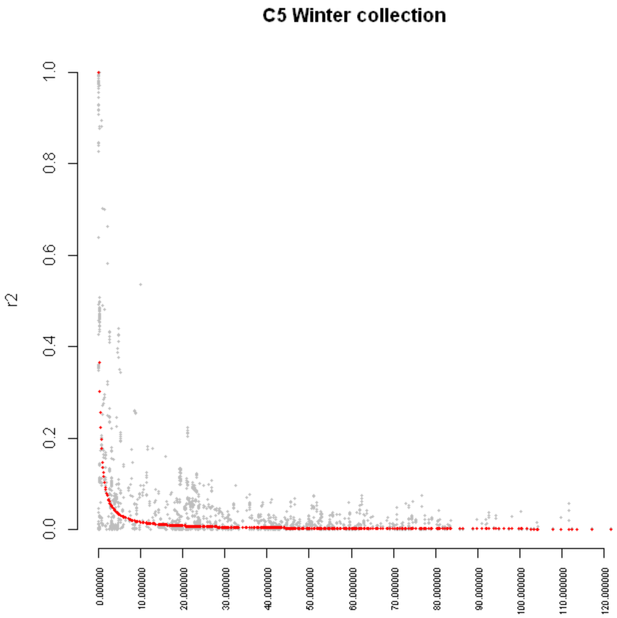
C5 Winter collection
r2
0.0
0.2
0.4
0.6
0.8
1.0
0.000000
10.000000
20.000000
30.000000
40.000000
50.000000
60.000000
70.000000
80.000000
90.000000
100.000000
110.000000
120.000000

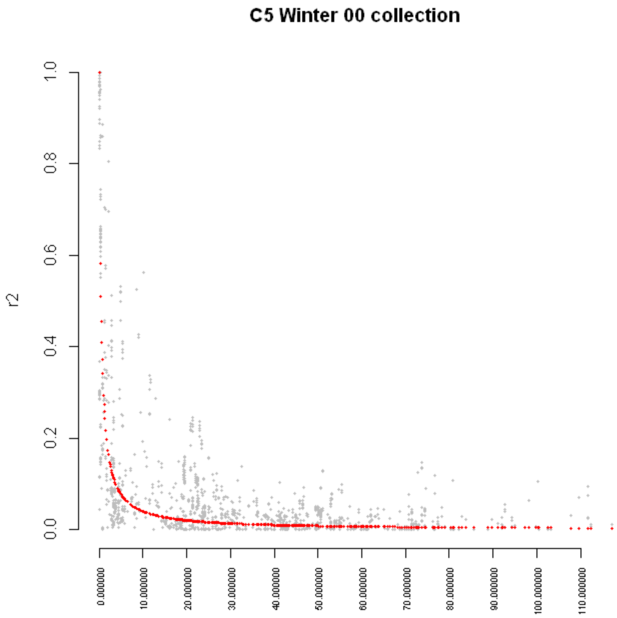
C5 Winter 00 collection
r2
0.0
0.2
0.4
0.6
0.8
1.0
0.000000
10.000000
20.000000
30.000000
40.000000
50.000000
60.000000
70.000000
80.000000
90.000000
100.000000
110.000000

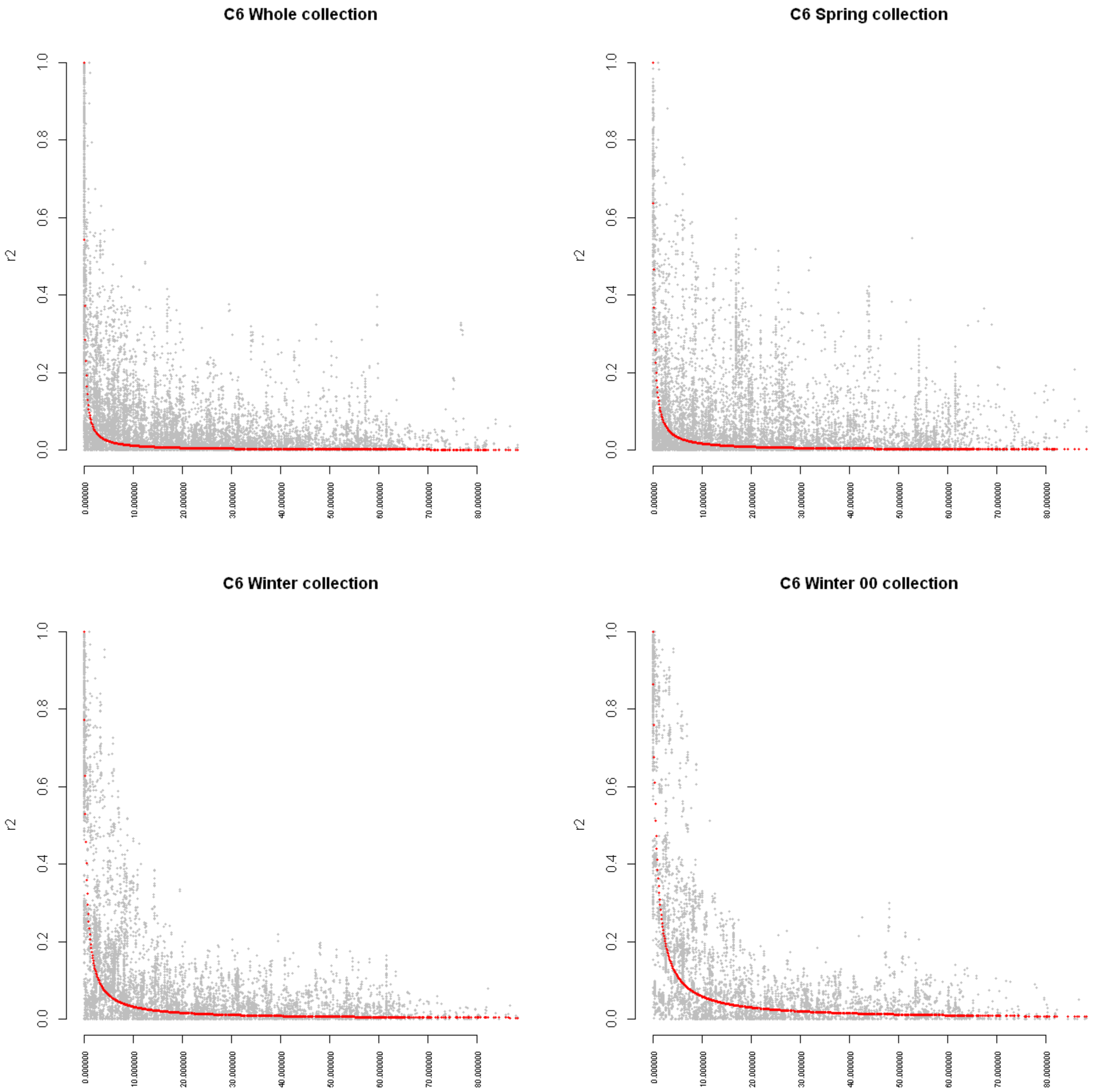
C6 Whole collection
C6 Spring collection
C6 Winter collection
C6 Winter 00 collection
r2
0.0
0.2
0.4
0.6
0.8
1.0
0.000000
10.000000
20.000000
30.000000
40.000000
50.000000
60.000000
70.000000
80.000000

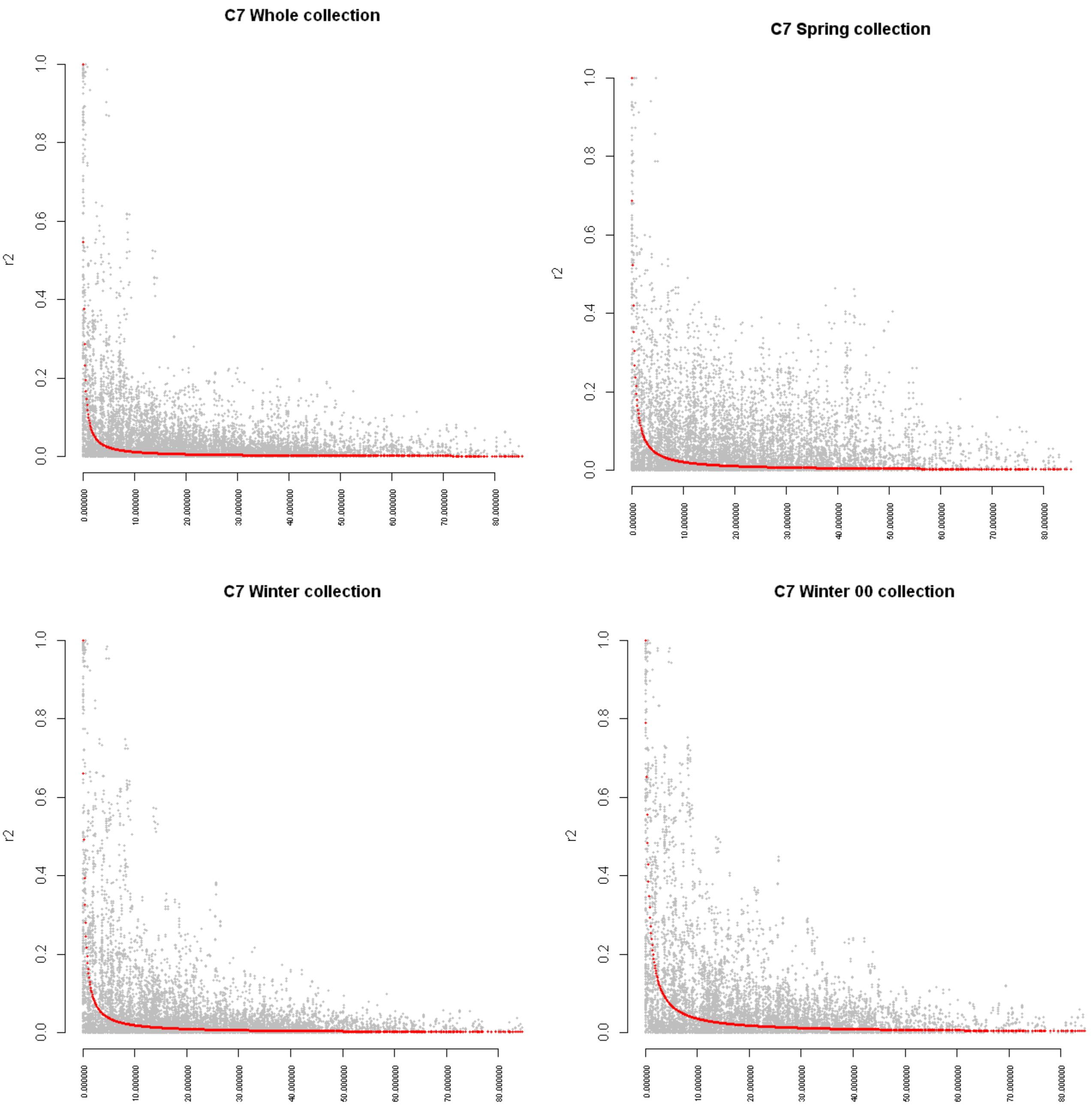
C7 Whole collection
C7 Spring collection
C7 Winter collection
C7 Winter 00 collection
r2
0.0
0.2
0.4
0.6
0.8
1.0
0.000000
10.000000
20.000000
30.000000
40.000000
50.000000
60.000000
70.000000
80.000000

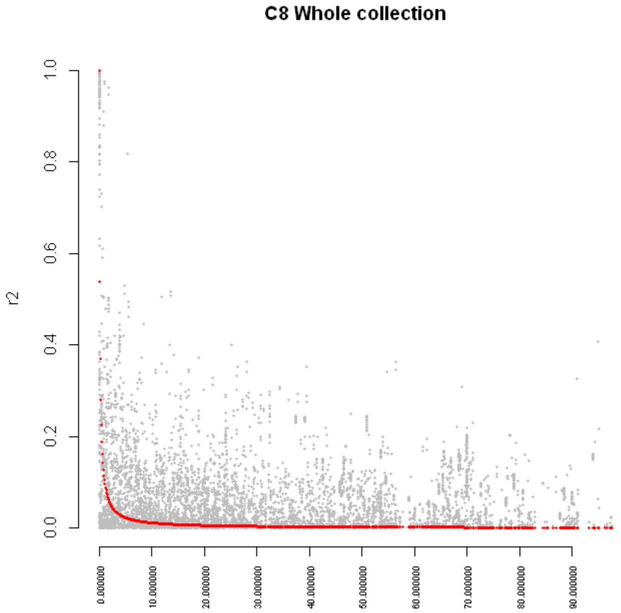

C8 Whole collection
r2
1.0
0.8
0.6
0.4
0.2
0.0
0.000000
10.000000
20.000000
30.000000
40.000000
50.000000
60.000000
70.000000
80.000000
90.000000


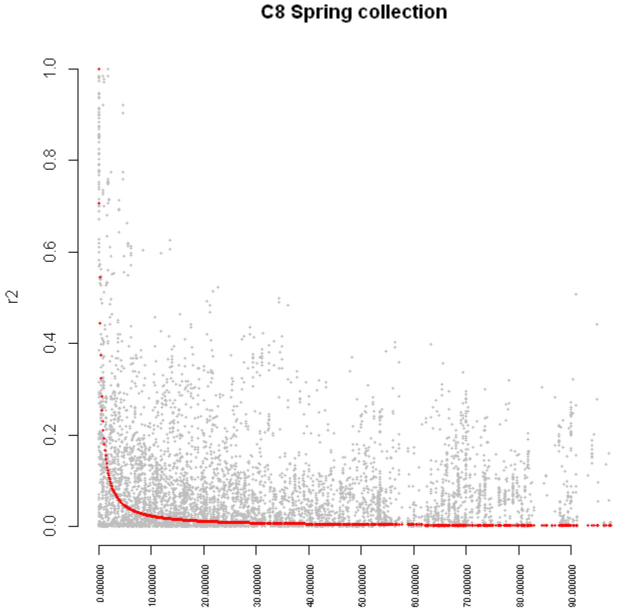

C8 Spring collection
r2
1.0
0.8
0.6
0.4
0.2
0.0
0.000000
10.000000
20.000000
30.000000
40.000000
50.000000
60.000000
70.000000
80.000000
90.000000


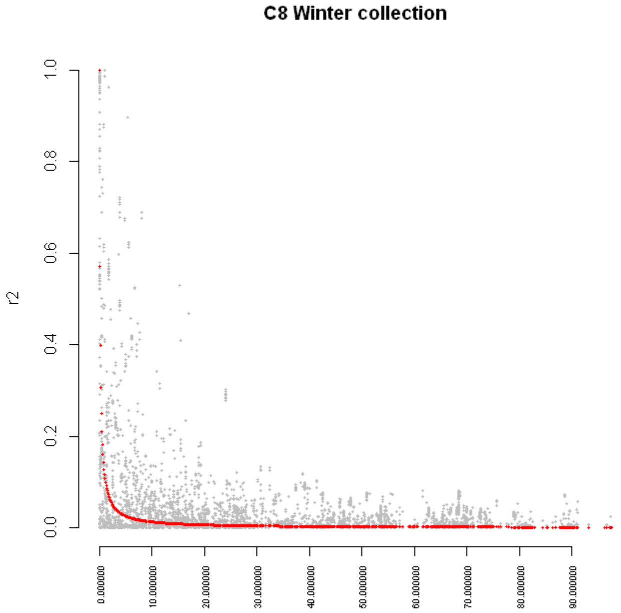

C8 Winter collection
r2
1.0
0.8
0.6
0.4
0.2
0.0
0.000000
10.000000
20.000000
30.000000
40.000000
50.000000
60.000000
70.000000
80.000000
90.000000


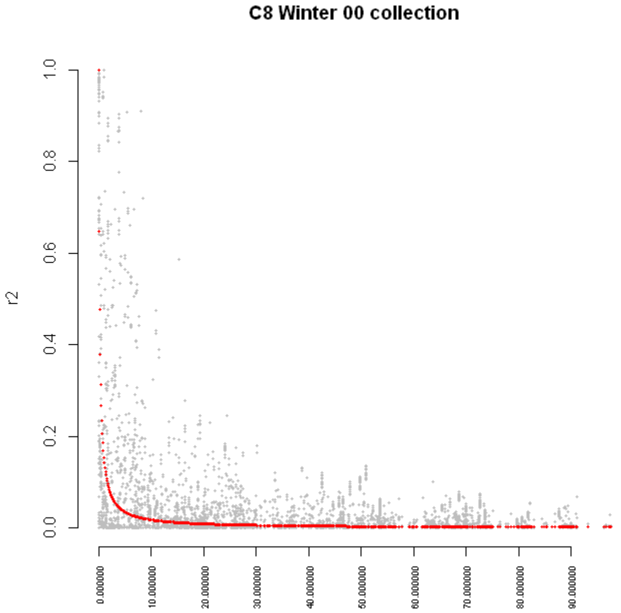

C8 Winter 00 collection
r2
1.0
0.8
0.6
0.4
0.2
0.0
0.000000
10.000000
20.000000
30.000000
40.000000
50.000000
60.000000
70.000000
80.000000
90.000000

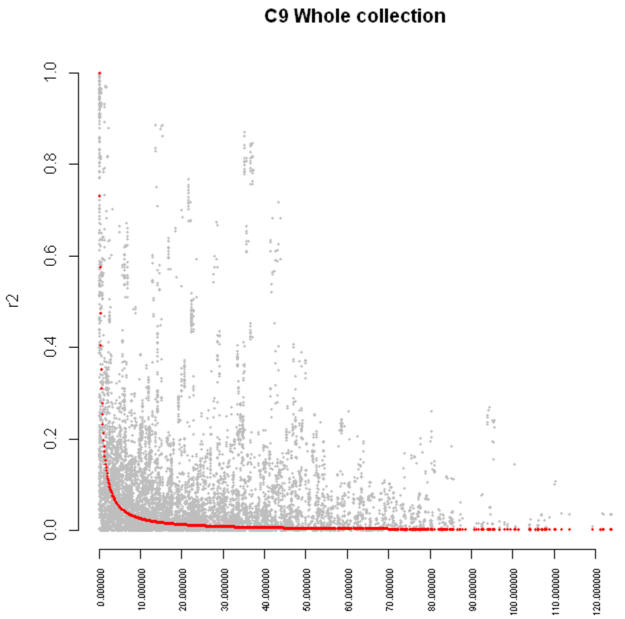
C9 Whole collection
r2
0.0
0.2
0.4
0.6
0.8
1.0
0.000000
10.000000
20.000000
30.000000
40.000000
50.000000
60.000000
70.000000
80.000000
90.000000
100.000000
110.000000
120.000000

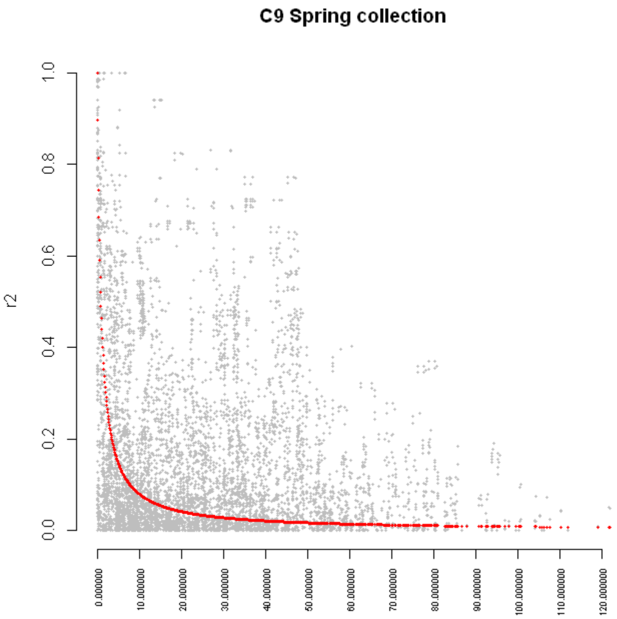
C9 Spring collection
r2
0.0
0.2
0.4
0.6
0.8
1.0
0.000000
10.000000
20.000000
30.000000
40.000000
50.000000
60.000000
70.000000
80.000000
90.000000
100.000000
110.000000
120.000000

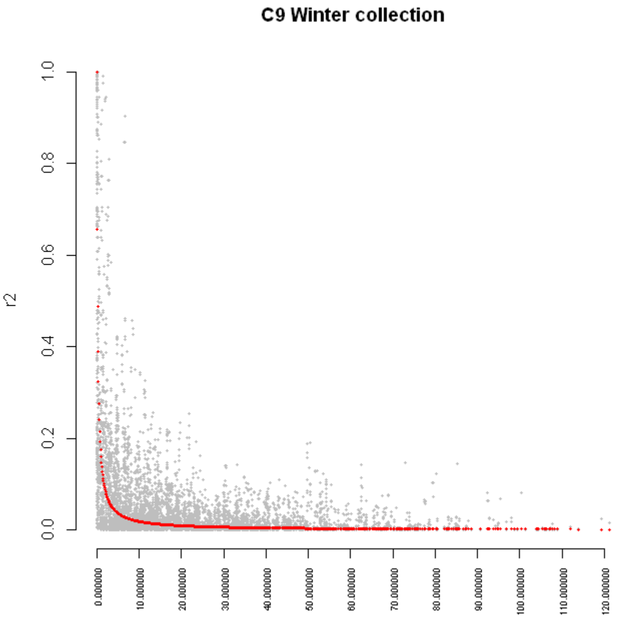
C9 Winter collection
r2
0.0
0.2
0.4
0.6
0.8
1.0
0.000000
10.000000
20.000000
30.000000
40.000000
50.000000
60.000000
70.000000
80.000000
90.000000
100.000000
110.000000
120.000000

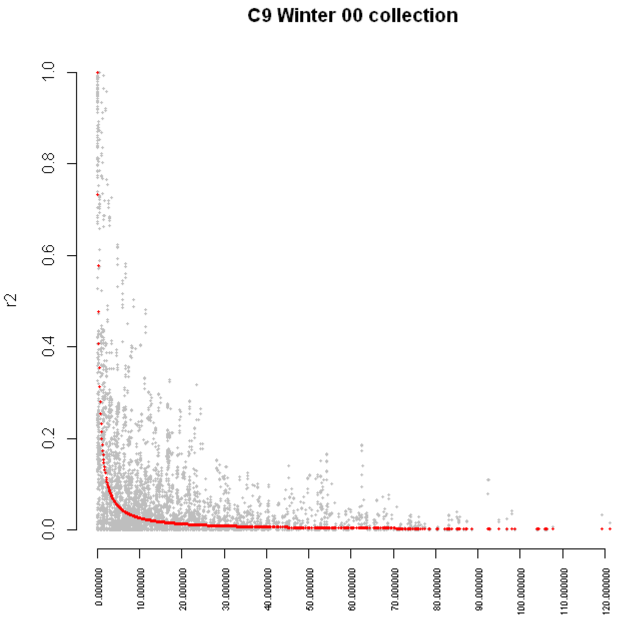
C9 Winter 00 collection
r2
0.0
0.2
0.4
0.6
0.8
1.0
0.000000
10.000000
20.000000
30.000000
40.000000
50.000000
60.000000
70.000000
80.000000
90.000000
100.000000
110.000000
120.000000
