# Supplementary material for: High-density SNP-based genetic map development and linkage disequilibrium assessment in Brassica napus L
Source: BMC Genomics. 2013 Feb 22;14:120. doi: 10.1186/1471-2164-14-120 (PMC3600037; doi:10.1186/1471-2164-14-120)

Supplementary figure 5: Linkage disequilibrium heatmaps for each linkage group in the whole, spring (SOSR), winter (WOSR) and « 00 » WOSR collections. The putative position of the centromeres is indicated by a black arrow.

Whole collection

A1

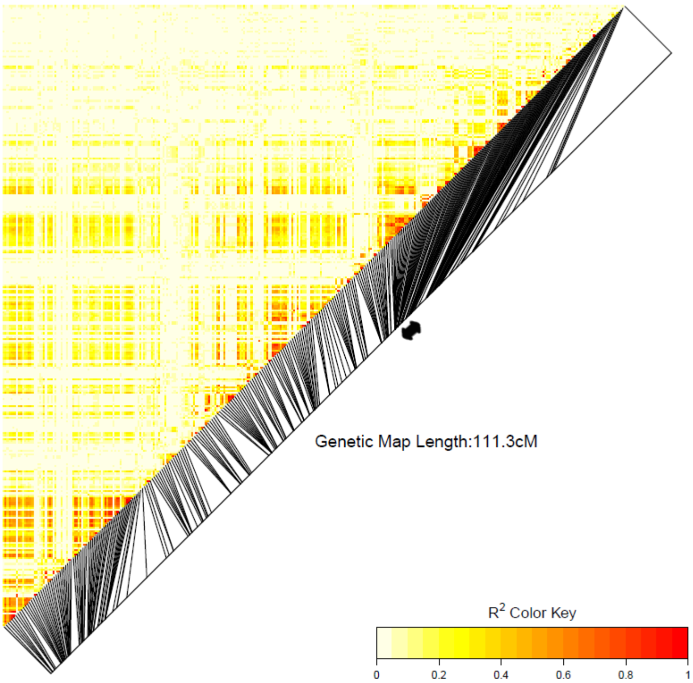

SOSR collection

A1

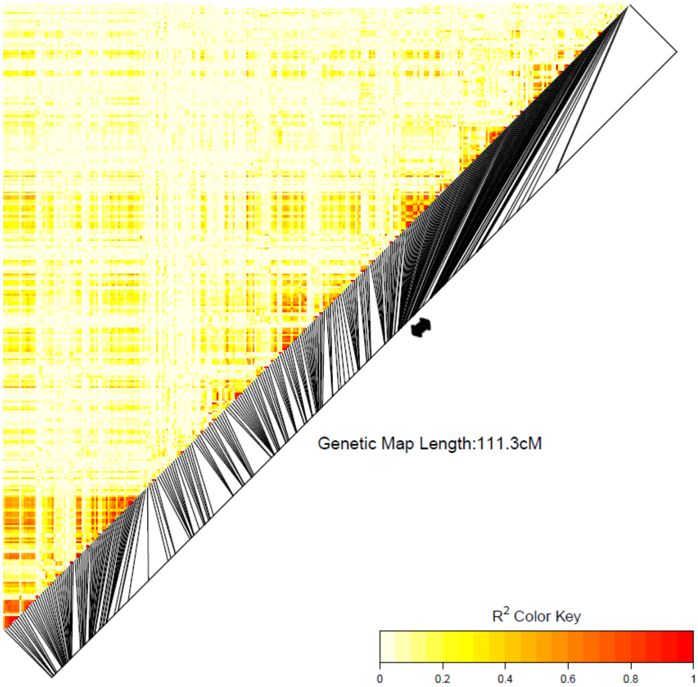

WOSR collection

A1

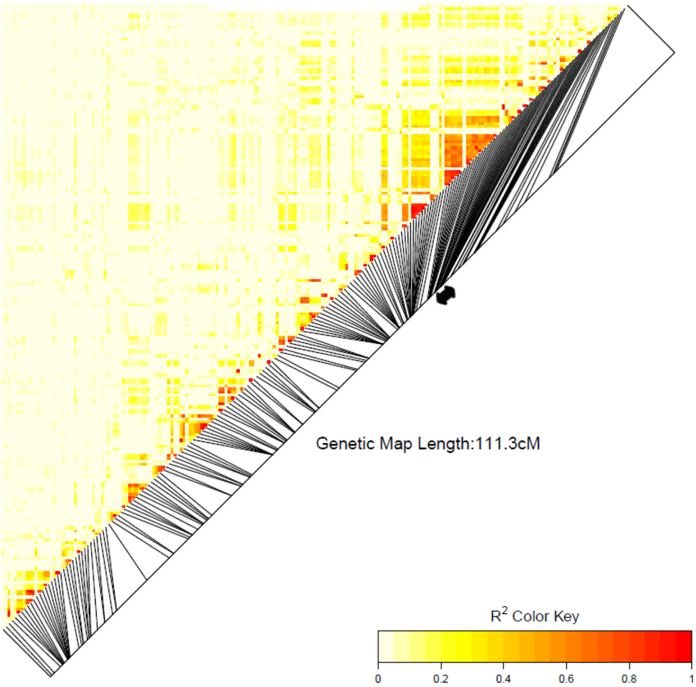

« 00 » WOSR collection

A1

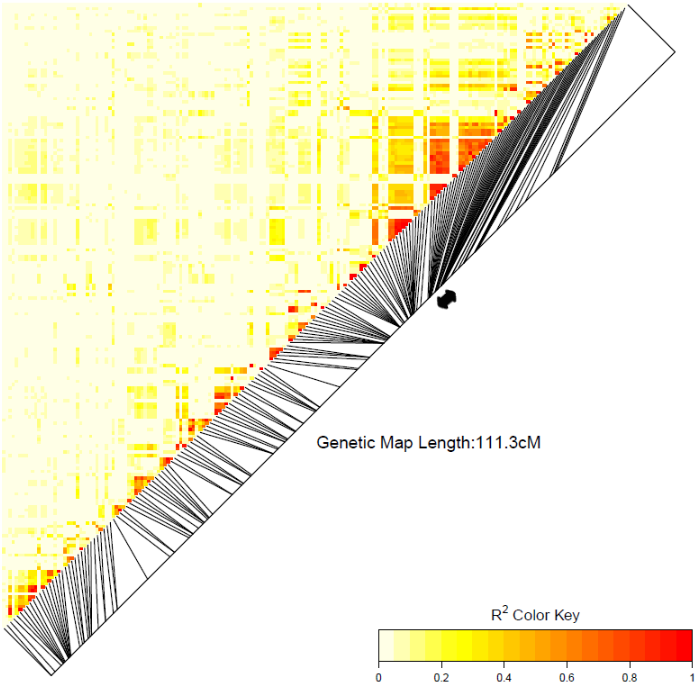

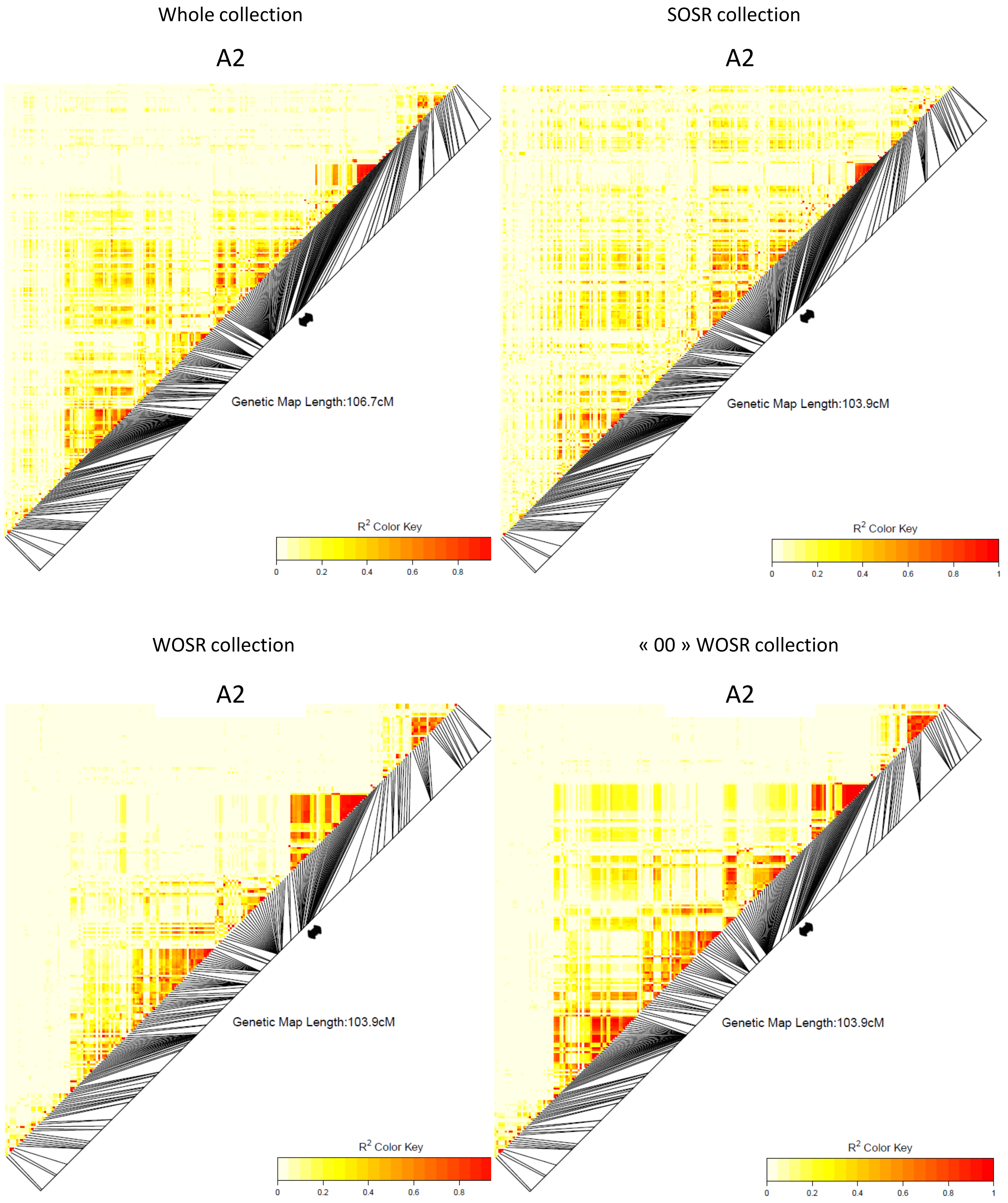
Whole collection
A2
Genetic Map Length:106.7cM
R2 Color Key
0 0.2 0.4 0.6 0.8
SOSR collection
A2
Genetic Map Length:103.9cM
R2 Color Key
0 0.2 0.4 0.6 0.8 1
WOSR collection
A2
Genetic Map Length:103.9cM
R2 Color Key
0 0.2 0.4 0.6 0.8
« 00 » WOSR collection
A2
Genetic Map Length:103.9cM
R2 Color Key
0 0.2 0.4 0.6 0.8 1

Whole collection

A3

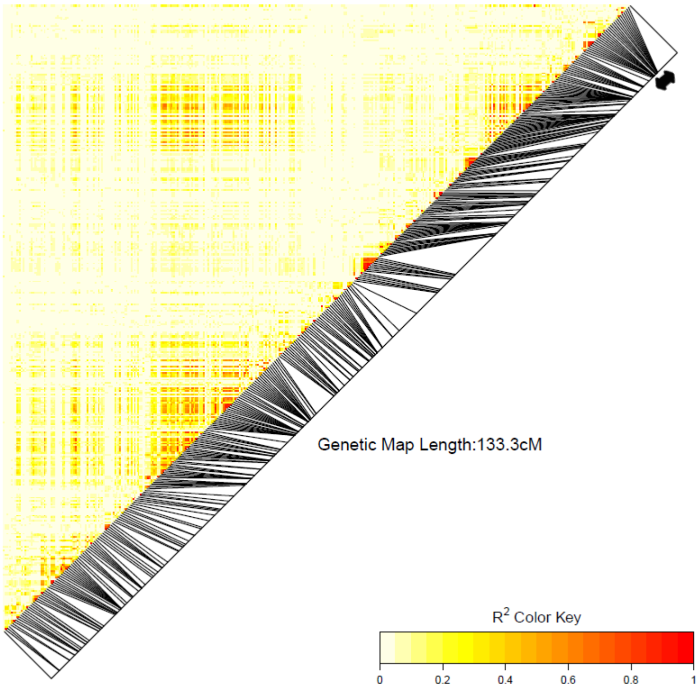

SOSR collection

A3

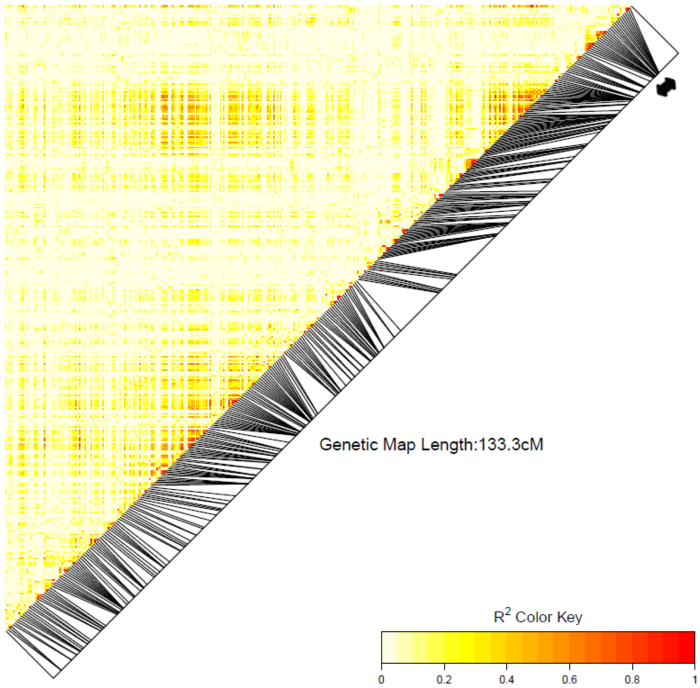

WOSR collection

A3

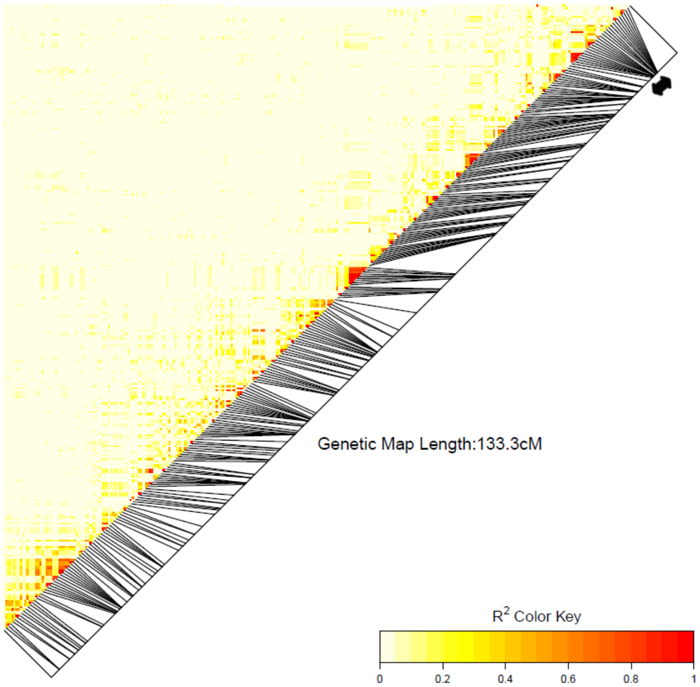

« 00 » WOSR collection

A3

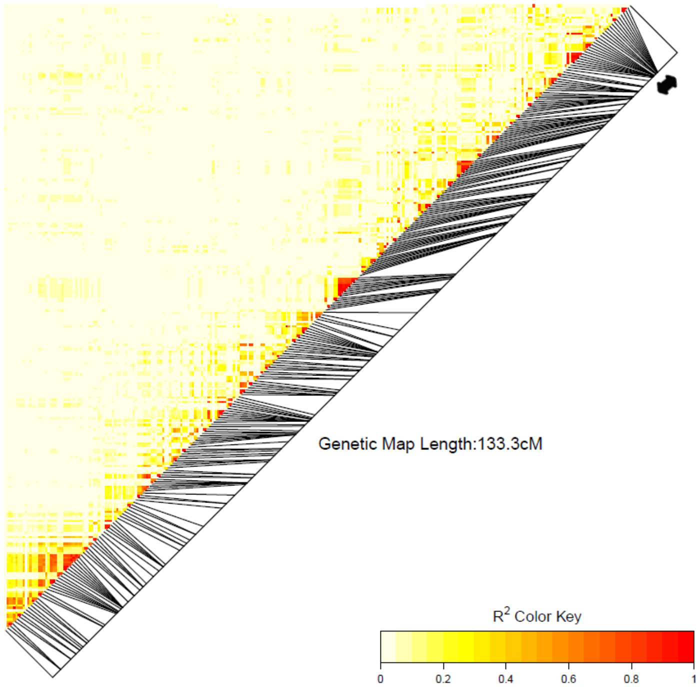

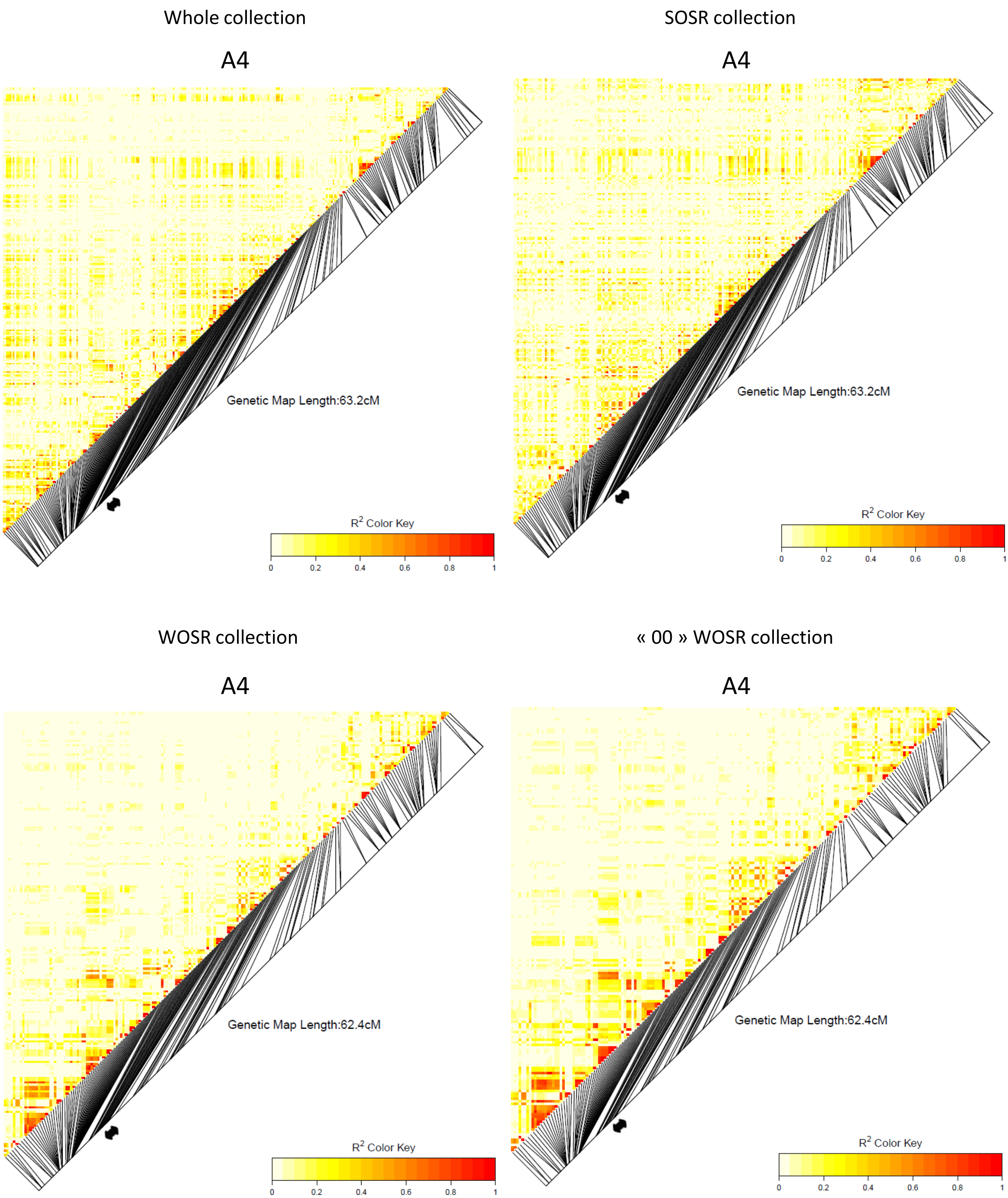

Whole collection
A4
Genetic Map Length:63.2cM
R2 Color Key
0 0.2 0.4 0.6 0.8 1
SOSR collection
A4
Genetic Map Length:63.2cM
R2 Color Key
0 0.2 0.4 0.6 0.8 1
WOSR collection
A4
Genetic Map Length:62.4cM
R2 Color Key
0 0.2 0.4 0.6 0.8 1
« 00 » WOSR collection
A4
Genetic Map Length:62.4cM
R2 Color Key
0 0.2 0.4 0.6 0.8 1

Whole collection

A5

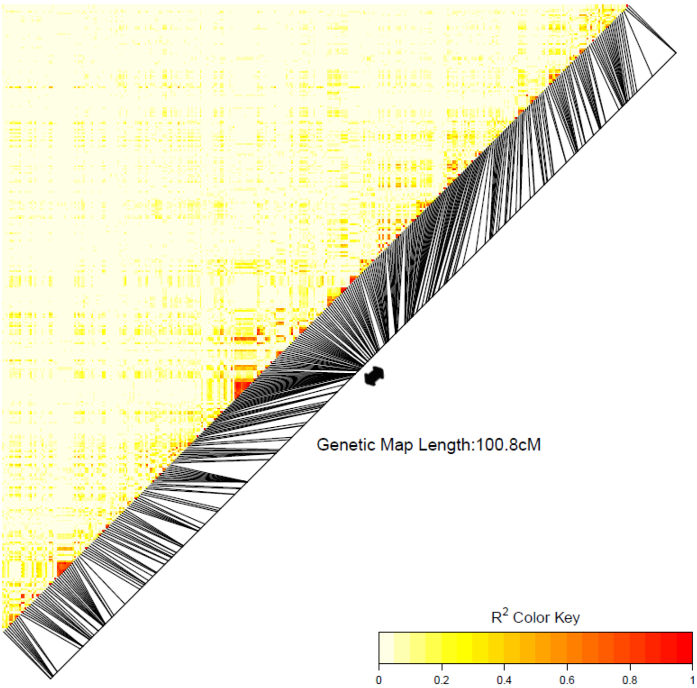

SOSR collection

A5

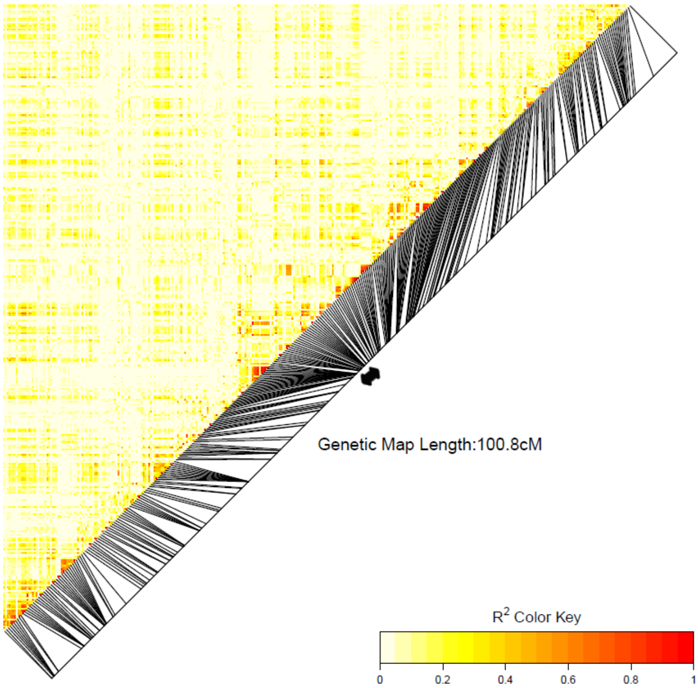

WOSR collection

A5

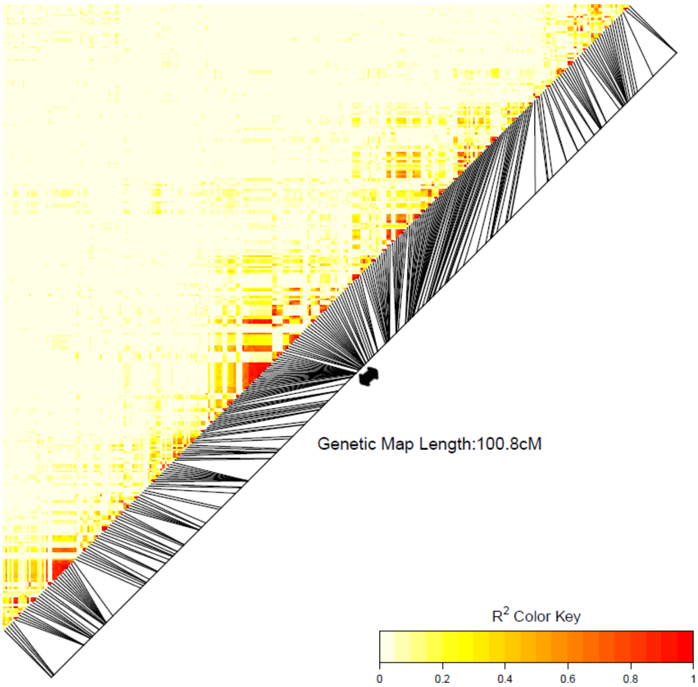

« 00 » WOSR collection

A5

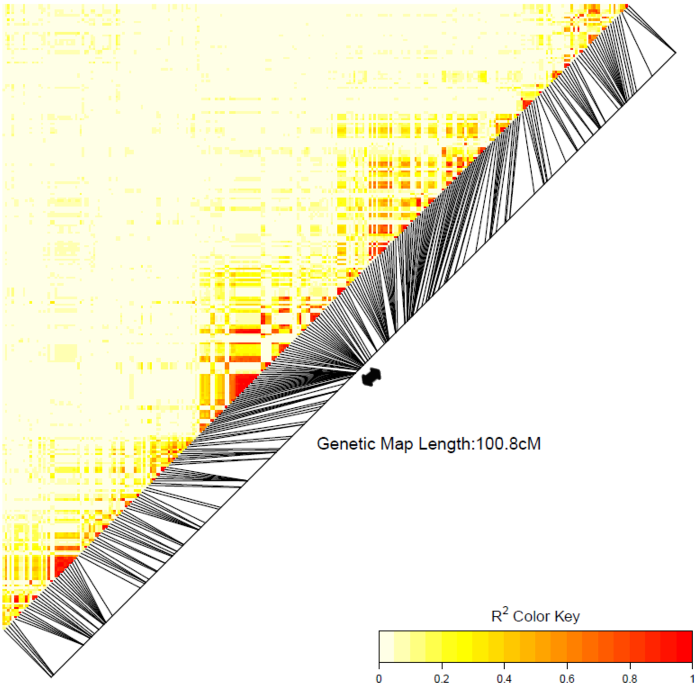

Whole collection

A6

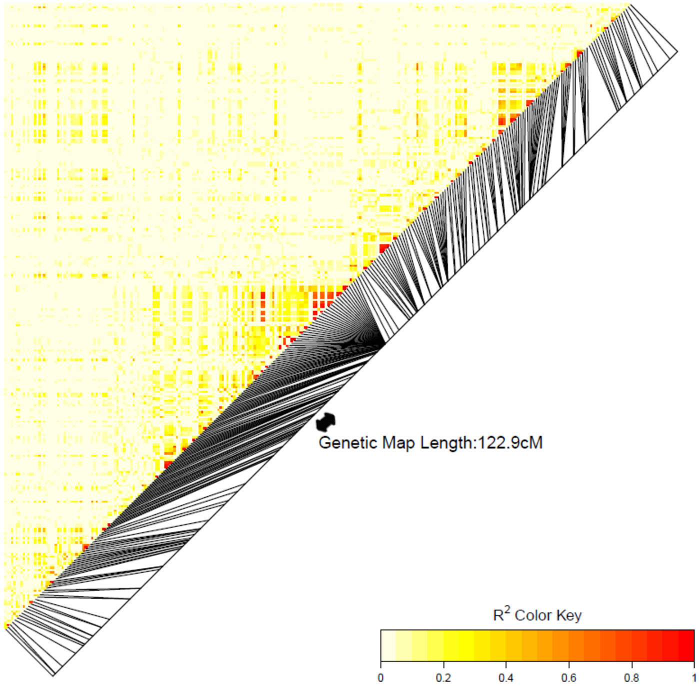

SOSR collection

A6

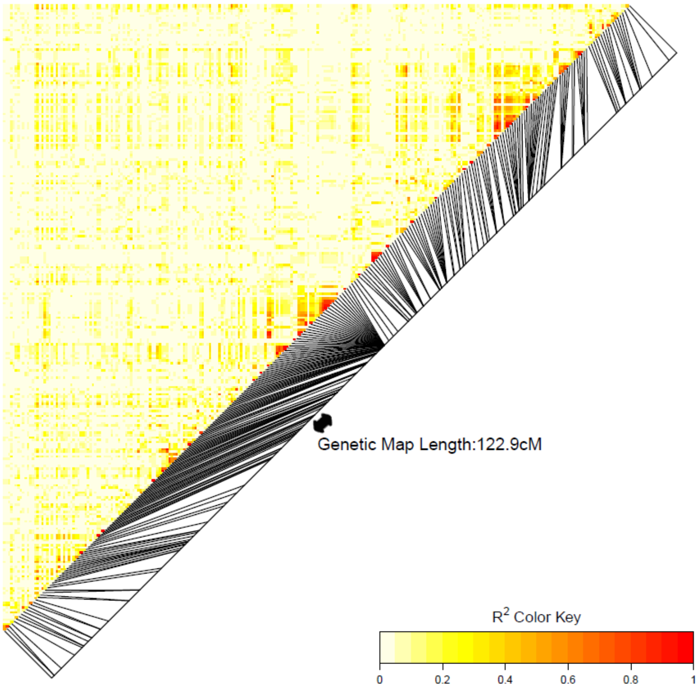

WOSR collection

A6

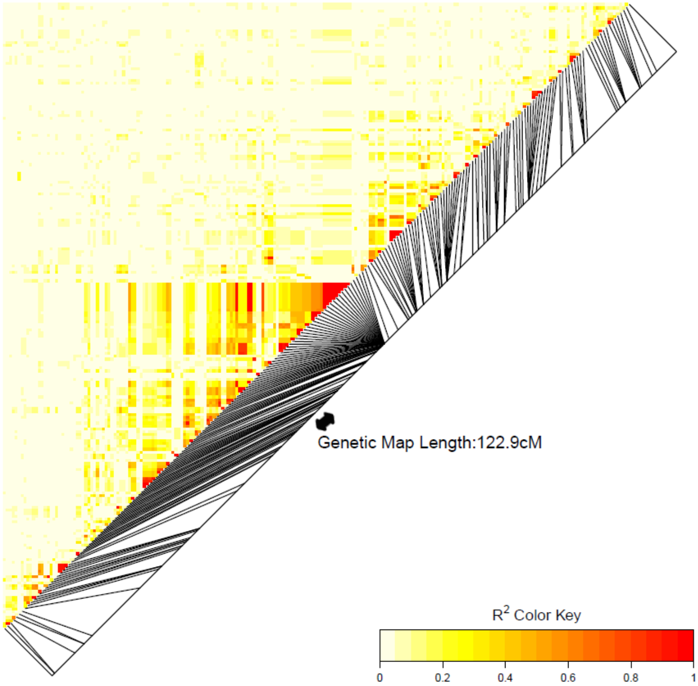

« 00 » WOSR collection

A6

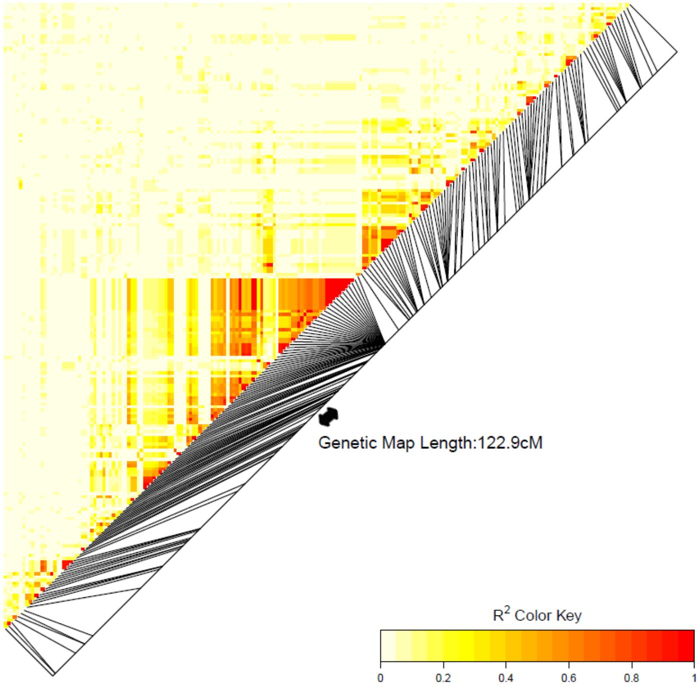

Whole collection

A7

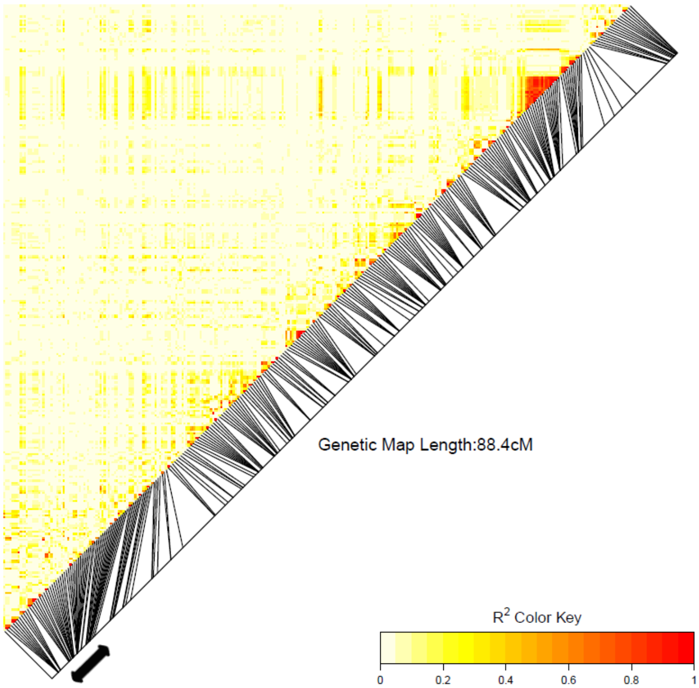

SOSR collection

A7

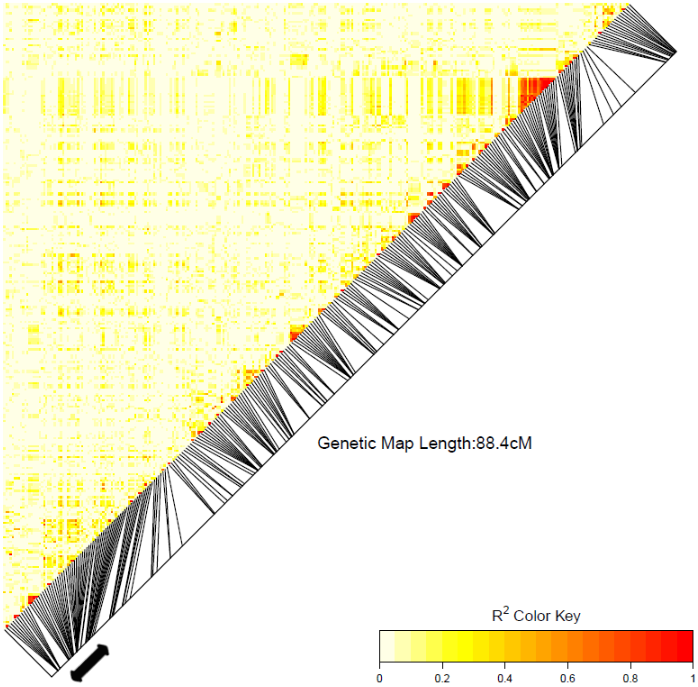

WOSR collection

A7

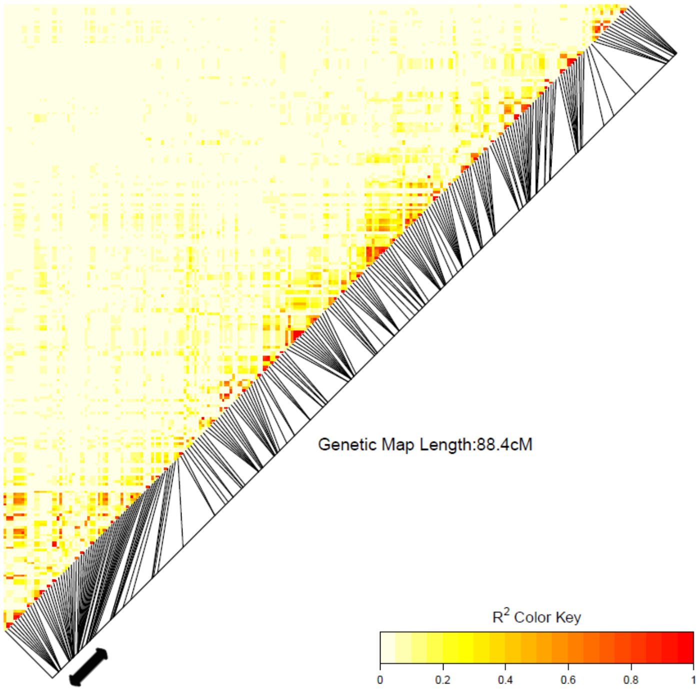

« 00 » WOSR collection

A7

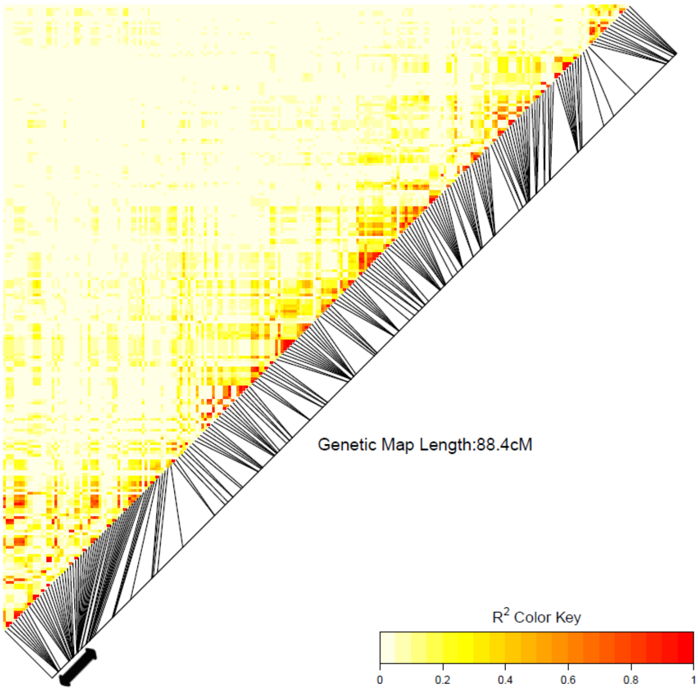

Whole collection

A8

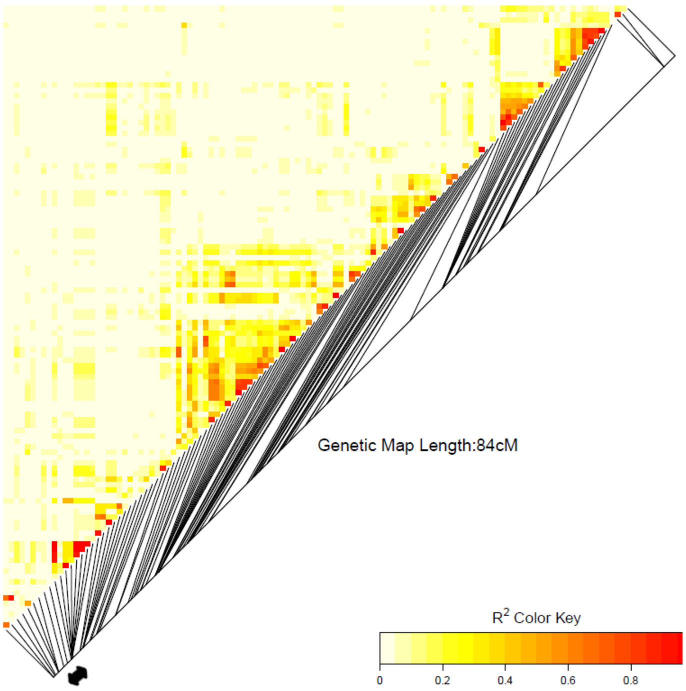

SOSR collection

A8

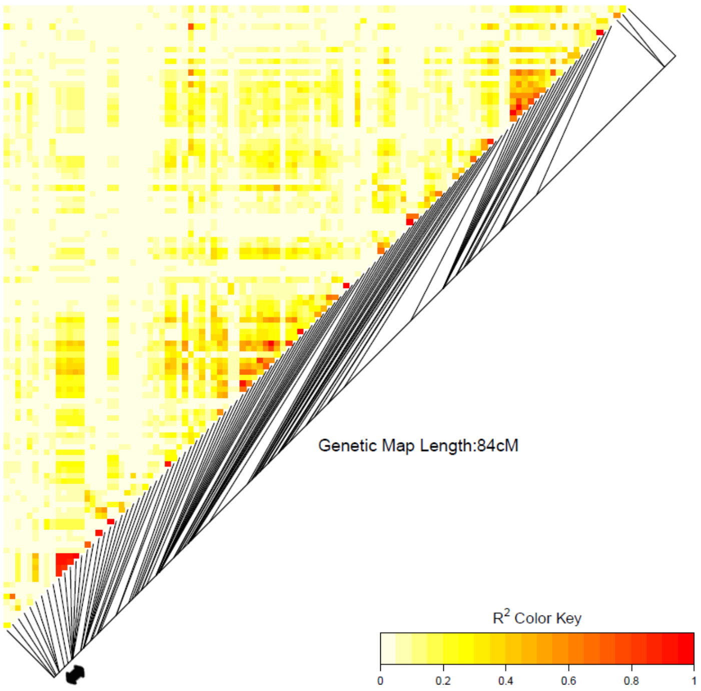

WOSR collection

A8

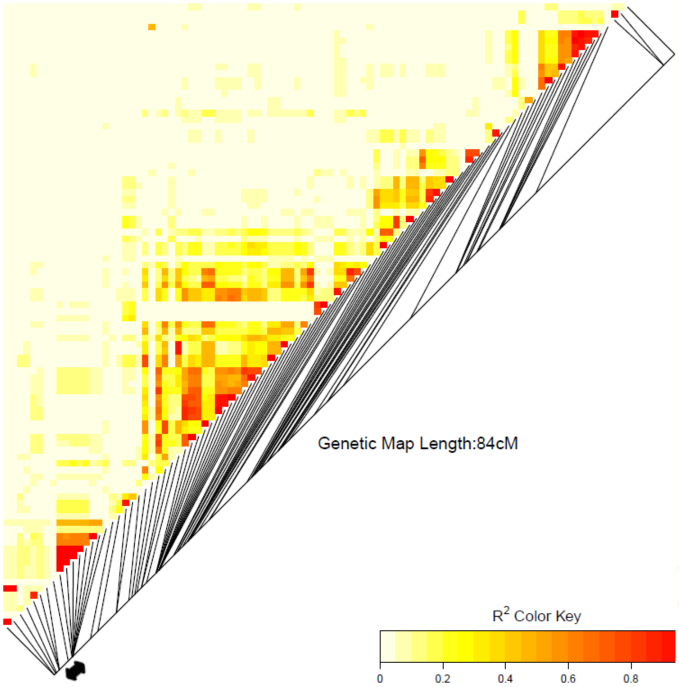

« 00 » WOSR collection

A8

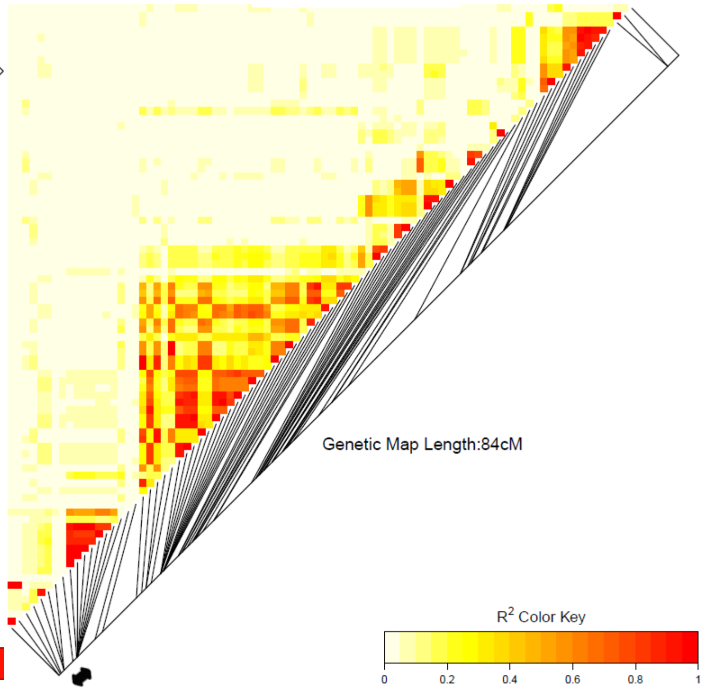

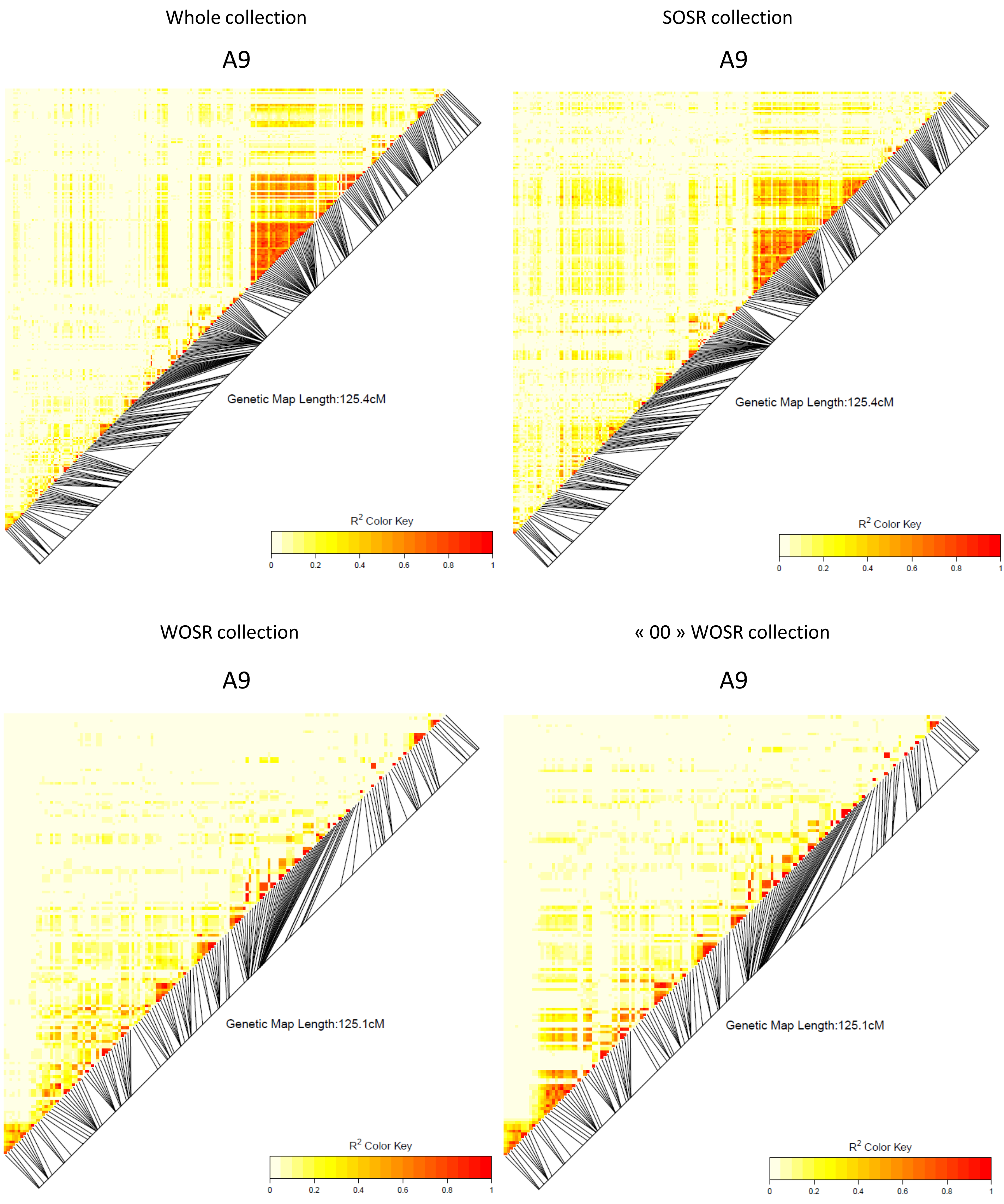
Whole collection
A9
Genetic Map Length:125.4cM
R2 Color Key
0
0.2
0.4
0.6
0.8
1
SOSR collection
A9
Genetic Map Length:125.4cM
R2 Color Key
0
0.2
0.4
0.6
0.8
1
WOSR collection
A9
Genetic Map Length:125.1cM
R2 Color Key
0
0.2
0.4
0.6
0.8
1
« 00 » WOSR collection
A9
Genetic Map Length:125.1cM
R2 Color Key
0
0.2
0.4
0.6
0.8
1

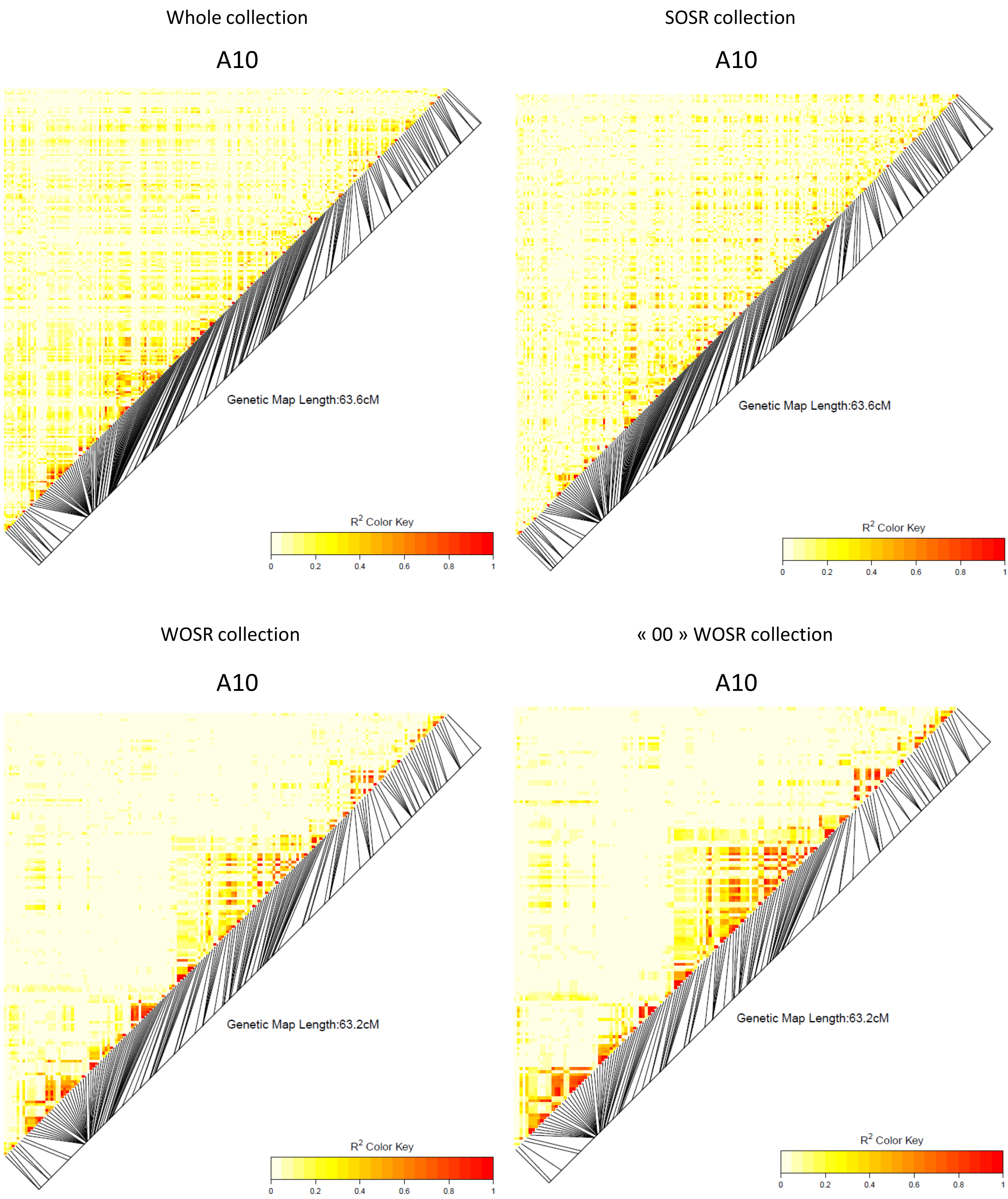

Whole collection
A10
Genetic Map Length:63.6cM
R2 Color Key
0
0.2
0.4
0.6
0.8
1
SOSR collection
A10
Genetic Map Length:63.6cM
R2 Color Key
0
0.2
0.4
0.6
0.8
1
WOSR collection
A10
Genetic Map Length:63.2cM
R2 Color Key
0
0.2
0.4
0.6
0.8
1
« 00 » WOSR collection
A10
Genetic Map Length:63.2cM
R2 Color Key
0
0.2
0.4
0.6
0.8
1

Whole collection

C1

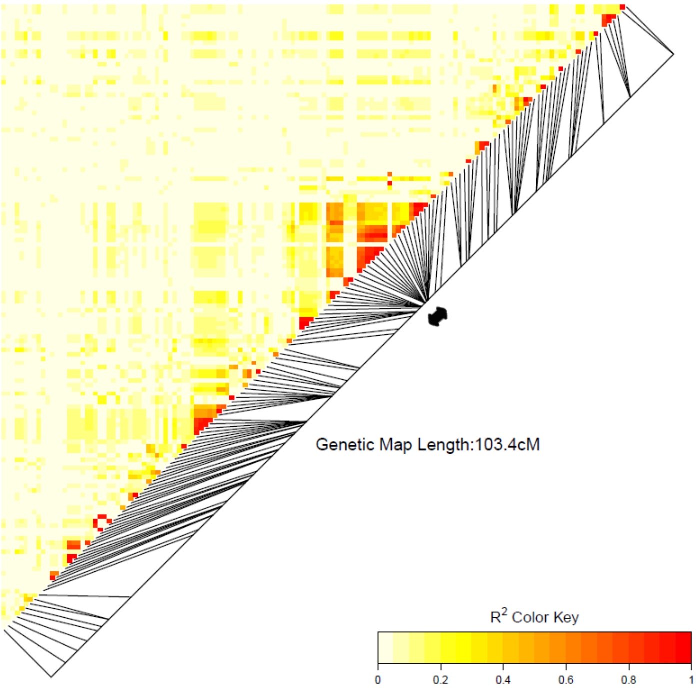

SOSR collection

C1

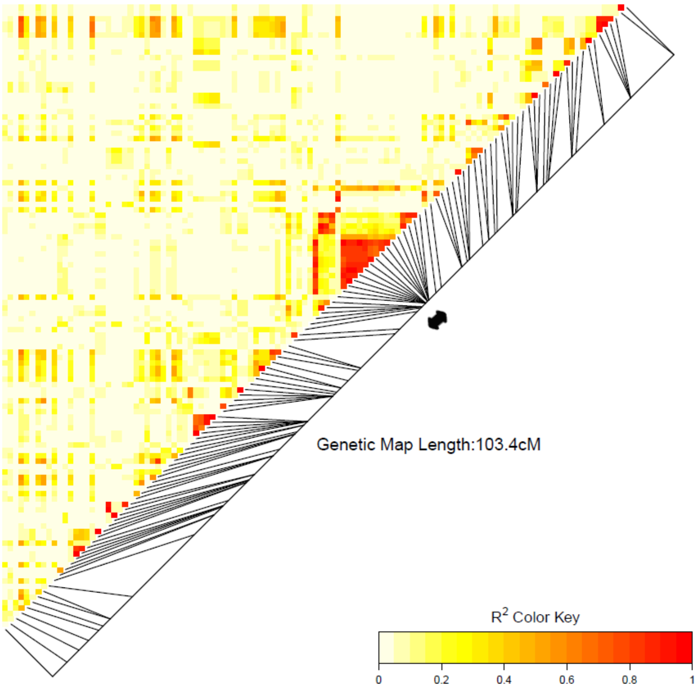

WOSR collection

C1

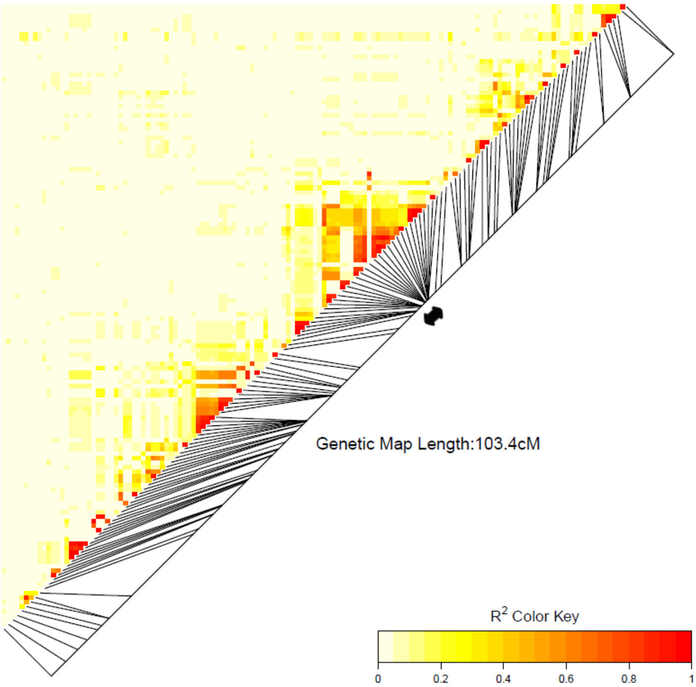

« 00 » WOSR collection

C1

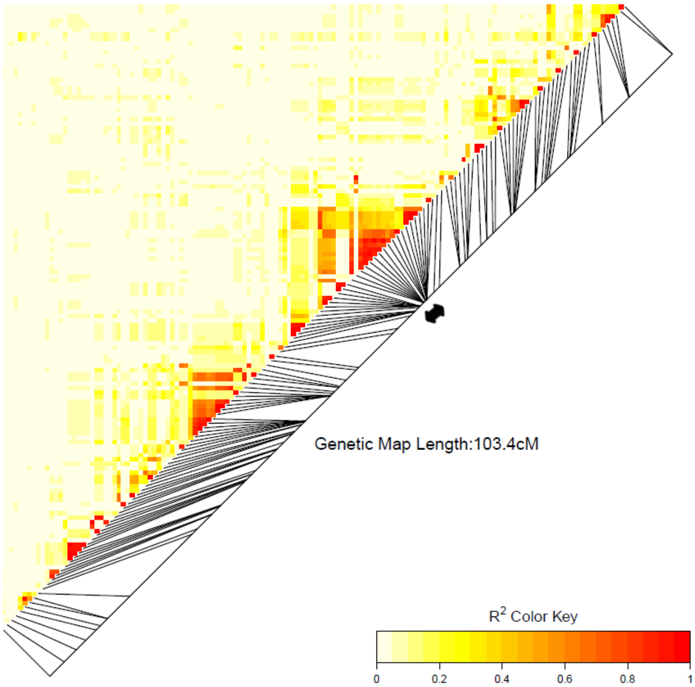

Whole collection

C2

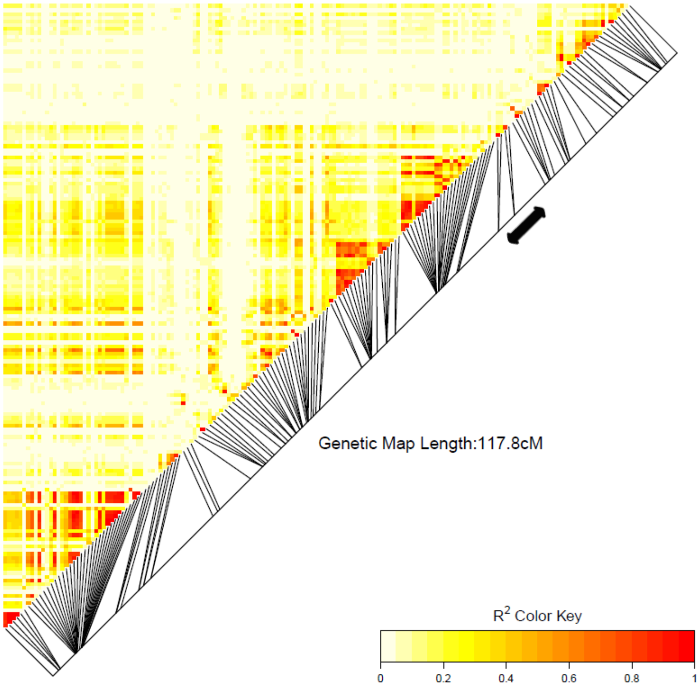

SOSR collection

C2

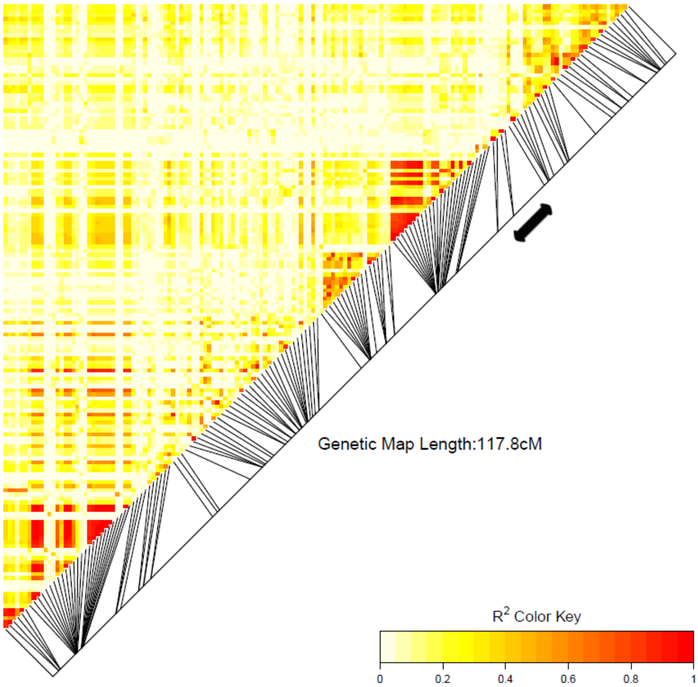

WOSR collection

C2

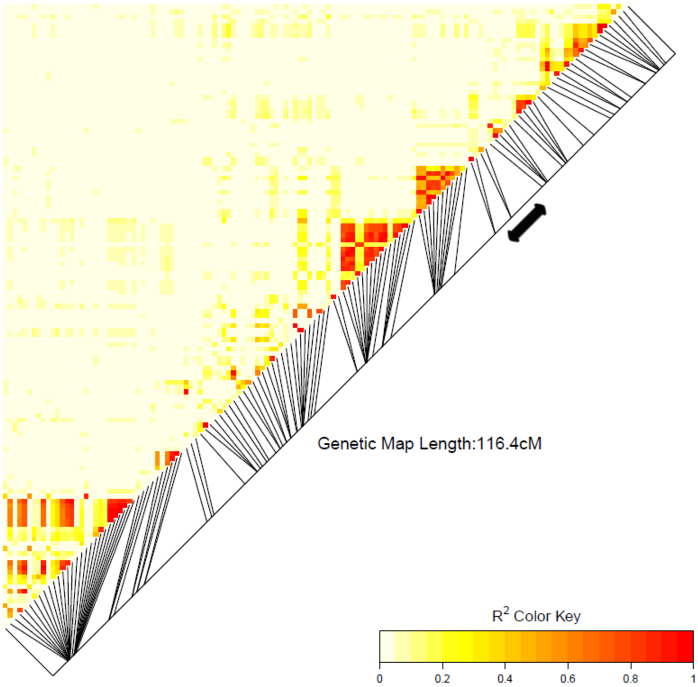

« 00 » WOSR collection

C2

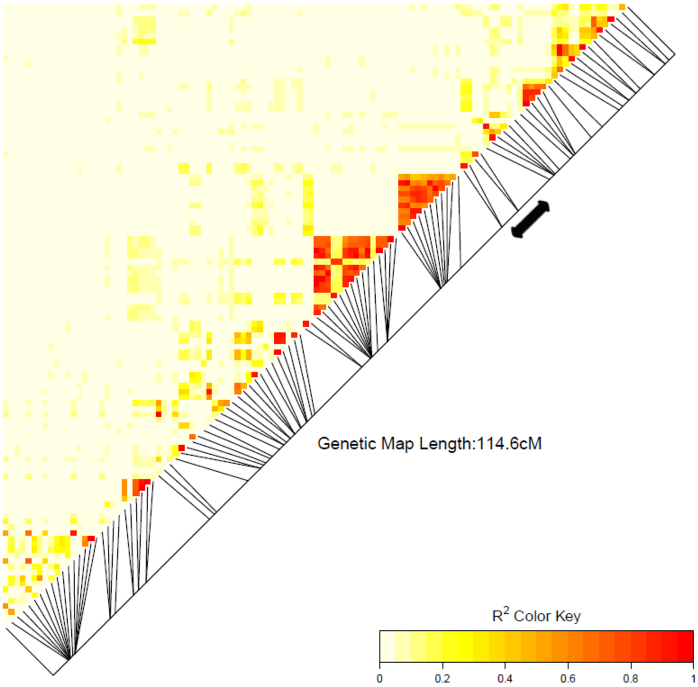

Whole collection

C3

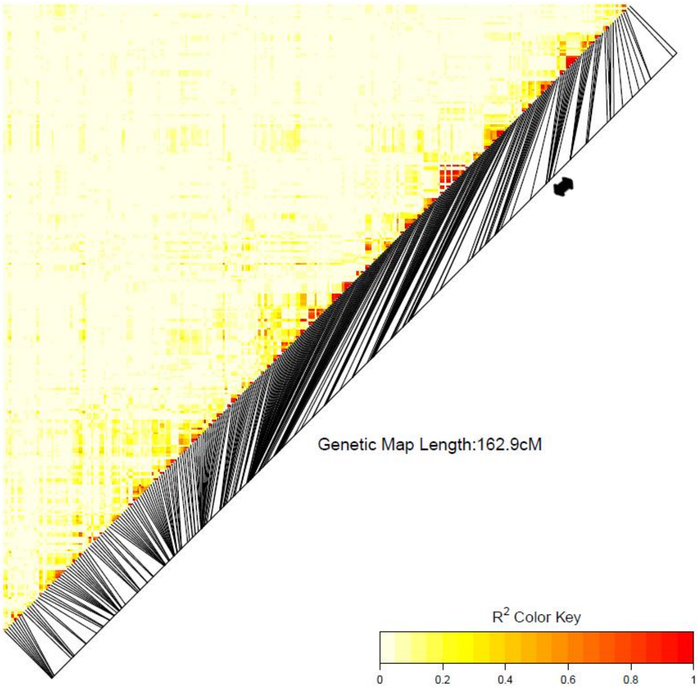

SOSR collection

C3

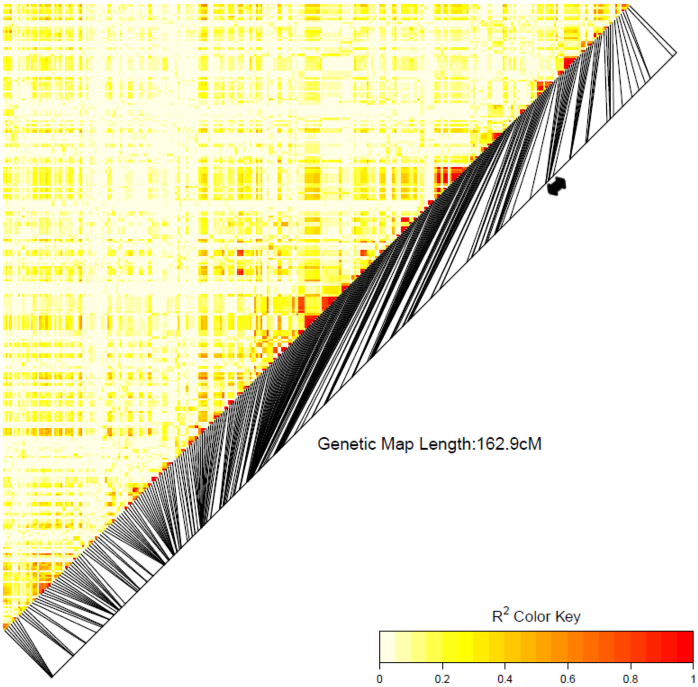

WOSR collection

C3

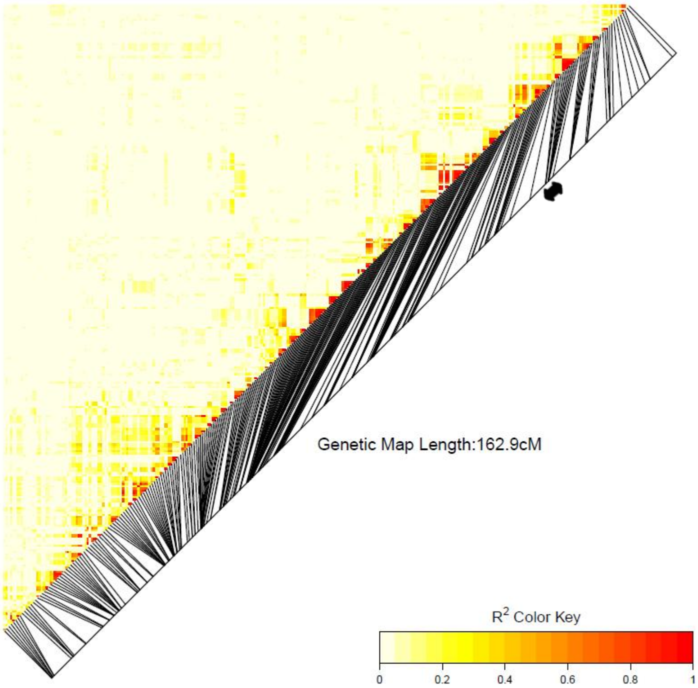

« 00 » WOSR collection

C3

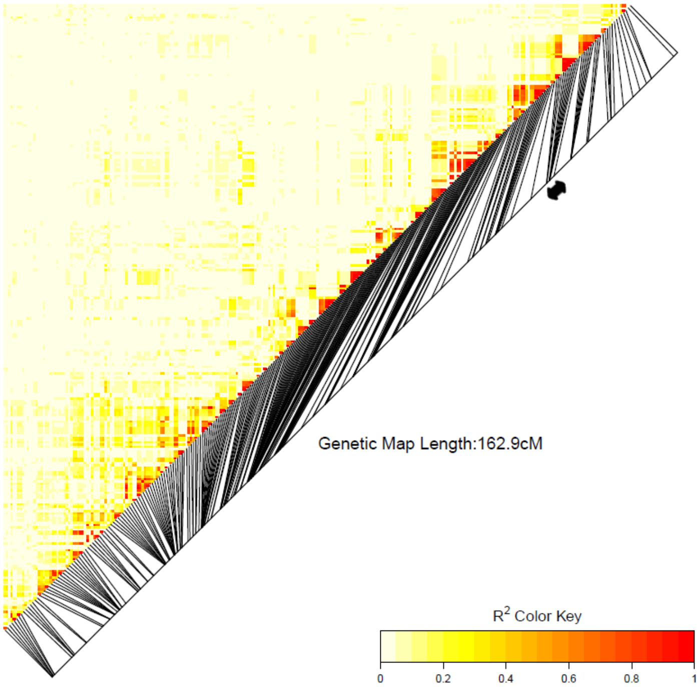

Whole collection

C4

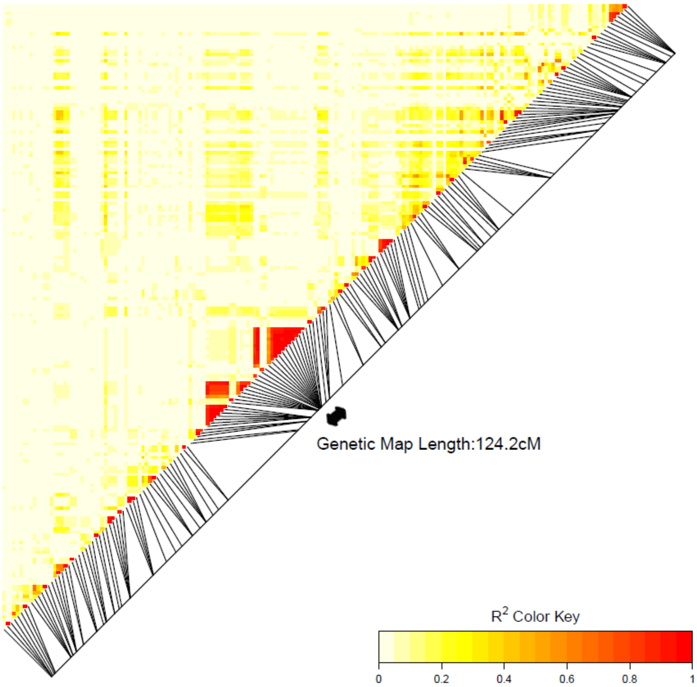

SOSR collection

C4

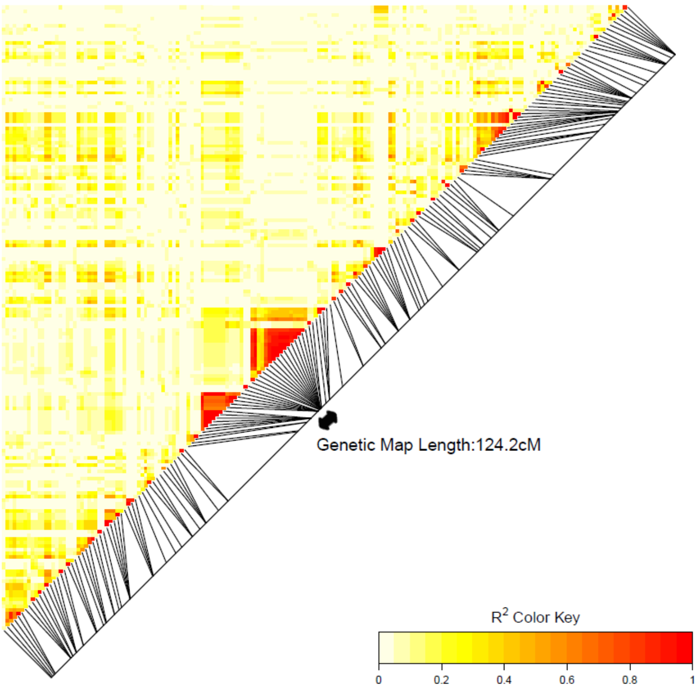

WOSR collection

C4

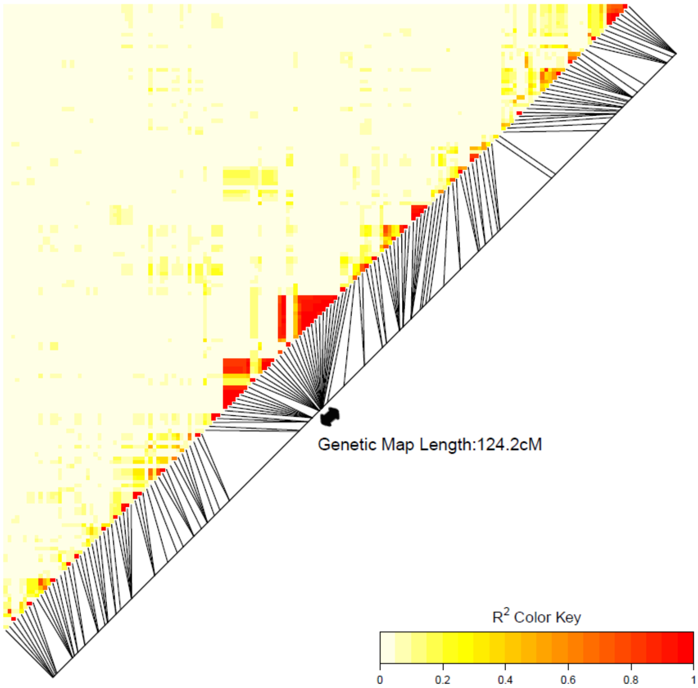

« 00 » WOSR collection

C4

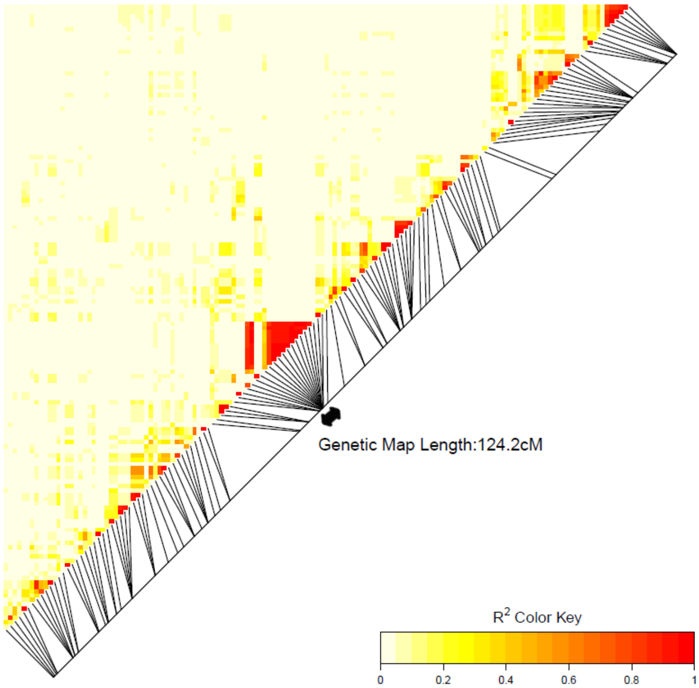

Whole collection

C5

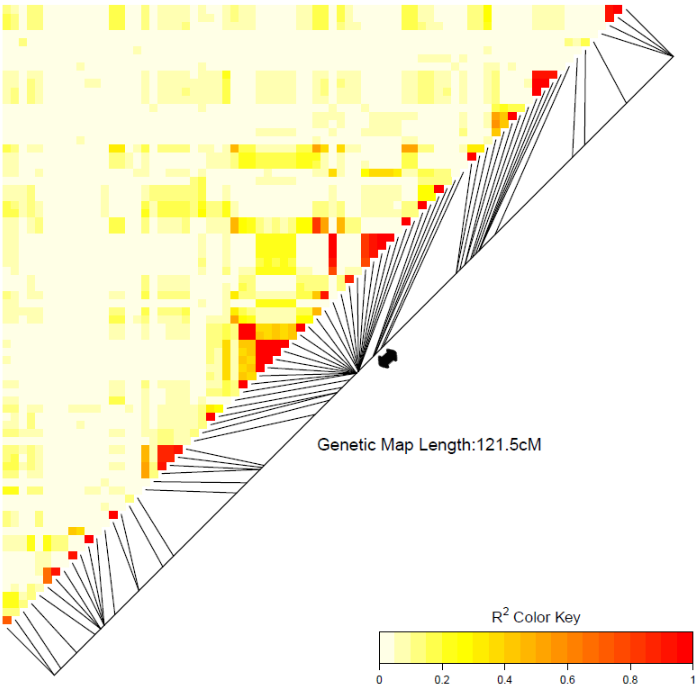

SOSR collection

C5

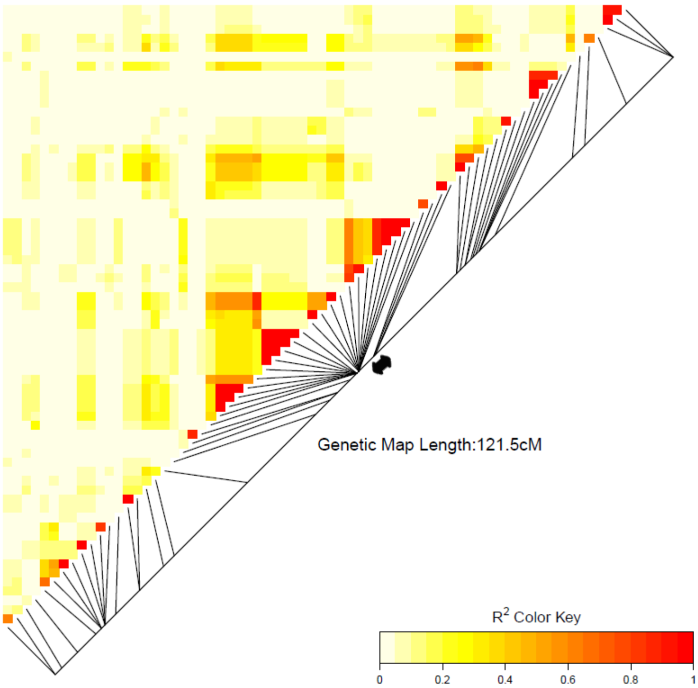

WOSR collection

C5

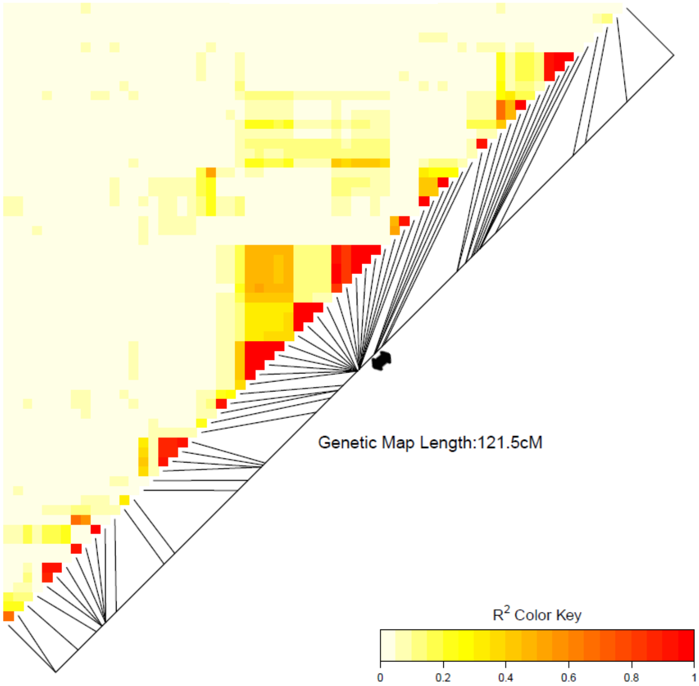

« 00 » WOSR collection

C5

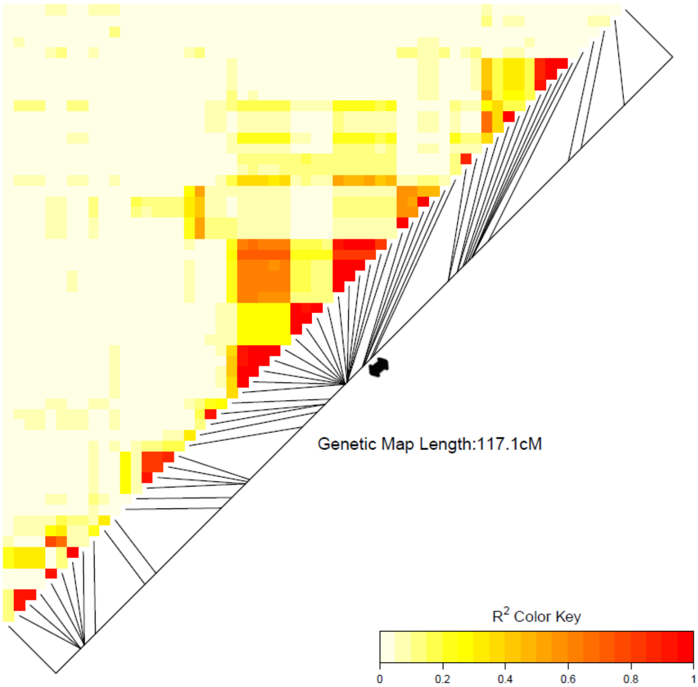

Whole collection

C6

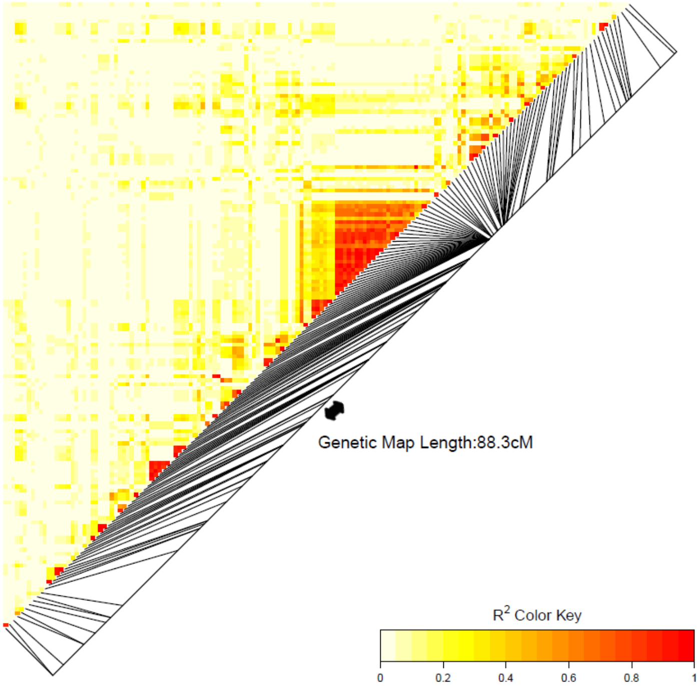

SOSR collection

C6

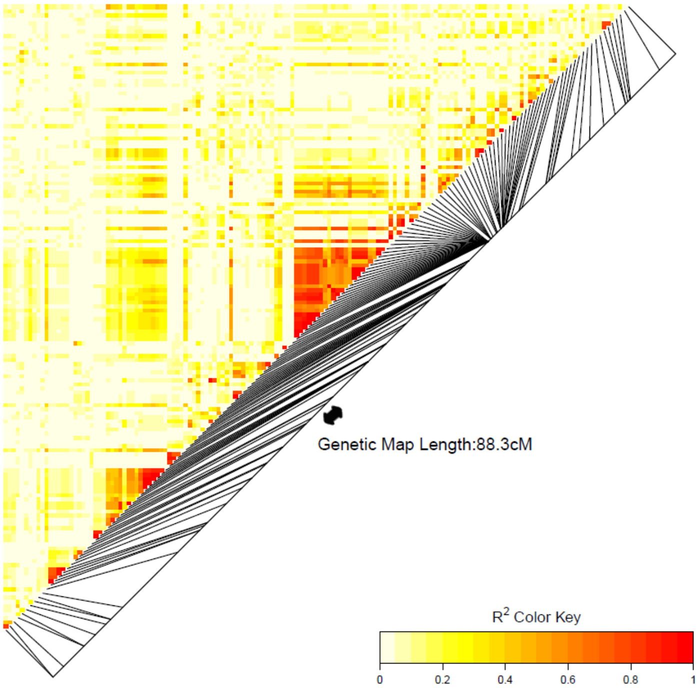

WOSR collection

C6

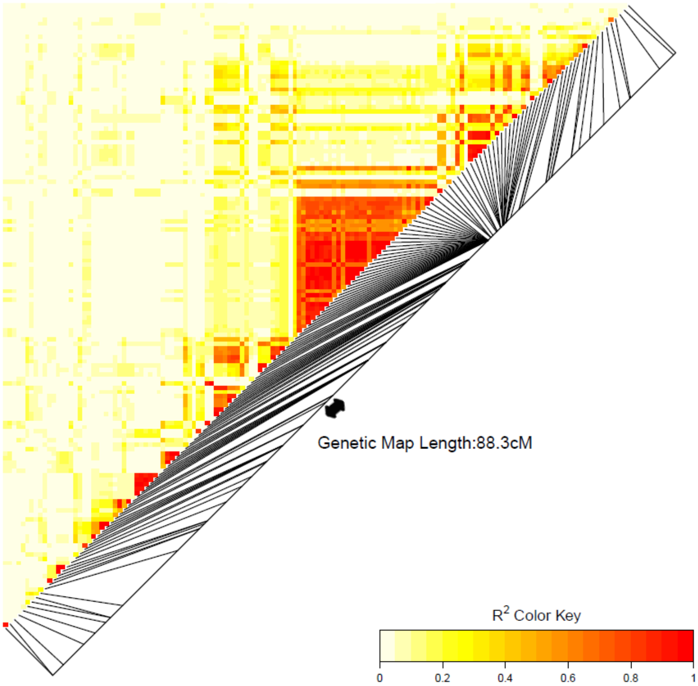

« 00 » WOSR collection

C6

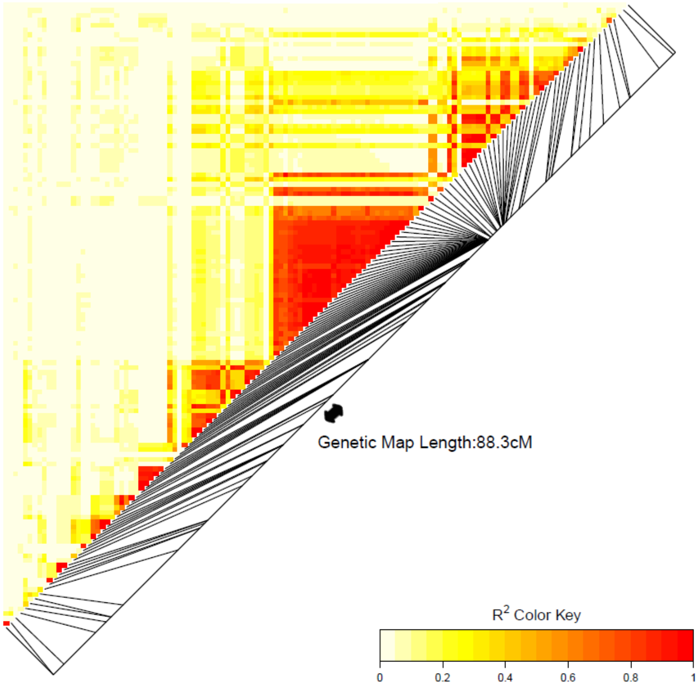

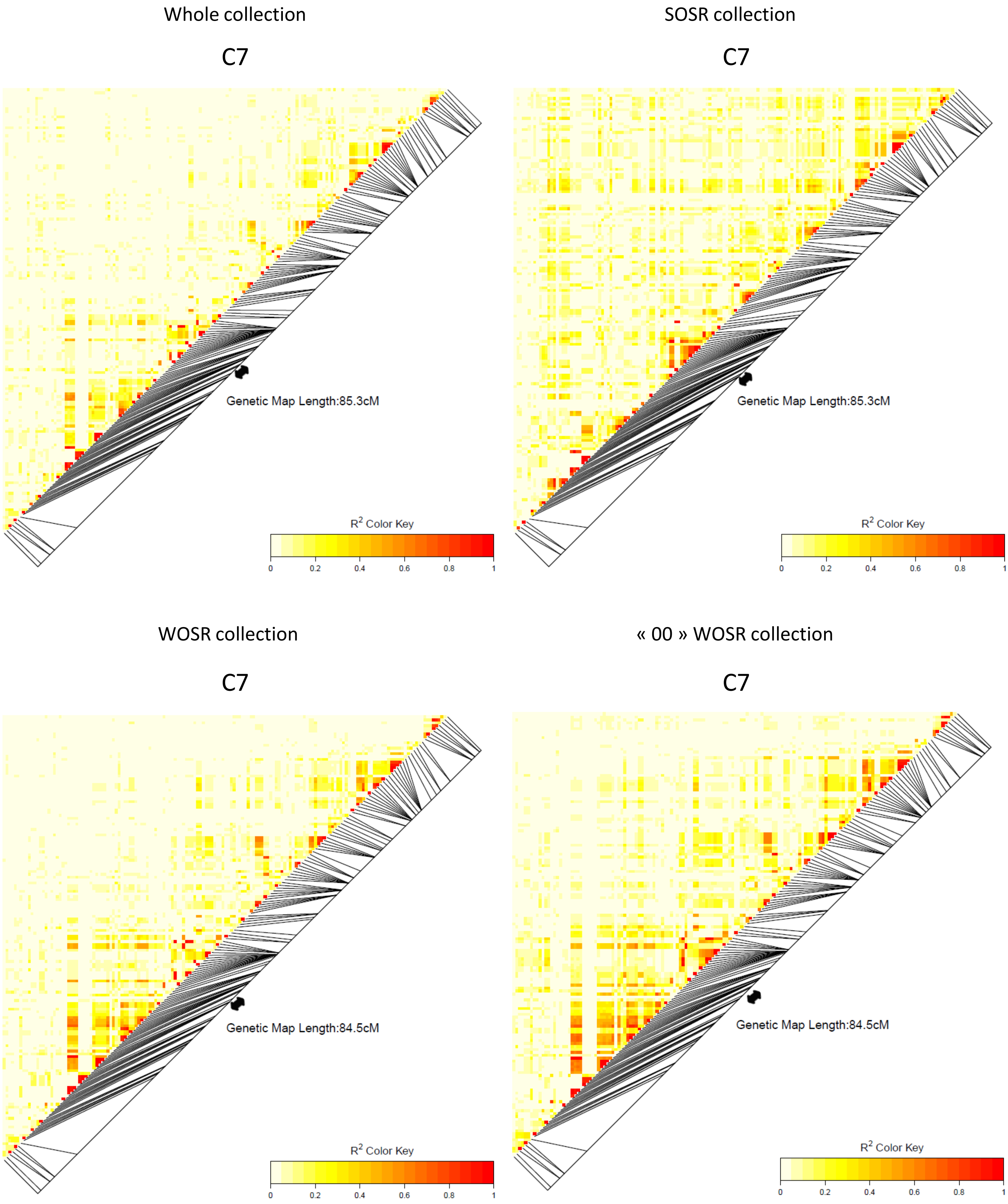

Whole collection
C7
Genetic Map Length:85.3cM
R2 Color Key
0
0.2
0.4
0.6
0.8
1
SOSR collection
C7
Genetic Map Length:85.3cM
R2 Color Key
0
0.2
0.4
0.6
0.8
1
WOSR collection
C7
Genetic Map Length:84.5cM
R2 Color Key
0
0.2
0.4
0.6
0.8
1
« 00 » WOSR collection
C7
Genetic Map Length:84.5cM
R2 Color Key
0
0.2
0.4
0.6
0.8
1

Whole collection

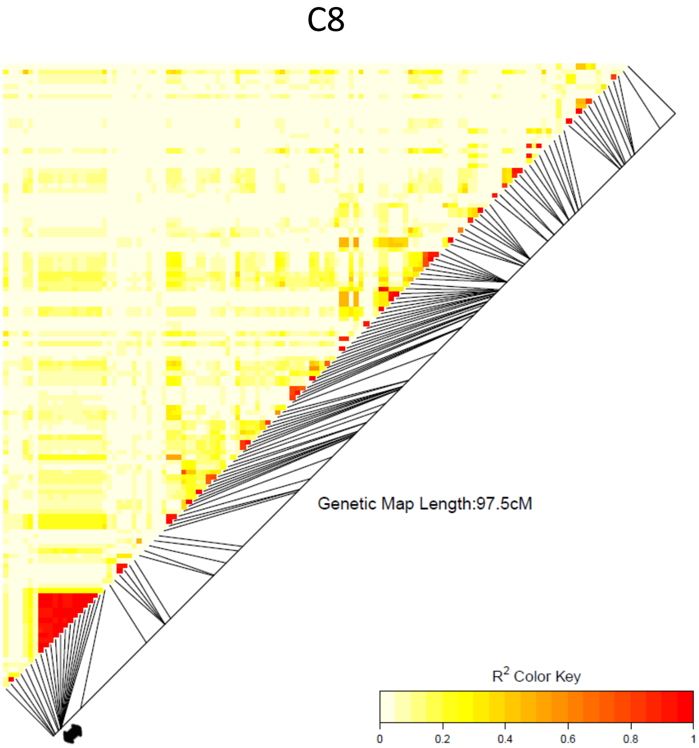

SOSR collection

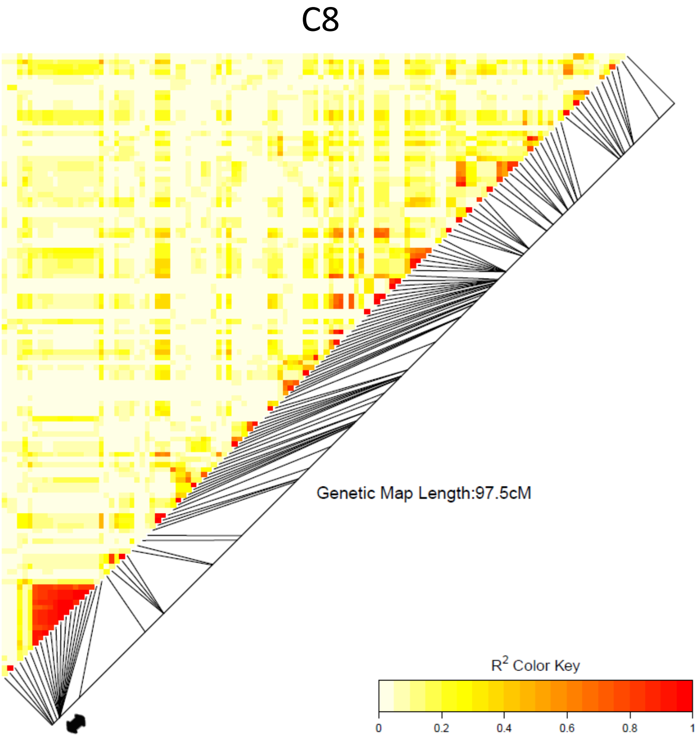

WOSR collection

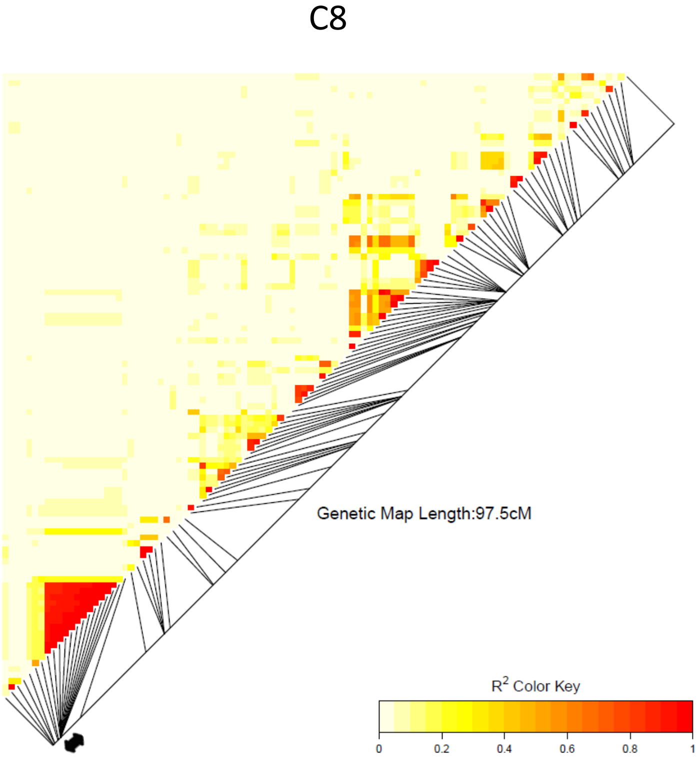

« 00 » WOSR collection

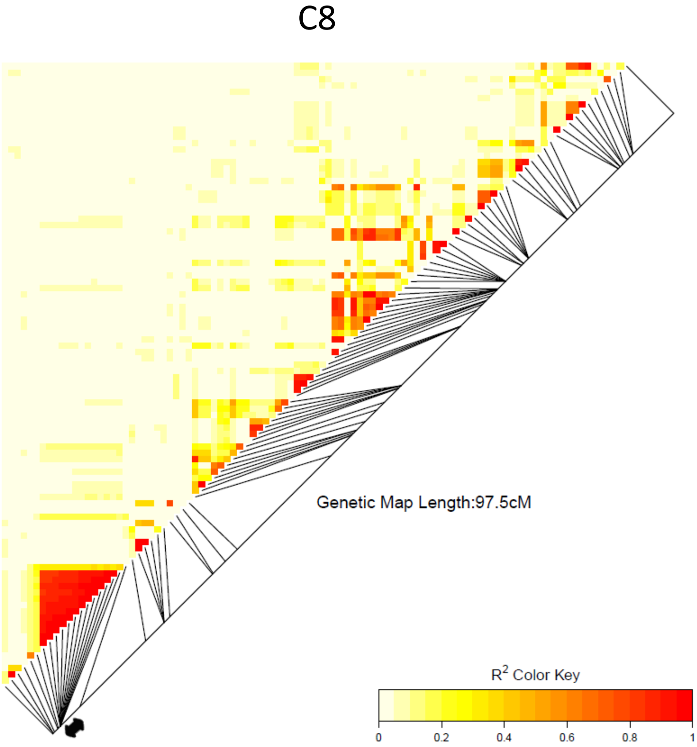

Whole collection

C9

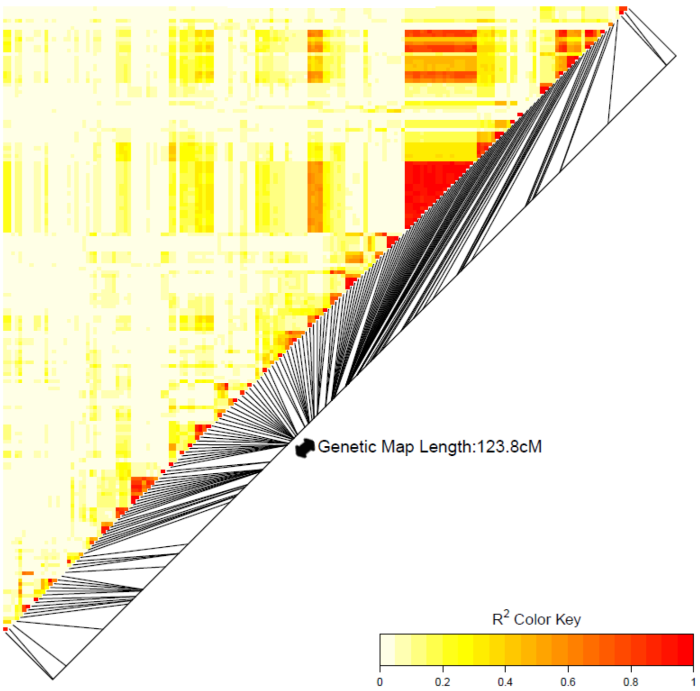

SOSR collection

C9

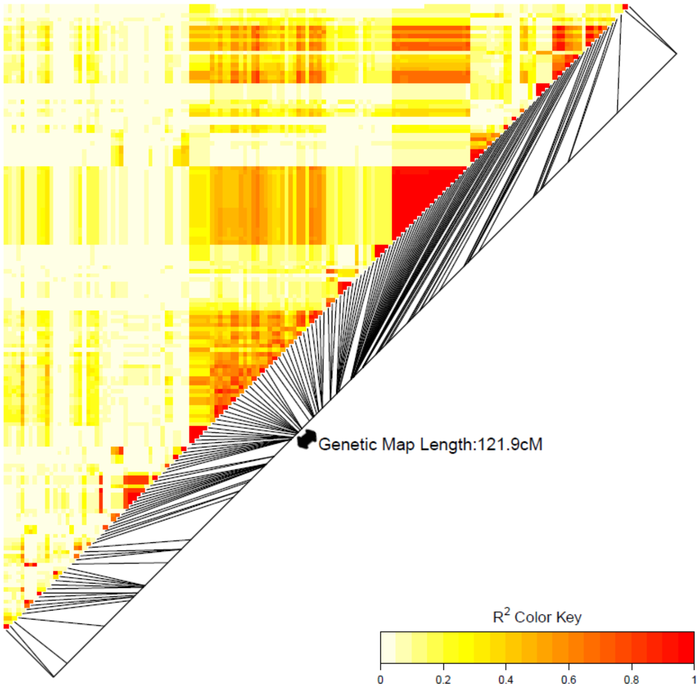

WOSR collection

C9

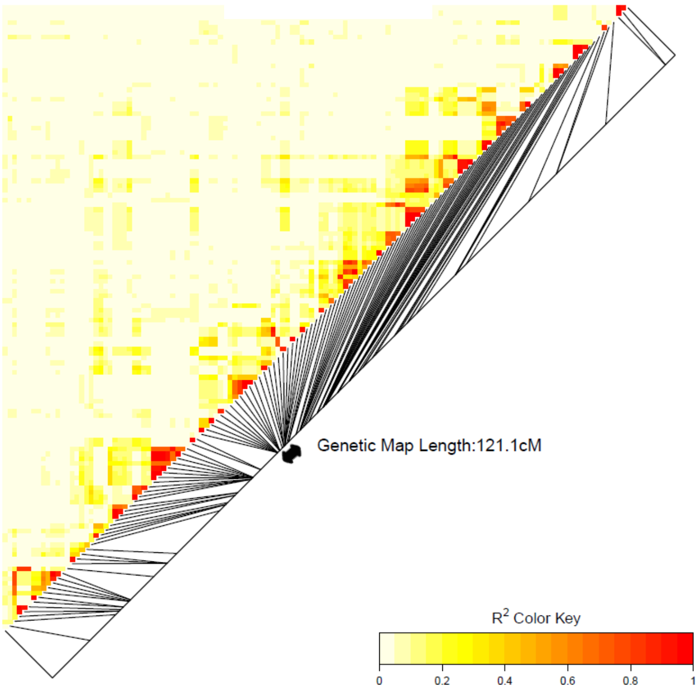

« 00 » WOSR collection

C9

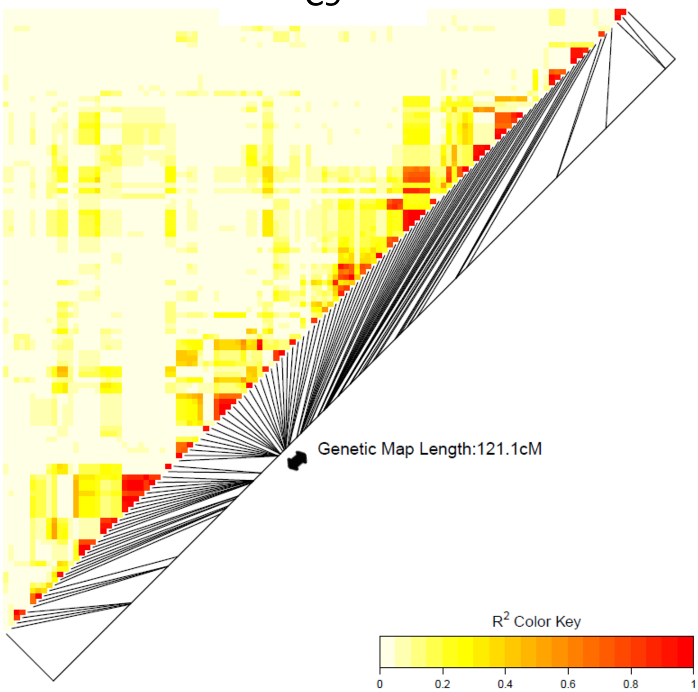

Supplement: Additional file 9: Figure S5 — Linkage disequilibrium heatmaps for each linkage group in the whole, spring (SOSR), winter (WOSR) and “00” WOSR collections. The putative position of the centromeres is indicated by a black arrow. [file 1471-2164-14-120-S9.pdf]
